# Using single-sample networks to identify the contrasting patterns of gene interactions and reveal the radiation dose-dependent effects in multiple tissues of spaceflight mice

**DOI:** 10.1038/s41526-024-00383-7

**Published:** 2024-04-04

**Authors:** Yan Zhang, Lei Zhao, Yeqing Sun

**Affiliations:** Institute of Environmental Systems Biology, College of Environmental Science and Engineering, Dalian Maritime University, 116026 Dalian, Liaoning China

**Keywords:** Computational biology and bioinformatics, Systems biology, Molecular biology, Risk factors

## Abstract

Transcriptome profiles are sensitive to space stressors and serve as valuable indicators of the biological effects during spaceflight. Herein, we transformed the expression profiles into gene interaction patterns by single-sample networks (SSNs) and performed the integrated analysis on the 301 spaceflight and 290 ground control samples, which were obtained from the GeneLab platform. Specifically, an individual SSN was established for each sample. Based on the topological structures of 591 SSNs, the differentially interacted genes (DIGs) were identified between spaceflights and ground controls. The results showed that spaceflight disrupted the gene interaction patterns in mice and resulted in significant enrichment of biological processes such as protein/amino acid metabolism and nucleic acid (DNA/RNA) metabolism (*P*-value < 0.05). We observed that the mice exposed to radiation doses within the three intervals (4.66–7.14, 7.592–8.295, 8.49–22.099 mGy) exhibited similar gene interaction patterns. Low and medium doses resulted in changes to the circadian rhythm, while the damaging effects on genetic material became more pronounced in higher doses. The gene interaction patterns in response to space stressors varied among different tissues, with the spleen, lung, and skin being the most responsive to space radiation (*P*-value < 0.01). The changes observed in gene networks during spaceflight conditions might contribute to the development of various diseases, such as mental disorders, depression, and metabolic disorders, among others. Additionally, organisms activated specific gene networks in response to virus reactivation. We identified several hub genes that were associated with circadian rhythms, suggesting that spaceflight could lead to substantial circadian rhythm dysregulation.

## Introduction

As space exploration advances, the health risks of astronauts induced by the space environment are extensively concerning^1^. Exposure to space radiation and microgravity are primary hazards to astronauts’ health in long-duration space missions^2^, where space radiation is mainly composed of high energy protons produced from solar particle events (SPE), heavy ions originated from galactic cosmic rays (GCR), and secondary particles generated through interactions with spacecraft shielding^3^. Previous studies have determined that the frequency of chromosome aberrations is substantially increased after spaceflight when compared to preflight levels^4^. The change is even more pronounced among individuals who have undergone long-term flights, which indicates that the space environment can induce substantial DNA damage^5,6^. Apart from the known risk of increased cancer from spaceflight^7^, astronauts returning from the International Space Station (ISS) contend with a range of health issues, including bone and muscle mass loss, central nervous system problems, immune dysfunction, and cardiovascular issues, etc.^8^. Therefore, it is necessary to employ advanced analytical methods to further reveal the effects of spaceflight on organisms.

The accumulation of omics data has opened up new possibilities for gaining a comprehensive understanding of the processes of molecular changes induced by spaceflight. Overall, pathway analysis on the multi-omics datasets showed substantial enrichment for mitochondrial processes, innate immunity, chronic inflammation, cell cycle, circadian rhythm, and olfactory functions^9^. The NASA twins study analyzed the omics data to find that some changes persisted 6 months after astronauts returned to Earth, including some genes’ expression levels^2^. McDonald et al. performed gene set enrichment analysis (GSEA) on multiple omics datasets from spaceflight experiments in GeneLab and found the variation of different biological signatures (functions) with radiation dose^10^. They discovered changes in mitochondrial function, ribosomal assembly, and immune pathways as a function of dose^10^. However, one issue with current commonly used omics analyses is that when multiple datasets are incorporated, the results may be impacted by data heterogeneity. Therefore, it is a challenge to mine the patterns of gene changes from spaceflight samples under different exposure conditions.

There is a growing recognition that phenotypic changes in organisms, which are often driven by complex gene networks, cannot be fully explained by each isolated gene^11^. It holds crucial significance for investigating the mechanisms induced by space stressors from molecular networks and deciphering how gene interactions change during spaceflight. Generally, the gene networks are built from multiple samples, neglecting individual-specific information^12^. To address this issue, the single-sample network (SSN) has gained notable research interest in recent years. This approach entails the creation of a network for each individual sample, wherein genes are represented as nodes, and their interactions are denoted as edges. When combined with the protein–protein interaction (PPI) network, the SNNs can characterize individual-specific gene interaction patterns. Currently, SSN analysis techniques have been shown as important tools in deciphering complex molecular mechanisms. For example, the application of SSNs to cancer data sourced from the cancer genome atlas (TCGA) enabled the prediction of individual-specific disease genes, the identification of distinct cancer phenotypes, and the subsequent classification of cancer subtypes^12^. Chen et al. employed SSNs to identify cancer-related genes and discovered two possible lung adenocarcinoma (LUAD) subtypes that exhibited distinct clinical features and molecular mechanisms^13^. Other researchers identified four new SSN-based subtypes in breast cancer, which showed strong heterogeneity in terms of prognosis, somatic mutations, phenotypic changes, and enriched pathways^14^. Besides, progress has been made in predicting pre-disease states or critical states using the dynamic network biomarker (DNB) technique developed based on SSN. Xiangtian et al. applied the DNB technique to analyze omics data of H3N2 cohorts^15^. They successfully detected early-warning signals of the influenza infection for each individual both on the occurred time and event in an accurate manner^15^. Chengming et al. employed landscape dynamic network biomarker (l-DNB) analysis to reveal the complicated process of skin response to ultraviolet (UV) irradiation at both molecular and network levels^16^. They discovered a tipping point before the critical transition state during the pigmentation process and identified 13 core DNB genes to detect this tipping point as a network biomarker^16^. From the promising results of the above research, it is evident that SSNs can effectively reflect gene interaction (regulation) relationships. We believe that the application of SSNs will have a positive impact on space biology.

To comprehensively understand the gene interaction patterns within mouse tissues under spaceflight conditions, we integrated 591 spaceflight mouse samples (301 spaceflight and 290 ground control samples) from 30 datasets in NASA’s GeneLab platform^17,18^ and further constructed an SSN for each individual. Specifically, we combined transcriptome with protein interactome and employed "linear interpolation to obtain network estimates for single samples (LIONESS)" to construct a set of reliable SSNs. LIONESS is a widely used method to reverse engineer SSNs from aggregate networks^11^. Next, we analyzed contrasting patterns of gene interactions between spaceflights and ground controls, especially for gene interaction patterns in ten tissues (adrenal glands, colon, eye, kidney, liver, lung, muscle, skin, spleen, and thymus). Moreover, we examined the effects of radiation dose levels on the gene interaction networks. The potential diseases induced by spaceflight were also predicted. We identified hub genes in the differentially interacted network (DIN) and analyzed the distinctions in the gene interaction networks between spaceflights and ground controls activated by hub genes. This work employs a multi-omics (transcriptome and protein interactome) single-sample network analysis to comprehensively delineate the contrasting patterns of gene interactions between spaceflights and ground controls, which makes a contribution to personalized aerospace medicine.

## Results

### Single-sample networks in spaceflight and ground control groups

**Fig. 1:**
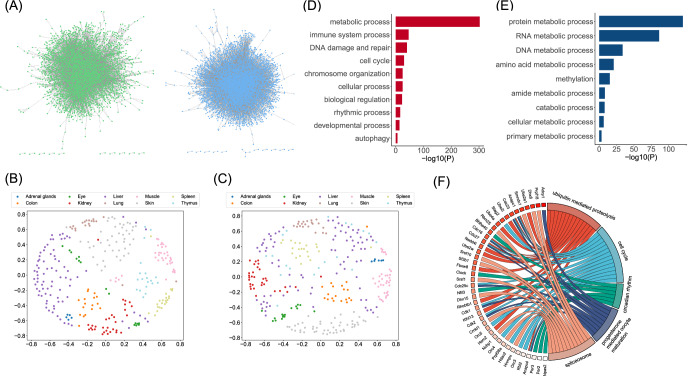
Single-sample networks and DIGs’ enrichment results. **A** Schematic diagram of a ground control SSN (green) and a spaceflight SSN (blue) from OSD-47. **B** A scatter plot of 290 ground control SSNs in two-dimensional space. **C** A scatter plot of 301 spaceflight SSNs in two-dimensional space. **D** The biological processes of 569 DIGs. Similar biological processes were integrated into a category by the GO tree, and the significance (negative logarithm of *P*-value) of all biological processes contained within a category was summed. **E** The metabolic processes of 569 DIGs. **F** The KEGG pathways of 569 DIGs. The lines between genes and pathways represent inclusion relationships. Genes are sorted by *P*-value, with lighter colors indicating smaller *P*-values.

The SSNs were constructed for 301 spaceflight samples and 290 ground control samples using LIONESS (see Supplementary Table 1 for degree vectors in 591 SSNs). To observe the structures of these SSNs, we conducted visualizations for two SSNs (a ground control sample and a spaceflight sample from OSD-47), as shown in Fig. 1A. The distance relationships among the 301 spaceflight SSNs and 290 ground control SSNs are illustrated in Fig. 1B, C respectively. Notably, SSNs from the same tissue tend to cluster together, indicating that the same tissues exhibit similar gene interaction networks. This result demonstrates that our SSNs can effectively reflect the gene interaction patterns in mice. Besides, the gene interaction patterns also exhibit tissue specificity, potentially differing among various tissues.

### Differentially interacted genes and their functions

Compared to the ground control group, there were 569 genes (DIGs) (Supplementary Table 2A) that exhibited significant changes (*P*-value < 0.05) in their interaction patterns in the spaceflight group. To explore the functions of these DIGs, we performed the GO enrichment analysis and categorized the biological processes (*P*-value < 0.05), as depicted in Fig. 1D and E. DIGs are mainly involved in metabolic process, immune system process, DNA damage and repair, cell cycle, chromosome organization, cellular process, biological regulation, rhythmic process, developmental process, and autophagy (Fig. 1D). Note that metabolic process includes protein metabolic process, RNA metabolic process, DNA metabolic process, and amino acid metabolic process, etc. (Fig. 1E). Moreover, KEGG-enrichment analysis shows that DIGs are mainly associated with five pathways, including ubiquitin-mediated proteolysis, cell cycle, circadian rhythm, progesterone-mediated oocyte maturation, and spliceosome (Fig. 1F).

### Contrasting patterns of gene interactions in low, medium, and high-dose groups

**Fig. 2:**
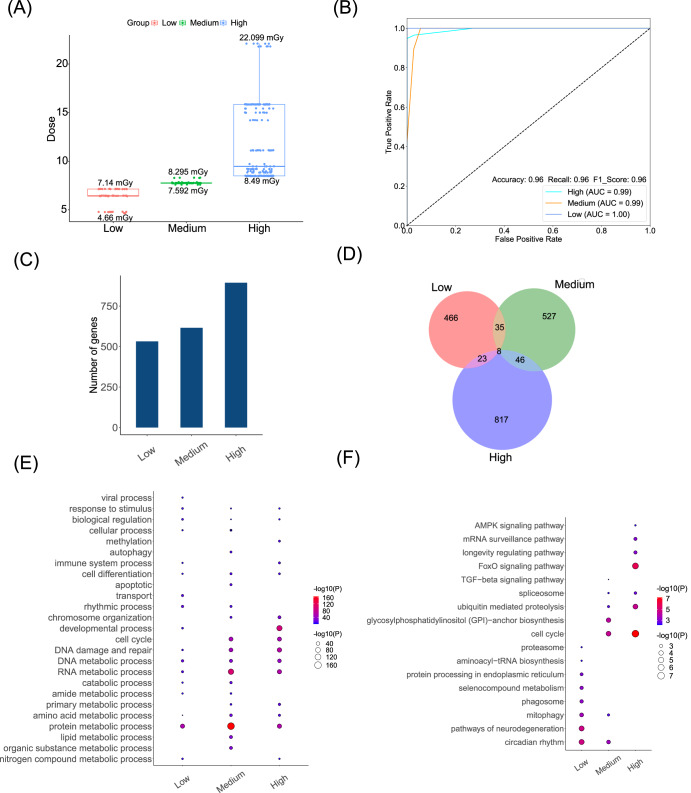
Three dose groups and their functions. **A** The doses of three groups (low, medium, and high). Each point denotes the absorbed dose of a sample. **B** The receiver operating characteristic (ROC) of dose grouping based on the KNN classifier. **C** The number of DIGs in three dose groups. **D** The Venn diagram of DIGs in three dose groups. **E** The biological processes in three dose groups. The size and color of the bubbles denote the negative logarithm of the *P*-value. **F** The KEGG pathways in three dose groups.

Based on the best classification results from KNN, we obtained the criteria for dividing dose groups: the low group is 4.66–7.14 mGy (53 samples), the medium group is 7.592–8.295 mGy (68 samples), and the high group is 8.49–22.099 mGy (180 samples) (Fig. 2A). The best KNN classifier for dose grouping achieved excellent performance, with an F1 Score of 0.94, and the AUC for the low, medium, and high classes were 0.99, 0.99, and 0.98, respectively (Fig. 2B), indicating a high similarity in gene interaction patterns within the dose groups.

We identified the DIGs for each dose group, respectively (namely, finding the ground control samples corresponding to spaceflight samples in each dose group, respectively, and conducting *T*-tests on the degree vectors of each gene) (Supplementary Table 2B). As the dose gradient increased, the number of DIGs in the three groups gradually increased (Fig. 2C). The DIGs in the three groups exhibit distinct differences, with only 8 DIGs being common to all groups, indicating a substantial dose effect on the gene interaction patterns (Fig. 2D).

The biological processes are more significant (smaller *P*-values) in the medium and high-dose groups (especially in the metabolic process, cell cycle, DNA damage, and repair). Notably, as the increase of radiation dose levels, the significance of the cell cycle, DNA damage and repair, and DNA metabolic process gradually increase (Fig. 2E). Among various metabolic processes, the protein metabolic process, DNA metabolic process, and RNA metabolic process exhibit high significance (Fig. 2E). According to the KEGG-enrichment results, medium and high doses will activate the cell cycle pathway, and the FoxO signaling pathway appears in the high-dose group. Moreover, circadian rhythm and rhythmic processes appeared in the low and medium-dose groups (Fig. 2F).

### Contrasting patterns of gene interactions in ten tissues

**Fig. 3:**
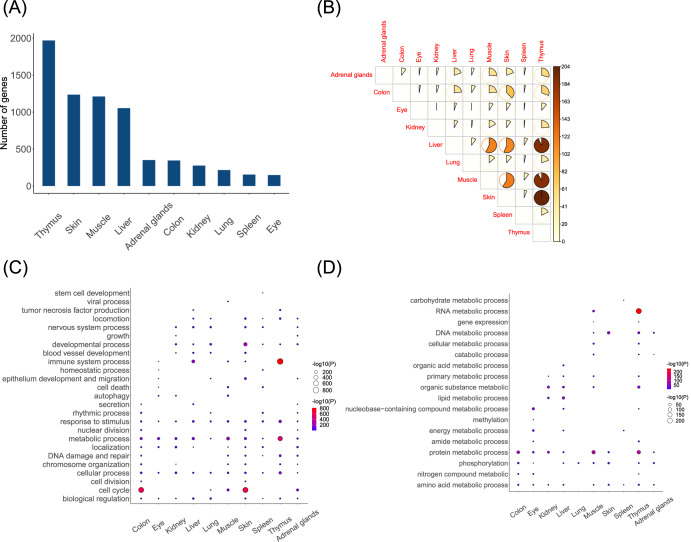
The DIGs and their functions in different tissues. **A** The number of DIGs in 10 tissues. **B** The intersection of DIGs in ten tissues. The size and color of the fans represent the number of overlapping DIGs in the two tissues. **C** The biological processes in 10 tissues. **D** The metabolic processes in 10 tissues.

For ten tissues, the DIGs were also identified (between spaceflights and ground controls) separately (Fig. 3A and Supplementary Table 2C). The thymus has the highest number of DIGs (1968), while the eye has the fewest (148). Tissues with more than 1000 DIGs also include skin, muscle, and liver. While most of the DIGs between tissues are specific, there are still some tissues that share common DIGs, such as thymus with skin, liver, and muscle; skin with liver and muscle; liver with muscle (Fig. 3B).

Observing the biological processes of DIGs in different tissues, it can be seen that all 10 tissues contain metabolic processes, responses to stimulus, and cellular processes. The cell cycle in the colon and skin, immune system process, and metabolic process in the thymus have high significance (Fig. 3C). Further analysis shows that protein metabolism and nucleic acid metabolism are more prominent in the metabolic process, and amino acid metabolism appears in almost all tissues (except for lung) (Fig. 3D).

### Radiation responsiveness of different tissues

**Fig. 4:**
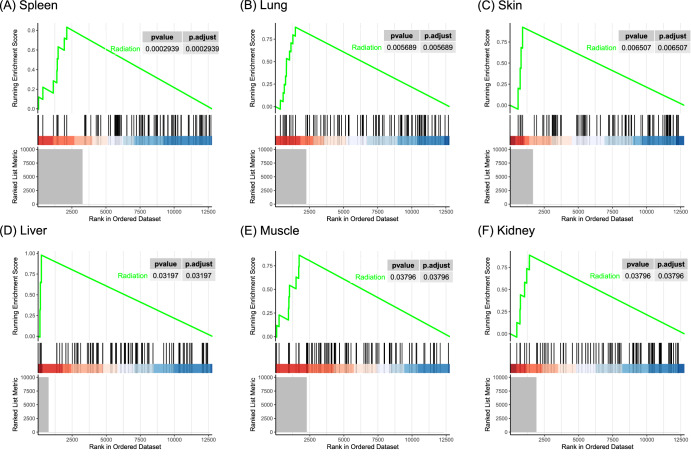
GSEA plots of radiation gene set in six tissues. **A** GSEA plots of radiation gene set in spleen. **B** GSEA plots of radiation gene set in lung. **C** GSEA plots of radiation gene set in skin. **D** GSEA plots of radiation gene set in liver. **E** GSEA plots of radiation gene set in muscle. **F** GSEA plots of radiation gene set in kidney.

According to the GSEA, we found that there were gene rankings in six tissues were significantly associated with the radiation gene set: spleen (*P*-value = 2.94E−04), lung (*P*-value = 5.69E−03), skin (*P*-value = 6.51E−03), liver (*P*-value = 3.20E−02), muscle (*P*-value = 3.80E−02), and kidney (*P*-value = 3.80E−02) (Fig. 4), indicating that above tissues exhibit higher radiation responsiveness.

### The diseases and virus-related pathways induced by spaceflight

**Fig. 5:**
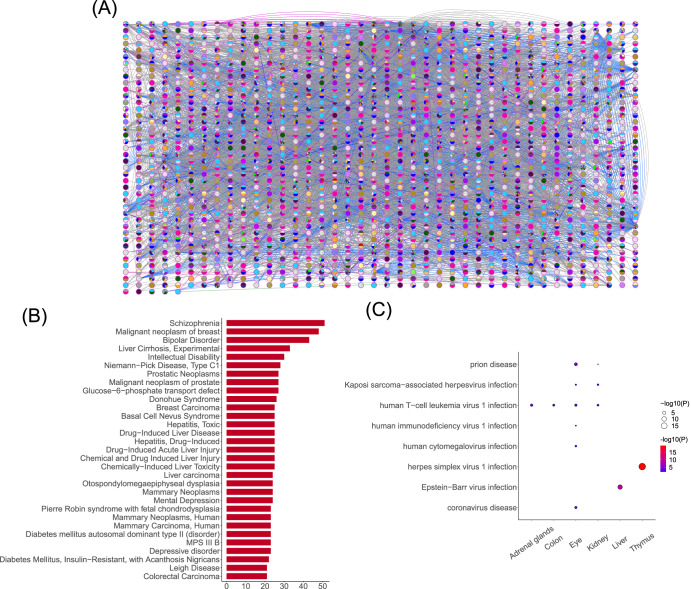
The diseases and virus-related pathways induced by spaceflight. **A** A human gene–disease network. Nodes denote genes or diseases, edges denote the relationships between diseases and genes, and colors denote the types of diseases. The correspondence between colors and disease types can be found in Supplementary Fig. 1. **B** The degrees of human diseases. **C** Virus-related pathways or diseases in some tissues.

We constructed a human gene–disease network using DisGeNET (Fig. 5A) and calculated the degrees of all diseases in this network. Figure 5B displays diseases with a degree > 20. Spaceflight can induce mental disorders, cancer, depression, liver injury, inherited metabolic disorders, genetic disease, dysplasia, mitochondrial disease, etc.

To validate the above human diseases, we obtained a human dataset (OSD-546) under spaceflight conditions from GeneLab. Note that detailed information about OSD-546 can be obtained from GeneLab (https://osdr.nasa.gov/bio/repo/data/studies/OSD-546). We employed the same method to construct SSNs for each sample of this dataset. Then the DIGs were identified, and the corresponding diseases were predicted (Supplementary Fig. 2). In OSD-546, we also found mental disorders (schizophrenia), cancers (malignant neoplasm of breast, malignant neoplasm of prostate, colorectal carcinoma, liver carcinoma, colorectal neoplasms, etc.), depression (depressive disorder, mental depression, and bipolar disorder), genetic diseases (Alzheimer disease, familial, type 3, amyotrophic lateral sclerosis, ataxia telangiectasia, and cardio-facio-cutaneous syndrome), and liver cirrhosis. The above diseases are consistent with those found in the mouse datasets, demonstrating the reliability of the results in this study.

Furthermore, KEGG-enrichment indicates that some virus-related pathways or diseases appear in various tissues, including human T-cell leukemia virus 1 infection, human cytomegalovirus infection, Kaposi sarcoma-associated herpesvirus infection, human immunodeficiency virus 1 infection, Epstein-Barr virus infection, herpes simplex virus 1 infection, prion disease, coronavirus disease, hepatitis B, hepatitis C, influenza A, where herpes simplex virus 1 infection and Epstein-Barr virus infection are the most significant (smaller *P*-values) (Fig. 5C).

### Differentially interacted network and hub genes

**Fig. 6:**
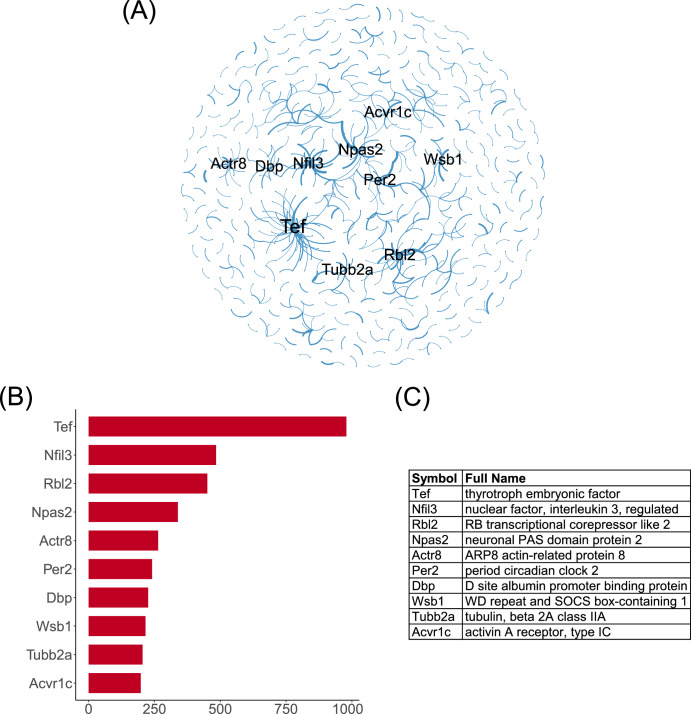
Differentially interacted network. **A** A DIN between the ground control and spaceflight groups. The thickness of the edges denotes differential counts. Hub genes are prominently highlighted. **B** The weighted degrees of hub genes in the DIN. **C** The full names of hub genes.

We extracted edges with differential counts of more than 20 between the ground control and spaceflight groups to form a DIN (Fig. 6A and Supplementary Table 3). Where the top 9 edges with the highest differential counts are as follows: (Hnf4a, Npas2), (Cdk16, Nfil3), (Rbl2, Cdk1), (Npas2, Vdr), (Arntl, Usp2), (Nfil3, Zfp521), (Usp2, Per2), (Wsb1, Hsp90aa1), (Psmd12, Htra3). Next, we calculated the weighted-degree of each gene in the DIN and identified 10 hub genes: Tef, Nfil3, Rbl2, Npas2, Actr8, Per2, Dbp, Wsb1, Tubb2a, Acvr1c (Fig. 6B, C).

**Fig. 7:**
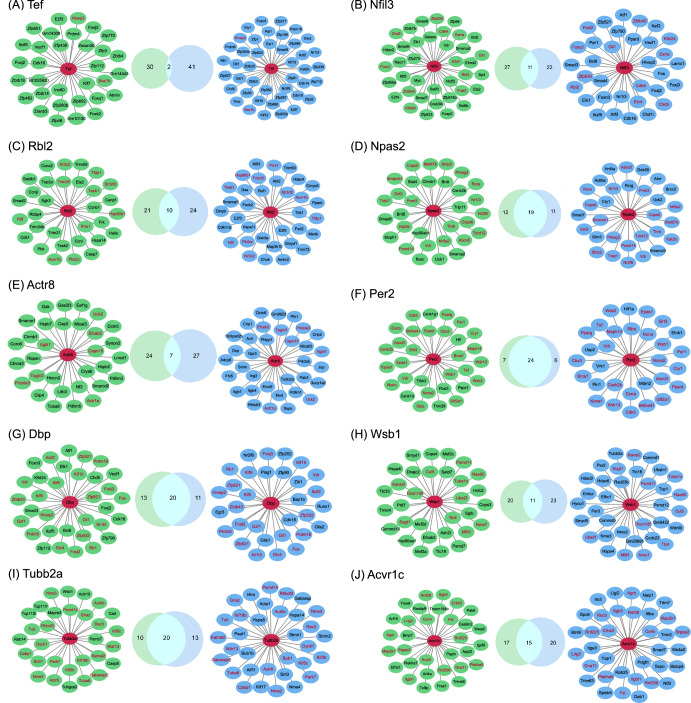
GC-NET and SF-NET activated by genes in the ground control and spaceflight groups. The green networks represent the GC-NETs, the blue networks represent the SF-NETs, and the red nodes indicate hub genes. The Venn diagrams represent the overlap of genes between GC-NETs and SF-NETs, and the overlapping genes are annotated with red font.

To further analyze the central role of each hub gene in the interaction network, we extracted the top 30 genes most connected to each hub gene in the ground control and spaceflight groups, respectively (all genes with the same number of connections were included). Therefore, there are two networks centered around each hub gene, referred to as GC-NET (a network in ground control group) and SF-NET (a network in spaceflight group) (Fig. 7), which are primarily involved in circadian rhythms, DNA damage and repair, cell cycle, metabolic process (mainly protein/amino acid, nucleic acid, and energy metabolism), immune process, development and differentiation, nervous system processes, methylation, etc. (Table 1).

**Table 1:** The biological processes in GC-NETs and SF-NETs activated by 10 hub genes

Gene	GC-NET	SF-NET
Tef	DNA damage and repair, differentiation	Circadian rhythm, cell cycle, development, RNA metabolic process, apoptosis, chromosome organization
Nfil3	Development and differentiation, RNA metabolic process, cell cycle, response to stimulus, immune process	Development and differentiation, RNA metabolic process, cell cycle, response to stimulus, chromosome organization
Rbl2	Cell cycle, virus process, protein metabolic process, DNA metabolic process, amino acid metabolic process, immune process, phosphorylation, development, response to stimulus	Cell cycle, viral process, protein metabolic process, DNA metabolic process, immune process, phosphorylation, differentiation, response to stimulus, apoptosis
Npas2	Circadian rhythm, DNA damage and repair, cell cycle, metabolic process, immune process, development and differentiation, response to stimulus	Circadian rhythm, DNA damage and repair, cell cycle, metabolic process, immune process, development and differentiation, homeostatic process
Actr8	DNA damage and repair, muscle process, development	DNA damage and repair, muscle process, development, metabolic process, locomotion, response to stimulus, signaling pathway
Per2	Circadian rhythm, DNA damage and repair, cell cycle, response to stimulus, metabolic process, development, phosphorylation, homeostatic process, oxidative stress, immune process	Circadian rhythm, DNA damage and repair, cell cycle, response to stimulus, metabolic process, development and differentiation, phosphorylation, apoptosis, homeostatic process, oxidative stress
Dbp	Metabolic process, development and differentiation, immune process, homeostatic process	Metabolic processes, development and differentiation, immune process
Wsb1	Methylation, protein metabolic process, development and differentiation, chromosome organization	Energy metabolic process, protein metabolic process, nucleic acid metabolic process, development, chromosome organization, homeostatic process
Tubb2a	Energy metabolic process, nucleic acid metabolic process, cell cycle, apoptosis, chromosome organization	Nervous system process, energy metabolic process, nucleic acid metabolic process, protein metabolic process, cell cycle, chromosome organization, autophagy
Acvr1c	Nervous system processes, phosphorylation, development and differentiation, immune process, response to stimulus	Nervous system process, phosphorylation, development and differentiation, immune process, response to stimulus, apoptosis

The hub genes activate distinct gene interaction networks between the ground control group and the spaceflight group. For example, there are only two common genes in the two networks activated by Tef. However, functional analysis reveals that although GC-NET and SF-NET are not entirely identical, they perform similar functions. For instance, both GC-NET and SF-NET for Tef and Nfil3 are involved in development and differentiation, and the two networks for Rbl2 are associated with the cell cycle, viral process, and metabolic process. Similarly, networks activated by genes like Npas2, Actr8, Per2, Dbp, and Acvr1 also exhibit similar functions. Therefore, spaceflight leads to changes in the topological structure of these functional networks.

## Discussion

The space environment, where microgravity and radiation continuously persist, induces complex bio-effects. However, due to differences in exposure duration and flight orbits, the impact of radiation and microgravity on organisms varies across different missions, which makes it challenging to gain an overall understanding of the bio-effects induced by spaceflight. To address this challenge, we integrated datasets from multiple spaceflight missions (with different exposure duration, orbits, and absorbed doses) and conducted a comprehensive analysis of the gene interaction patterns induced by spaceflight. Our approach involved a molecular network perspective to elucidate the bio-effects of spaceflight and predict potential health risks. In a gene interaction network, each node represents a gene, while each edge denotes a gene-to-gene interaction. Herein, we analyzed changes in the topological structure of the gene interaction network from both node and edge perspectives, which allows for a more comprehensive understanding of the changing characteristics in gene interaction patterns induced by spaceflight and provides a more refined depiction of network structures.

To explore the contrasting patterns of gene interactions between spaceflights and ground controls, we developed a bioinformatics pipeline based on SSNs and analyzed the mouse transcriptome profiles from multiple datasets in the GeneLab platform. Due to the fact that the mouse samples are derived from various flight missions and involve differences in sequencing technologies and experimental platforms, the transcriptome profiles exhibit inherent heterogeneity, making direct comparisons unfeasible. Herein, we tackled this issue through a two-step approach: Initially, we applied the *rlogTransformation()* in the DESeq2 R package to mitigate batch effects among the samples. Subsequently, the SSN method was employed to convert expression levels into gene interactions within individuals, ensuring comparability across different samples. Above all, *rlogTransformation()* transforms the count data to the log2 scale in a way that minimizes differences between samples with small counts and which normalizes with respect to library size. Therefore, *the rlogTransformation()* can transform data to a normal distribution in order to reduce the impact of batch effects and heteroscedasticity between samples^19^. In addition, the authors of LIONESS have experimentally demonstrated that this method is insensitive to the choice of background samples, meaning that SSNs constructed by the same sample in different background datasets are similar, and LIONESS performs robustly even when different subtypes are present in the background samples^11^. Thus, even if the gene expression data after normalization is still heterogeneous, LIONESS can construct an SSN with good performance, which ensures the effectiveness of data integration and the reliability of the SSN analysis in this paper. The scatter plot obtained from MDS reveals that SSNs from the same tissue tend to cluster together, demonstrating that our SSNs can effectively capture the gene interaction patterns.

According to the enrichment results, spaceflight has induced substantial metabolic disruptions, particularly in biological processes related to protein/amino acid metabolism and nucleic acid (DNA/RNA) metabolism (Figs. 1E, 2E, and 3D). Previous studies have indicated that spaceflight inhibits the protein metabolism network in mice^20^. Mao et al. found substantial changes in biochemicals associated with amino acid and carbohydrate metabolism in mice that underwent spaceflight and proposed that these changes might be the consequence of increased regulation in cellular antioxidants, ROS production, and tissue remodeling^21^. Besides, the metabolism induced by DNA damage can be quite complex, and a number of DNA metabolic proteins and pathways are involved^22^. Thus, some of the alterations in the nucleic acid metabolic process may be associated with the severe DNA damage induced by spaceflight. The above research also highlighted the dysregulation of protein/amino acid and nucleic acid metabolism during spaceflight, while we revealed the gene interaction mechanisms driving these changes.

To observe the dose-dependent effects in gene interaction patterns, KNN was employed for dose grouping in this study. We strictly adhered to a five-fold cross-validation and prudently reported our results. Nevertheless, since the new test samples need to be transformed into SSNs for dose prediction, their data distribution may affect the model’s performance. Therefore, we assumed that the new test samples should be in a similar distribution as required by LIONESS. We discovered a dose-dependent effect in gene interaction patterns. With increasing doses, the number of DIGs gradually increased, and the significance of processes related to genetic material damage (such as cell cycle, DNA damage repair, and DNA metabolism) also increased progressively. Therefore, higher levels of space radiation could lead to more pronounced disruptions in gene interaction networks and result in stronger damage effects on genetic material. There have been some reports on the bio-effects induced by radiation doses. Jain et al. found that in chronic low-dose radiation exposure, the number of differentially expressed genes (DEGs) increased with a dose-dependent, and these genes were primarily associated with DNA damage response signaling, DNA repair, and cell cycle arrest^23^. It can be inferred that in a low-dose space radiation environment, both the number of differential genes and the magnitude of damage effects may escalate with increasing doses, which is consistent with our findings. We also discovered that mice exposed to radiation doses within the same range (4.66–7.14, 7.592–8.295, 8.49–22.099 mGy) exhibited similar gene interaction patterns (with AUC values of 0.99, 0.99, and 0.98, respectively). Notably, dose grouping may be influenced by the mouse strains. Previous studies indicated that C57 and BALB/c mice exhibited higher radiation sensitivity^24,25^. In our results, the high-dose group contains a larger number of samples with a wider range of doses. C57BL/6NTac mice with 8.49 mGy and BALB/c mice with 8.97 mGy were both categorized in the high-dose group, possibly due to their higher radiation sensitivity.

The gene interaction patterns induced by spaceflight exhibit specificity in different tissues. According to the GSEA, we found a strong correlation between gene rankings in the spleen, lung, skin and the radiation gene set, suggesting that these three tissues were most strongly influenced by space radiation. Based on relevant studies, the skin absorbs the highest radiation doses and dose equivalents within spacecraft^26^, while the lung demonstrates the highest nominal cancer risks induced by radiation^27^. Hence, it can be inferred that the radiation responsiveness of a tissue may be related to both the absorbed dose and its own anti-stress mechanism. Based on our results, each tissue activates a specific gene interaction network to respond to space stressors, and most of these networks participate in the metabolic process, response to stimulus, cellular process, localization, DNA damage and repair, cell cycle, etc. As aforementioned, while the biological functions involved in each tissue exhibit similarities, their stress-response mechanisms and pathways are different (most of the DIGs between tissues are specific).

The KEGG-enrichment results reveal that spaceflight induces several pathways or diseases associated with viruses. Many studies have already reported viral reactivation during spaceflight: Astronauts shed Epstein-Barr virus (EBV), varicella-zoster virus (VZV), and herpes-simplex-1 (HSV-1) in saliva and cytomegalovirus (CMV) in urine. Larger quantities and increased frequencies for these viruses are found during spaceflight as compared to before or after flight samples and their matched healthy controls^28^. HSV-1 establishes latency in various cranial nerve ganglia and often reactivates in response to stress-associated immune system dysregulation^29^. EBV reactivates during spaceflight, with EBV shedding in saliva increasing to levels 10 times those observed pre- and post-flight. During spaceflight, EBV infection leads to an increase in cell DNA damage, and cells infected with EBV are less prone to apoptosis^30^. The reactivation of latent viruses during spaceflight is commonly considered a consequence of immune system dysregulation^31^, and the virus-specific T-cell function is depressed both during and following spaceflight^32^. Our analysis of the gene interaction networks further confirms the phenomenon of viral reactivation during spaceflight. What was more, we revealed that mice would activate specific gene interaction networks to respond to the viral reactivation during spaceflight. Moreover, we identified new viruses (including prion, Hepatitis B virus, Hepatitis C virus, and influenza virus), and future studies could explore the reactivation of these viruses during spaceflight in greater detail.

The cellular and molecular responses of spaceflight have substantial physiological and systemic implications regarding astronaut health, such as cardiovascular dysregulation, CNS impairments, increased cancer risk, bone loss, increased liver disease, circadian rhythm dysregulation, etc.^1^ Over the past five decades, psychiatric issues have been documented in orbital spaceflight^33^. The extreme environments that astronauts face during spaceflight can easily lead to symptoms such as sleep disturbance, fatigue, and irritability, which may cause depression-like behavior and have a serious impact on the normal execution of space missions^34,35^. According to DisGeNET analysis, numerous DIGs are associated with depression and mental disorders, implying the possibility of using targeted drugs to alleviate mental and psychological issues among astronauts. While many of these systemic and physiological health risks of spaceflight have been well documented, much is left to be discovered^36^. Therefore, establishing multi-omics approaches to further investigate these health risks is of paramount importance and will advance the development of personalized aerospace medicine^36^. In this work, we have predicted most of the known diseases based on transcriptome and protein interactome, indicating that disturbance in gene interaction patterns can reflect the occurrence and development of diseases induced by spaceflight.

Remarkably, through the comparison of gene interaction patterns under space and ground conditions, we have also identified some new diseases, such as inherited metabolic disorders (IMD) (Niemann-Pick disease, glucose-6-phosphate transport defect, Donohue syndrome, Mucopolysaccharidosis III B) and mitochondrial diseases (Leigh disease). Most IMDs are caused by the absence or deficiency of a specific enzyme that catalyzes a step in the biochemical pathway. As metabolism is controlled by the input of genes and the environment, metabolic disorders result from some disturbance in the interaction between genes and environmental factors^37^. Our findings suggest that space stressors may lead to changes in genes involved in metabolic pathways, thereby triggering severe metabolic diseases. Our results are of great significance for the prevention and precise treatment of potential diseases in astronauts, and assessment of spaceflight risks.

We identified 10 new hub genes that played important roles in spaceflight based on DIN. TEF is an element of the “cellular clock”, which plays a key role in circadian rhythm^38^. Moreover, upregulation of TEF expression substantially retards cancer cell growth by inhibiting the G1/S transition via regulating AKT/FOXOs signaling^39^. NFIL3 is also a rhythm gene, which is involved in energy metabolism and immune cell differentiation, and its abnormal expression is related to metabolic diseases, inflammation, and tumors^40^. RBL2 participates in multiple important pathways of the cell cycle^41,42^. NPAS2 is one of the core genes that control the rhythm of the biological clock^43^, which is associated with anxiety and cancer^44,45^. PER2 is a core circadian clock protein^46^, which plays an active role in low-level radiation adaptive radioprotection^47^. Dbp is also related to circadian rhythm, which governs the circadian transcription of a number of hepatic detoxification and metabolic enzymes prior to the activity phase and subsequent food intake of mice^48^. WSB-1 is involved in DNA damage response by targeting homeodomain-interacting protein kinase 2 (HIPK2) for ubiquitination and degradation^49^. TUBB2A is a gene related to structural abnormalities in the brain^50^, which may play a role in neurologic damage under spaceflight. ACVR1C is associated with skin diseases, and studies have shown that it may be a target for preventing or treating UV-induced disruptions in lipid metabolism and associated skin disorders^51^. Therefore, many hub genes are related to circadian rhythms, while other hub genes are associated with the cell cycle, DNA damage response, nervous system disorders, and skin injuries. Further analysis reveals that hub genes activate distinct gene interaction networks between the ground control and spaceflight group, regulating processes such as circadian rhythms, DNA damage and repair, cell cycle, metabolic process (mainly protein/amino acid, nucleic acid, and energy metabolism), immune process, development and differentiation, nervous system processes, methylation, etc. Note that most of the hub genes’ functions are contained within the networks they activate, providing evidence that genes function through networks. These hub genes may play crucial roles in the organism’s response to spaceflight and can serve as potential targets for risk mitigation.

Many studies reported circadian rhythm disruption during spaceflight, which was visible in many physiological aspects, for example, shorter sleep durations^52^, musculoskeletal atrophy^53^, physiological aspects^54^, etc. Da Silveira et al. also discovered substantial enrichment of circadian rhythm through the multi-omics profiles^9^. We have identified that genes related to circadian rhythms play a central role in the DIN during spaceflight. Therefore, there is an urgent need to establish preventive measures for astronauts using circadian biology to minimize the effect of space missions on their health and performance^55^.

In summary, by developing a bioinformatics pipeline based on SSNs, this study provides a comprehensive insight into gene interaction patterns within mouse tissues under spaceflight conditions. Spaceflight disrupts the gene interaction patterns in mice, with this alteration exhibiting a radiation dose-dependent effect. Different tissues exhibit varying gene interaction patterns in response to spaceflight, with the spleen, lung, and skin being the most responsive to space radiation. Ten hub genes that played key roles in DIN were identified, which activated gene networks involved in circadian rhythms, DNA damage and repair, cell cycle, metabolism, etc., under spaceflight.

## Methods

### Collection and preprocessing of transcriptome profiles

The transcriptome datasets of mice utilized in this study were obtained from NASA’s GeneLab platform (genelab.nasa.gov). We collected all existing datasets including spaceflight samples and providing raw counts of RNA-Seq from GeneLab (accessed on July 1, 2023). There were 30 datasets encompassing 10 tissues (adrenal glands, colon, eye, kidney, liver, lung, muscle, skin, spleen, thymus) from RNA-sequencing, including 301 samples from spaceflight conditions and 290 samples from ground control conditions (Supplementary Table 4). We removed genes with zero expression levels in over 20% of the samples. Then the *rlogTransformation()*^19^ in the *DESeq2* R package was used to transform the count data to log2 scale (data normalization).

### Construction of single-sample networks

The SSNs were constructed for 591 mouse samples using LIONESS, respectively. LIONESS constructs the state transition network by calculating the edge statistical significance between all samples and the samples without a given single sample. The network specific to a sample *s* was calculated according to the following equation:

1 $${e}_{{ij}}^{{s}}=N({e}_{{ij}}^{{N}}-{e}_{{ij}}^{{N-s}})+{e}_{{ij}}^{{N-s}}$$

where ${e}_{{ij}}^{{s}}$ is the correlation between gene *i* and *j* in sample *s*, ${e}_{{ij}}^{{N}}$ is the Pearson correlation coefficient (PCC) of gene *i* and *j* in *N* samples, ${e}_{{ij}}^{N-s}$ is the PCC of gene *i* and *j* after removing sample *s*, and *N* is the total number of samples.

After obtaining the distribution *D* of the ${e}_{{ij}}^{s}$ (absolute values) of all gene pairs, it is necessary to choose a threshold to determine the edges in an SSN. Following ref. ^56^, we set the threshold as

2 $$w=\mu (D)+2\delta (D)$$

where $\mu (D)$ and $\delta (D)$ are the mean and standard deviation of the *D* in sample *s*. Namely, in the sample *s*, if the ${\rm{|}}{e}_{{ij}}^{s}|$ of two genes is greater than *w*, then there is an edge between them in the SSN.

### Characterization of gene interaction patterns

To obtain the gene interaction patterns for each sample, we intersected the SSNs constructed by LIONESS (transcriptome) with the PPI network (protein interactome) (i.e., only retaining the nodes and edges that were common to both the SSN and PPI network), resulting in a final SSN used for downstream analysis. The PPI network was obtained from the STRING (string-db.org), and only experimentally confirmed gene interactions were retained, which not only ensured the reliability of interactions in the SSNs but also enabled SSNs to characterize individual-specific gene interaction patterns.

To observe the distribution of SSNs, the distances between all SSNs in the spaceflight group were calculated. For two SSNs, ${G}_{1}({V}_{1},{E}_{1})$ and ${G}_{2}({V}_{2},{E}_{2})$, where *V* was the set of nodes and *E* was the set of edges, the distance between them was calculated using Eq. (3):

3 $${{dist}}=1-\frac{{E}_{1}\cap {E}_{2}}{{E}_{1}\cup {E}_{2}}$$

where the latter part of Eq. (3) denotes the Jaccard similarity coefficient.

After obtaining the distance matrix between all SSNs, multidimensional scaling (MDS) was employed to reduce the SSNs to a two-dimensional space, and a scatter plot was generated using the *matplotlib* Python package. MDS accomplishes dimensionality reduction by minimizing the error between distances in the original data (SSNs) and distances in a two-dimensional space, which faithfully preserves the relative distances between SSNs in the lower-dimensional space^57^. In this study, the *MDS()* in *sklearn* python package was employed to implement MDS.

### Extraction of node features in SSN and identification of differentially interacted genes

Herein, the degrees were represented as node features. Given a node *v*, where $N(v)$ denoted the collection of its neighbors in an SSN, the degree of *v* was defined as follows:

4 $${{{Degree}}}(v)={\rm{|}}N(v){\rm{|}}$$

where |∙| denotes the number of elements in the set.

Next, we took the union of genes present in all SSNs and constructed a degree vector **d** for each gene (Eq. (5)). Here, *n* denotes the number of samples (which is 591 in our work). For any given gene, *d*_*i*_ denotes the degree of this gene in the *i*th SSN (if this gene is not present in the *i*th SSN, then *d*_*i*_ = 0).

5 $${\bf{d}}=({d}_{1},{d}_{2},\cdots ,{d}_{n})$$

**Fig. 8:**
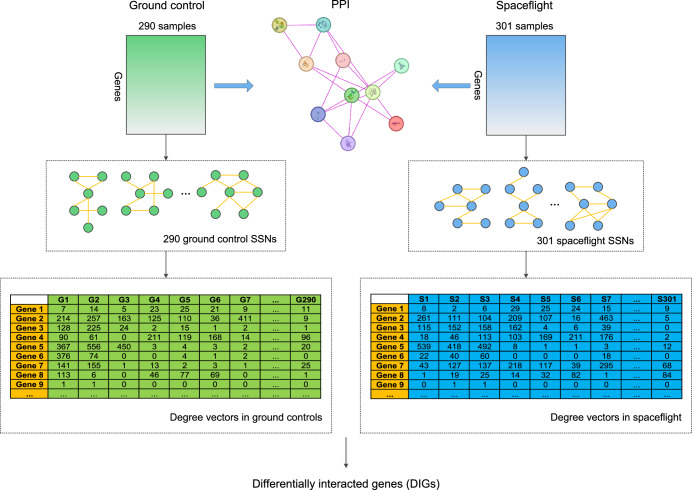
Schematic representation of the analysis workflow to identify DIGs. 591 spaceflight mouse samples (301 spaceflight and 290 ground control samples) from 30 datasets were integrated, and a SSN was constructed for each individual. To obtain the gene interaction patterns for each sample, the SSNs that constructed by LIONESS were intersected with the PPI network. The degrees were represented as node features. A two-sided T-test was conducted on the degree vectors of a gene in the ground control and spaceflight groups, and the genes with P-values < 0.05 were defined as DIGs.

Finally, we conducted a two-sided *T*-test on the ${{\bf{d}}}_{{{\rm {GC}}}}=({d}_{1},{d}_{2},\cdots ,{d}_{290})$ (degree vector of a gene in the ground control group) and ${{\bf{d}}}_{{{\rm {SF}}}}=({d}_{291},{d}_{2},\cdots ,{d}_{591})$ (degree vector of a gene in the spaceflight group), defining genes with a *P*-value < 0.05 as differentially interacted genes (DIGs). Specifically, homogeneity of variances was tested using *levene()* in *scipy* python package, and then the *T*-test was performed using *ttest_ind()* in *scipy* python package. The workflow of identifying DIGs is shown in Fig. 8.

### Dose grouping based on K-nearest neighbors classifiers

The radiation dose absorbed by each sample was obtained from the “Environmental Data” of the GeneLab platform (genelab.nasa.gov/environmental/radiation) (Supplementary Table 4). To explore the effect of different space radiation doses on gene interaction patterns, we used machine learning to divide all the samples into three dose groups, including low (L), medium (M), and high (H), respectively. To obtain the optimal radiation dose groups, we exhausted all partitioning schemes, i.e., different cutoff thresholds were used to divide all doses into three groups while ensuring that $h \,>\, m \,>\, l,\forall \,h\in H,m\in M,l\in L$. For every partitioning scheme, the same classifier (K-Nearest Neighbors, KNN) was employed for classification, and the best choice was the partitioning scheme that yielded the highest F1 score. The KNN classifier was implemented using *KNeighborsClassifier()* in *sklearn* python package. The input features for the classifier were the degree vectors of all SSNs, while the classification labels were the dose groups to which these SSNs belong. Of note, the experiment employed a five-fold cross-validation method. We believed that this classifier-based grouping carried greater biological significance.

### Prediction of spaceflight-induced diseases

DisGeNET (disgenet.org) was used to predict human diseases that spaceflight might induce, and the specific steps were as follows: Initially, we downloaded the mapping file for mouse-human homologous genes from the Ensembl database (asia.ensembl.org) and mapped DIGs to human genes (human-DIGs). Subsequently, the human DIGs were inputted into the DisGeNET cytoscape app^58^ to construct a gene–disease network. Where the parameters “Score” were set from 0.3 to 1, and “EI” was set from 0 to 1. Lastly, we computed the degree of each disease in the gene–disease network, where a higher degree indicated a potentially stronger association with spaceflight.

### Identification of hub genes in contrasting patterns of gene interactions

We counted the occurrences of each edge in 301 spaceflight SSNs and 290 ground control SSNs, respectively. Next, the edges with differential counts of more than 20 between the two groups were extracted to form a differentially interacted network (DIN). In the DIN, the edge weights represented the difference in occurrence counts. For instance, if an edge appeared *n*_1_ times in the ground control group and *n*_2_ times in the spaceflight group, its weight would be ${\rm{|}}{n}_{1}-{n}_{2}{\rm{|}}$. We summed the weights of edges connected to each gene in the DIN (referred to as weighted-degree of a gene) and defined the top 10 genes with the highest weighted-degree as hub genes.

### Enrichment analysis

GO and KEGG-enrichment analyses were performed on the DIGs using Metascape (metascape.org)^59^, and the *P*-value < 0.05 was considered statistically significant. Furthermore, to investigate the radiation responsiveness of different tissues, we curated a radiation gene set and identified which tissue’s gene list was associated with this gene set (P-value < 0.05) using GSEA^60^. To note, GSEA was implemented using *GSEA()* in *clusterProfiler* R package and plotted using *gseaplot2()* in *enrichplot* R package. The gene list was sorted in descending order according to Fold Change (FC), and the FC of the *i*th gene could be calculated according to Eq. (6).

6 $${{{{FC}}}}_{i}=\frac{{\rm{mean}}({{\bf{d}}}_{i}^{{\rm{SF}}})}{{\rm{mean}}({{\bf{d}}}_{i}^{{\rm{GC}}})}$$

Here, ${\rm{mean}}({{\boldsymbol{d}}}_{i}^{{\rm{SF}}})$ and ${\rm{mean}}({{\boldsymbol{d}}}_{i}^{{\rm{GC}}})$ represent the mean degree of gene *i* in the spaceflight group and ground control group of a specific tissue, respectively. The method for curating the radiation gene set was as follows: firstly, we identified four GO terms related to space radiation: GO:0009314 (Response to radiation), GO:0010212 (Response to ionizing radiation), GO:0071478 (Cellular response to radiation), GO:0071479 (Cellular response to ionizing radiation). Next, we obtained genes annotated to these GO terms from the STRING and combined these genes to create a radiation gene set (Supplementary Table 5).

### Reporting summary

Further information on research design is available in the Nature Research Reporting Summary linked to this article.

### Supplementary information

Supplementary Information

Supplementary Table 1

Reporting Summary

## Data Availability

Source data for this study are publicly available in the GeneLab data repository (genelab.nasa.gov) under the Accession codes OSD-47, OSD-98, OSD-99, OSD-100, OSD-101, OSD-102, OSD-103, OSD-104, OSD-105, OSD-137, OSD-162, OSD-163, OSD-164, OSD-168, OSD-173, OSD-194, OSD-238, OSD-240, OSD-241, OSD-242, OSD-243, OSD-244, OSD-245, OSD-246, OSD-247, OSD-248, OSD-253, OSD-288, OSD-379, OSD-401. All relevant data are available from the authors.

## Code availability

All programs, packages, and functions used in this manuscript are freely available and are referred to in the “Methods” section.
