# Supplementary material for: Using single-sample networks to identify the contrasting patterns of gene interactions and reveal the radiation dose-dependent effects in multiple tissues of spaceflight mice
Source: NPJ Microgravity. 2024 Apr 4;10:45. doi: 10.1038/s41526-024-00383-7 (PMC10995210; doi:10.1038/s41526-024-00383-7)
Supplement: Supplementary file 1 — Supplementary Information [file 41526_2024_383_MOESM1_ESM.pdf]

Using single-sample networks to identify the contrasting patterns of gene interactions and reveal the radiation dose-independent effects in multiple tissues of spaceflight mice

Yan Zhang[1], Lei Zhao[1,*], Yeqing Sun[1,*]

[1] *Institute of Environmental Systems Biology, College of Environmental Science and Engineering, Dalian Maritime University, Dalian 116026, Liaoning, China*

* Corresponding author:

Lei Zhao

Linghai Road, Dalian 116026, Liaoning, China

Email address: zhaol@dlmu.edu.cn

Yeqing Sun

Linghai Road, Dalian 116026, Liaoning, China

Email address: yqsun@dlmu.edu.cn

Supplementary Figures

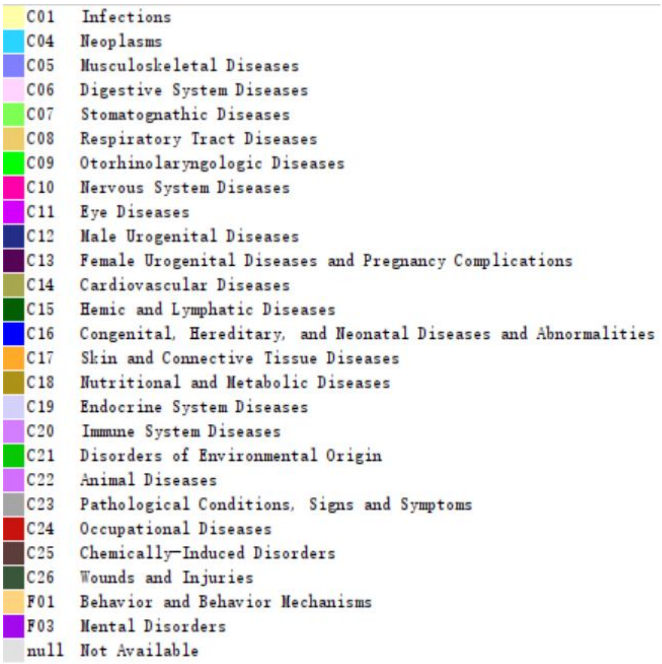

Supplementary Figure 1. The correspondence between colors and disease types in Fig. 5A.

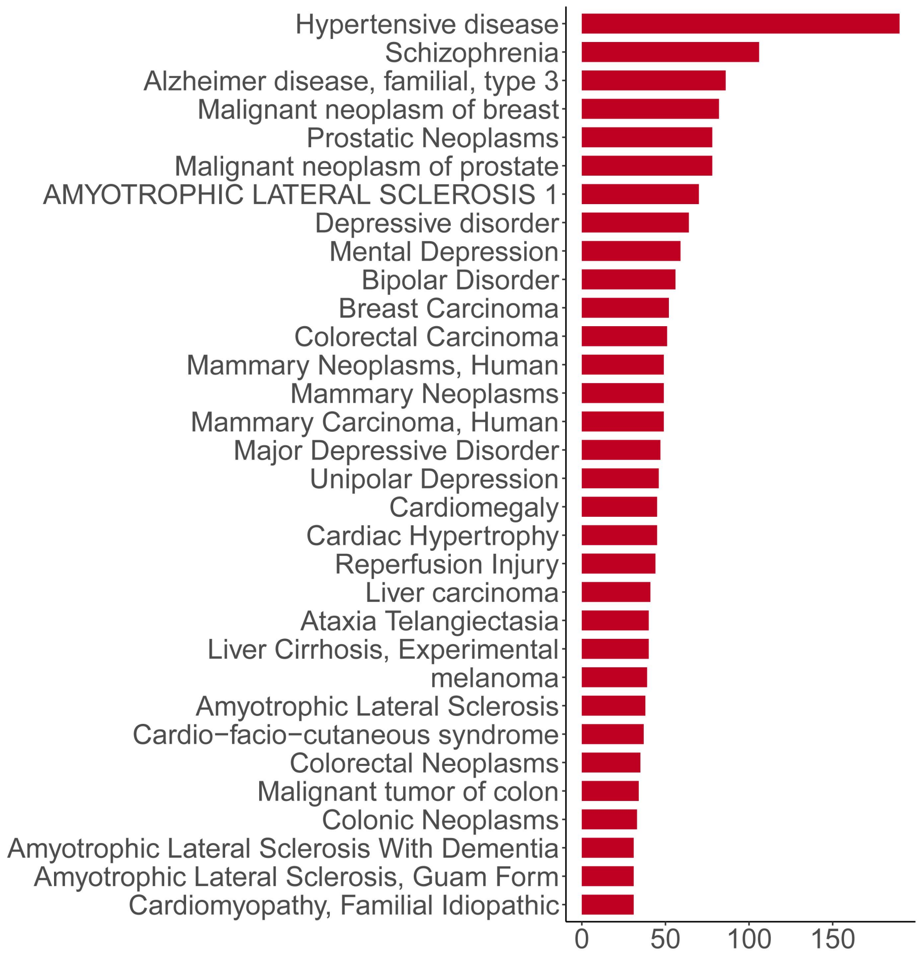

Supplementary Figure 2. The degrees of diseases in human dataset.

Supplementary Tables

Supplementary Table 2. The list of DIGs between spaceflight and ground control.

A. 569 DIGs between spaceflight and ground control groups. **B.** The DIGs in low, medium, and high dose groups. **C.** The DIGs in ten tissues.

A	DIGs
	Tef, A4galt, Sorbs3, Kif5b, Npr2, Actr8, Appl1, Mmp17, Cxcl1, Srms, Ahctf1, Mapre1, Tgfbr3, Plbd2, Anapc1, Cbx2, Ifit1, Trim24, Ttc33, Anapc4, Ankrd50, Calr, Kdm1a, Gm10263, Ephb1, Aqp7, Map4k4, Rhobtb1, Spry4, Tfrc, Plxna2, Stx6, Trrap, Pbrm1, Fem1b, Crnkl1, Dhx29, Zkscan17, Atad2b, Rxra, Ubr1, Rdh13, Dclre1a, Ctps, Dhx15, Ilf2, Npas2, Per2, Auts2, Herc2, Zbtb43, Bhlhe40, Klhl7, Otub2, Plk2, Uba5, Bmp1, Clock, Cux2, Gm14288, Kdm6a, Mettl7a1, Zbtb10, Zfat, Zfp260, Zfp386, Zfp507, Zfp532, Zfp654, Zfp68, Zfp808, Zfp972, Brdt, Serpinb6a, Cachd1, Lims2, Cdc23, Med23, Tceanc, Cct6b, Cdc27, Crebl2, Gli1, Top2b, Gzf1, Hdac2, Nkap, Clcn7, Plod1, Flot2, Hic1, Nedd9, Stag2, Zfp606, Zfp758, Zfp938, Zfp958, Dnajb11, Hnrnpd, Csnk1g1, Per3, Rps6kc1, Cstf2, Scly, Cux1, Hmgxb4, Cyb5d1, Gorasp1, Dmwd, Zfp36l2, Denr, Gemin5, Spr, Dusp3, Pdia5, Efna4, Trim23, Esrra, Exosc4, Imp3, Fasn, Fcgrt, Surf1, Fkbp9, Flt4, Ip6k2, Zmym2, Fzd8, Gmeb1, Tpp2, Mrps2, Gm10184, Ube2e1, Sf3b1, Khdrbs3, Ncbp1, Ubqln1, Ube2i, Hdac9, Zfp354b, Zfp672, Hspa5, Igfbp4, Prpf38a, Tgfbr2, Ing5, Rnf135, Kank2, Kcna6, Plekhg1, Lpin1, Lrp1, Lrrc55, Ssh2, Mcm10, Mex3c, Mogat2, Mrpl36, Mrpl39, Mrps27, Naglu, Zgrf1, Nr2f2, Nupl2, Nvl, Pcsk4, Rngtt, Top1, Ulk3, Yeats4, Yy1, Zfp865, Rnf2, Rpusd2, Tars, Rsph1, Siae, Slc37a4, Zfp467, Tprkb, Srsf10, Syncrip, Yeats2, Taf1b, Wnt5a, Usp31, Ubxn4, Map3k5, Ror1, Aftph, Agk, Phf20l1, Cdk2, Cfap97, Wdr20, Ap2b1, Ap5m1, Arid2, Ypel3, Nkapl, Ash2l, Rem1, Atp8b2, Usp54, Ppp2r3c, Gabarapl1, Bmp3, Gm10226, Cdc16, Cep78, Hells, Rad17, Rev1, Setdb1, Trib3, Npc1, Cpq, Creg2, Ranbp6, Ddx20, Ltn1, Fgf1, Dr1, Katna1, Efna2, Eif4a2, Txndc16, Frs2, Gns, Uck2, Hmga1, Hsdl2, Msh3, Zmym4, Ppfia1, Lgals9, Man1a2, Mettl16, Sdc4, Mzt1, Yipf3, Nxf1, Rad54b, Smn1, Zfp944, Wsb1, Ypel1, Pdzrn3, Csf2rb2, Oas1a, Zfp184, C130026I21Rik, Mrpl19, Slc30a4, Dusp10, Tubb2a, Ebf3, Eng, Hip1r, Sigmar1, Itga5, Kansl1l, Prkar2b, Rps20, Pnkd, Rplp2, Sec61b, Taf1d, 2010315B03Rik, Oxsm, Mybbp1a, Acadsb, Sqstm1, Pnpla3, Btnl9, Nedd4l, Robo4, Tnfrsf22, Adcy10, Eaf1, Aga, Agpat2, Cct3, Txnrd1, Gclc, Cables2, Dusp5, Insr, Clpx, Amfr, Nfil3, Angptl2, Clhc1, Reps1, Rab13, Blmh, Kat7, Cebpg, Ccdc115, Ndfip1, Ptch1, Smarcc1, Tmed4, Zfp956, Dchs1, Car7, Zranb2, Dnmt3b, L3mbtl3, Mbtd1, Pcgf6, Rbl2, Cdk1, Orc4, Irf2bp2, Cct5, Kalrn, Cidec, Zfp932, Lypd1, Snx4, Cnot7, Col11a2, Col14a1, Irf2bpl, Crbn, Cstf3, Etv6, Orc5, Ephx1, Cysltr1, Slc17a8, Rbm25, Srsf1, Rad51c, Dus1l, Med26, Spen, Hnrnpu, Ikbkg, Ppm1k, F3, Fnip1, Fntb, Meox2, Pask, Gipc3, Gucd1, Slc1a2, Nasp, Htra3, Ift172, Pcdh12, Kcnh1, Klhl13, Nsmce2, Nub1, Mrfap1, Tiam2, Zscan21, Mycl, Mylip, Ninl, Sntg2, Orc3, Rnf144b, Samd5, Poll, Traip, Stx18, Tmem177, Slc39a7, Zcchc18, Tada1, 4921524J17Rik, Acox2, Htatip2, Mical3, Gab1, Baz2a, Srfbp1, Rsbn1l,

	Ttll4, Smarce1, Chrac1, Ciita, Cnnm3, Col12a1, Dis3l2, Golga2, Peli1, Kdm5c, Stab1, Zer1, Mettl3, Sec23a, Ube2w, Cdc25c, Ctdp1, Id3, Mtmr14, Tnf, Uhrf1, Cacna2d2, Slc22a3, Ffar4, Foxk1, Sltm, Lilra5, Lmo4, Mia3, Slc7a11, Rcc2, Ythdf3, Egln3, Fbxl19, Fbxl4, Gtf2e2, Id4, Acsm1, Ino80c, Dlst, Kif4, Angel1, Tmtc4, Tbc1d17, Armc10, Zmynd11, Fancg, Pcgf5, Dusp7, Catip, Cnot6, Cd3eap, Lzts1, Zkscan4, Gmcl1, Thrap3, Eif1a, Ndufc2, Smndc1, Zfp341, Ehd2, Katnal1, Jmjd4, Exo1, Ext1, Fbxo31, Mrpl16, Ip6k3, Letm1, Wipi1, Trim12c, Mplkip, Rgmb, Tdrkh, Ube4a, Wdr86, Chac1, Cmbl, Romo1, Eif4enif1, Gbp7, Tmem241, Fbxw8, Tnrc6c, Ap3b1, Cox6b2, Lin52, Utp23, Epm2aip1, Gpx7, Zfp511, Cds2, Ctla2a, Rrp15, Cnp, Rbm15b, Fzd1, Loxl3, Glipr2, Slc6a17, Mettl8, Paip2, Slc25a40, Isg20l2, Srr, S1pr4, Cyp20a1, Mgat1, Rdh11, Orai3, Ndufb5, Slc15a4, Mettl14, Mta2, Ubap2l, Ddias, Hlx, Prdm2, Dcun1d3, Lrrc42, Slc39a6, Fam178b, Prx, Plekha4, Stard6, Jkamp, Megf8, Gck, Hepacam, Dnajc12, Hs1bp3, Tbc1d25, Mrps26, Senp6, Poglut1, Nnat, Secisbp2l, Amz2, Bloc1s6, Atg12, Slc26a1, Atp13a3, Morc3, Ndc1, Lyrm2, Nsmce4a, Sulf2, Jade1, Mid1ip1, Nubpl, Setd6, Pigf, Slc8b1, Ptgr2, Slc35f1, Azin1, C1qtnf2, Pigo, Slc39a10, Dusp14, Crispld2, Pwwp2a, Mettl4, Phlda1, Slc35e2, Plekha2, Ptgfrn, Selenoh, Spaca6, Pram1, Coq10b, Morc4, Atraid, C1qtnf1, Dap, Bglap3, Fbrs, Fam135a, Arl6ip6, Mmrn2, Amigo2, Parp16, Mtfmt, Gpam, Cntd1, Gimap8, Fam214a

**B**	**DIGs**
Low	Tef, 6720489N17Rik, Abcd1, Sbds, Acads, Acsf3, Npr2, Gpc6, Nhlrc2, Adcy1, Alg3, Rgs16, Rela, Calr, Tapbp, P2rx3, Fkbp2, Mapk15, Spry4, Stat2, Asap2, Pld6, Tnfaip2, Arg1, Smarcc2, Csnk2b, Hsp90ab1, Vamp3, Slit3, Dbp, Etv1, Atg14, Mdp1, Rdh13, Xpa, Npas2, Bhlhe40, Bhlhe41, Birc2, Casp12, Rab8a, Src, Sirt3, Zfp146, Zim1, Btbd3, Cdc5l, Catsper2, Supt16, Cars, Casr, Ifitm2, Cct6b, Dusp8, Tubb4b, Inmt, Cenpc1, Hjurp, Eif3g, Rapgef2, Clcn7, Clec5a, Cpeb4, U2af1, Cpt1a, Dtx4, Pcdhb14, Pgf, Zfp606, Per3, Ik, Cwc25, Cyb5r3, Sqle, Gorasp1, Zfp36l2, Denr, Lrp3, Rps13, Nucb1, Gch1, Men1, Dusp12, Dusp4, Map1a, Eif5a, Enpp1, Eny2, Esrra, Zfp623, Fcgrt, Fzd8, Gadd45a, Gmeb1, Map2k7, Mrps2, Mtg1, Rpl13, N4bp2l1, Trim35, Gm12166, Golt1b, Gtf2f1, Smarcd1, Prpf6, Rala, Ubqln1, Psmc1, Gna12, Sord, Gnmt, Shisa6, Hus1, Thap1, Zfp740, Zkscan7, Idi1, Ino80, Thbs4, Rnf135, Kcna6, Kcnq4, Klb, Lcn2, Proser2, Stap2, Mbd2, Mcm10, Mcu, Med12l, Mff, Mocs1, Mrpl46, Mrrf, Ncor1, Wscd2, Nrf1, Pcsk4, Pgr, Ubp1, Plcd4, Polr1b, Ppef2, Tbccd1, Ppp2r5a, Zscan26, Psmd11, Wls, Rraga, Ybx2, Snrpa, Tars, Trip10, Slc38a3, Tfe3, Zfp605, Vkorc1, Acad10, Ctr9, Paxip1, Prickle4, Ankrd37, Exoc3, Kpnb1, Arhgef17, Asb13, Ash2l, Cops8, Kdm4b, Atp6v1e1, Usp54, Mertk, Bmp3, C1qc, Tmprss6, Hint2, Scarb1, Cfdp1, Rev1, Trib3, Npc1, Krr1, Eif3b, Larp1, Trmt1, E2f8, Fcgr1, Ube2z, Txndc16, Ftsj3, Slu7, Id1, Jmjd1c, Spg20, Lhx6, Rassf10, Tex30, Scfd2, Ndrg3, Nolc1, Yipf3, Pdgfc, Sema5b, Ptrh1, Tsc1, Zfp422, Zfp775, Zfp811, Snf8, Trappc12, Trim69, Mad2l2, Ap4s1, Gmfb, Ccdc130, Hes1, Gm14443, Psmd3, Ccdc103, Cog3, Dpp9, Pdia4, Txndc11, Dnttip1, Grip2, Tubb2a, Ngfr, Mat2b, Esyt2, Tirap, Sfrp5, Golph3l, Lnx2, Hif1an, Sigmar1, Itga5, Slc7a2, Lasp1, Micu3, Nsfl1c, Rps20, Pex5, Pnkd,

	Zbtb8a, Siglec1, 5430403G16Rik, Pum3, Rrp1, Vcp, Myo1e, Ptpn9, Acsl6, Fahd1, Supt6, Ttll12, Ampd2, Frrs1, Txnrd2, Mapk8ip1, Sult4a1, Clpx, Uba1, Nfil3, Angptl6, Ccdc8, Cdk16, Obsl1, Arhgef25, Ssrp1, Ano2, Pcx, Slc2a8, Get4, Kif1a, Arhgap28, Lamtor1, Atl3, Bag2, Itsn1, Mlkl, Psmd12, Txnip, Dhx37, Bcl2l11, Bnip3l, Cops7b, Brd7, Klf16, Zfp956, Maml1, Zcchc3, Bsn, Map3k10, Cbx6, Dnmt3b, Med30, Rassf8, Chka, Doc2b, Gm12258, Zfp595, Zfp810, Chmp6, Chrnb1, Snx4, Tuba4a, Syt1, Col14a1, Lamb2, Smoc1, Irf2bpl, Csrnp1, Rbm3, Snrpb, Rnf145, Etv5, Ddx23, Impdh1, Pabpc4l, Eif3c, Dffa, Fancl, Kctd17, Dnajc21, Prpf19, Oxr1, Dock3, Piga, Hspb1, Mogs, Tarbp2, Eif3a, Elmod3, Nars, Prdm11, Rfc5, Erlin1, Ip6k1, Mphosph6, Hgf, Mapk12, Ntrk2, S1pr5, Tshr, Wac, Gpatch11, Gprc5b, Sema4c, Slc35a2, Mtpap, Pdrg1, Srxn1, Tbk1, Uqcrc1, Txndc9, Lin9, Smc1a, Slc20a1, Stk38l, Mvb12b, Mxra7, Rfc3, Ptms, Pde4c, Ntmt1, Pold2, Slc9a2, Smyd5, Tbcb, Abcd2, Ccne1, Ldhal6b, C1qa, Blcap, Bloc1s4, Hps5, Faf2, Tcea2, Usp8, Def8, Serpine2, Ppp2r2d, Stab1, Mpg, Sema6b, Rufy1, Uroc1, Adat1, Setd4, Id3, Mrc1, Slc39a2, Tpgs2, Micall2, Zfp691, Ccl24, Dcun1d4, Dnttip2, Tab2, Kcnn4, Papss2, Tspyl2, Ctdsp2, Parp9, Styxl1, Ssna1, Abcb9, Psmd7, Iars, Anapc5, Fblim1, Aplnr, Pmpca, Slc44a2, Ndufa12, Slc25a11, Atp6v0e, Atp5g1, Fancg, B4galnt1, BC005624, Dnase1l1, Caprin1, Prmt7, Cdca3, Ces2e, Fip1l1, Eif1a, Ndufc2, Ubxn1, Ubl3, Dcaf5, Kars, Dnajc1, Dync1li2, Pdcd4, Parva, Gpaa1, Stbd1, Wnk4, L3hypdh, Tlk2, Zfp207, Sav1, Slc12a7, Adamts7, Gmds, Gtf3c6, Dnah10, Kctd7, Dscc1, Haus1, Tecr, Leprot, Patz1, Slc41a3, Mcat, Usp42, Ap3b1, Sh3bgrl, Abce1, Btd, Bik, Abca4, Agtpbp1, Sgip1, Atp11c, Rrp15, Praf2, Trim44, Thoc5, Slc6a9, Tspan2, Fgf11, Slc6a17, Homez, Spon2, Vopp1, Snx32, Mgat1, Slc15a4, Fbxl22, Mars2, Hrc, Sfr1, Tax1bp1, Ciart, Nr1d2, Sco1, Mxra8, Lxn, Dgat2, Hhipl2, Immp2l, Taf1c, Ak4, Trabd2b, Slc25a19, Fbxo38, Hnrnph2, Trim26, Poglut1, Nmb, Aida, Nptn, Gnptg, Ndufa8, Polr1d, Liph, Atp13a3, Phf3, Igsf8, Zfyve27, Fam151b, C5ar2, Nubpl, Setd6, Mettl5, Slc8b1, Wfdc1, Azin1, Plekha1, Slc35e2, Rnf148, Selenoh, Nxpe4, Pet100, Abca7, Dap, Slc39a3, Gon4l, Glcci1
Medium	Sorbs3, Kif5b, Smap2, Wdr1, Fbn1, Bud13, Zfp472, Actr8, Trim41, Rnf187, Adamts20, Agfg1, Ago2, Plbd2, Pgm5, Anapc10, Ifit1bl2, Pabpc1, Plk3, Ttc33, Anapc4, Angptl4, Calr, Rpl23, Brd4, Tpd52, Rev3l, Ldlr, Grb10, Jak2, Nck2, Ncoa1, Trio, Tfrc, Psmb10, Arhgap10, Copz1, Exoc1, Gdi1, Leo1, Plxna4, Tnfaip2, Rad54l2, Chd8, Arntl, Atxn2l, Rxrb, Arpp21, Crnkl1, Eif4b, Herc3, Zkscan17, Atf3, Creb1, Ubr1, Atg13, Rdh13, Atp6v0d1, Efr3b, Mcm4, Rp9, Npas2, Per2, Auts2, Herc2, Bcor, Zbtb43, Fmod, Klhl7, Lrrn1, Smad3, Uba5, Dhdds, Ep300, Zbtb10, Zfat, Zfp146, Zfp462, Zfp507, Zfp719, Zfp972, Brd8, Cdc5l, Mcoln1, Casq2, Cbfa2t2, Cbl, Ccdc47, Cdc27, Wee1, Ciao1, Cdkn1a, Mdm2, Cenpc1, Top2b, Hic1, Pcdh18, Zfp579, Srrm1, Per3, Csnk1g3, Rps6kc1, Jrk, Nop14, Cuta, Hmgxb4, Cyb5d1, D2hgdh, Zfp36l2, Ddx6, Dnmt3a, Glt28d2, Dusp1, Ehhadh, Hyi, Ep400, Ptpn4, Trim23, F2rl3, Fam110b, Lrp6, Fcgrt, Ptpn11, Fhl2, Fkbp9, Ip6k2, Fundc1, Gadd45a, Gmeb1, Llph, Rpf2, Mepce, Prpf8, Pum1, Sugp1, Prokr1, Tdrd3, Hdac9, Rad50, Zfp672, Ift46, Ttc30a1, Ing5, Ints9, Islr, Slc16a1, Lamc1, Ncstn, Scaf4, Setd1b, Stau1, Klf3, Lpin1, Trim14, Mrps27,

	Ncor1, Nid1, Wdr45, Xpo1, Optn, Shmt1, Pck1, Pde12, Rgp1, Rhbdd2, Phf6, Pigc, Rngtt, Ppil1, Top1, Zscan26, Rassf1, Trmt11, Sec14l4, Tufm, Rpl4, Zfpm2, Vcl, Srsf10, Actl6a, Ago4, Aktip, Anapc15, Ankib1, Ypel3, Ecm2, Bcl6, Phf19, Gm10226, Kdm4d, Cdc16, Wdr19, Cd209a, Hint2, Epc1, Fos, Ing3, Kat8, Pole3, Zfp110, Npc1, Trmt1, E2f8, Pwp1, Gfm1, Gigyf1, Tnrc6b, Gm20695, Ighmbp2, Gtpbp3, Grb14, Hck, Zfp142, Zfp526, Hmga1, Hook2, Zfr, Iqank1, Kctd9, Uggt1, Man1a2, Mettl16, Mrm1, Nacc2, Shf, Pdzd8, Ung, Utp6, Zfp568, Zxdc, Smn1, Wsb1, Hspb11, Ap1g2, Smtn, Rps29, Oas1a, Rnf19a, Mrpl19, Cog3, Dusp10, Grip2, Mob2, Tubb2a, Eif2s3x, Fign, Pdcd2l, Gp5, Mx1, Leng8, Prkar2b, Ngf, Nup50, Ppig, Zbtb24, Zbtb32, Rbm4b, Zyg11b, 2810021J22Rik, 3110082I17Rik, Mybbp1a, Nfx1, Utp15, Myo1e, Sqstm1, Snx25, Actg1, Lefty1, Nhlrc3, Spryd4, Tdp2, Tnfrsf22, Trim39, Trim7, Map1lc3a, Eaf1, Ahrr, Ppard, Gclc, Arl15, Cables2, Ppl, Zranb1, G3bp2, Nfil3, Amt, Angptl2, Lmo7, Cdc37l1, P2rx7, Pcx, Cebpg, Atg4a, Atp11b, Bag1, Hsf2, Larp4b, Nqo1, Txnip, Bbs10, Ccng2, Stat5a, Zfp956, Nsun6, Rbm34, Zranb2, Suv39h1, Ccar1, Ccdc84, Orc4, Irf2bp2, Cdc45, Cidec, Gm12258, Gm2a, Lypd1, Cnbp, Irf2bpl, Socs5, Etv6, Flcn, Rbm3, Ephx1, Desi1, Gtpbp8, Npm1, Rsph3a, Slfn1, Dnajc2, Sema4b, Dtwd2, Med26, Hnrnpu, Tubgcp2, Kdsr, Ppp2r5c, Rhoc, Rnf8, Tk2, Fgd3, Vstm4, Fnip1, Slc25a20, Gsr, Hinfp, Rbm39, Tcf24, Plekhf1, Usp4, Zrsr1, Pmpcb, Tbk1, Nasp, Sox9, Lamp2, Pdgfb, Nup62cl, Irak2, Morf4l1, Nub1, Mier3, Mrfap1, Ppp1r10, Zfp658, Scyl3, Podxl2, Pold3, Tmem203, Ppp1r15b, Stambpl1, Vapa, Usp1, Tmem63a, Wrap53, Zswim6, Abcd2, Acad9, Acox2, Htatip2, Ipo8, Afg3l1, Ppp1r3c, Bace1, Gab1, Plcg2, Bcl2l2, Srfbp1, Tbc1d9, Ces1f, Rcan2, Cnnm3, Ctsk, Def8, Ovca2, Filip1l, Foxp1, Irgq, Itga8, Zer1, Wdr27, Myo1f, Neil2, Pigk, Ube2w, Tle4, Apobec1, Gskip, Cd300lf, Ext2, Fst, Mtmr14, Tnf, Vash2, Ube2f, Poc1b, Yipf2, Rarb, Snca, Med9, Dcun1d4, Eda, Eid2b, Ets2, Foxk1, Loxl2, Hsd3b2, Nr3c2, Rcc2, Rnf122, Ambra1, Btg2, Flt3, Egln3, Zfp830, Fbxl4, Id4, Tbcd, Nelfb, Uri1, Yod1, Abcb9, Mthfd1l, Acer3, Dlst, Adam9, Nt5c2, Angel1, Aldh1l2, Taco1, Tbc1d17, Armc10, Yipf5, Zmynd11, Elovl7, Fancg, Bcas3, Dusp7, Cenpb, Fip1l1, Cilp, Cisd3, Spsb3, Tbc1d31, Slc25a12, Cox7a1, Ubxn1, Mrpl45, Kcnj8, Klhl22, Cystm1, Srsf7, Mettl2, Noc2l, Zfp655, Orai1, Oma1, Enc1, Ext1, F8a, Fam189b, Rdh14, Ltbp2, Snupn, Gpaa1, Rtn4ip1, Lyar, Nfya, Jade2, Tspear, Ssc5d, Letm1, Man1a, Ppp1r3f, Slc25a30, Tacc3, Pm20d1, Rbm7, Sbf1, Tbc1d12, Znhit6, Rwdd1, Sgcb, Coq2, Agl, Gtf3c6, Slc2a6, Eif4enif1, Gbp3, Mgat5, Plekhm1, Ndufa4l2, Zfp668, Prpf40a, Prpf40b, Tnrc6c, Eps8l1, Utp23, Timm29, Cdca4, Zfp747, Steap3, Acin1, Klk8, Astn2, Slc30a6, Cnp, Cog7, Tppp3, Lpl, Ptcd3, Taf6l, Kansl1, Xaf1, Slc25a33, Plin2, Gins4, 2310022B05Rik, S1pr4, Nufip1, Orai3, Slc15a4, Tmem216, Crtc3, Dck, Srrm2, Fancf, Pacsin3, St3gal4, Bbox1, Cdkn2aip, Celf1, Sash1, Slc39a6, Lpgat1, Cmtm4, Rnf149, Ncmap, Sco1, Zrsr2, Rfx5, Zc3h12c, Fam107a, Slc19a3, Timd4, Lamtor5, Ubiad1, Slc25a34, Loxl4, Mtmr11, Ccndbp1, Qrich1, Arfip1, Amz2, Bloc1s6, Ttl, Cox19, Tbc1d22a, Mrpl55, Nt5dc1, Tfpt, Trp53inp2, Isca1, Slc26a1, Atp13a3, Ypel5, Gjb1, Cdc42ep2, Zbtb8os, Jade1, Nat9, Rmnd1, Slc25a39, Pigm, Mid1ip1, Pex14, Ece1, Pigf, Ptgr2, Arsj, Azin1, Pigo, Lclat1, Ggcx, Slco1a4, Pex16, Coq10b, Cep162, Rnf217, Scaper, Abhd17a,

	Cnnm4, Tmem214, Cntd1, Alkbh4, Ccdc134, Pxdc1
High	2610044O15Rik8, Prkdc, A4galt, Slc15a2, Abhd6, Abi2, Cdk7, Mapk14, Igf1r, Kdelr1, Sod1, Smarcad1, Sp4, Zfp729a, Zik1, Acvr1c, Appl1, Adamts3, Mmp17, Adra1b, Srms, Agtr1a, Nup93, Aif1l, Akr1a1, Tgfbr3, Cyp2d22, Anapc1, Trim24, Anapc4, Angel2, Anks4b, Calr, Kdm1a, Foxo3, Gm10263, Ephb1, Aqp7, Map4k4, Rhobtb1, Spry4, Stat2, Synrg, Arap2, Igf2r, Atr, Gorasp2, Plxna2, Asap2, Dnaja1, Leo1, Pitpnb, Stx6, Prodh, Arhgap32, Pbrm1, Shprh, Chd6, Arntl, Ascc1, Dhx29, Tsku, Asf1a, Atad2b, Pou2f1, Atp6ap1, Cers2, Dclre1a, Mllt3, Ctps, Dhx15, Ilf2, Zfp407, Otub2, Plk2, Bmp1, Bmyc, Boll, Chd4, Gm15446, Kdm6a, Mettl7a1, Srbd1, Zbtb1, Zfat, Zfp260, Zfp280b, Zfp322a, Zfp329, Zfp386, Zfp518b, Zfp532, Zfp566, Zfp626, Zfp644, Zfp68, Zfp799, Brd8, Brdt, Jarid2, Kdm5b, Setbp1, Phf20, Ppp4r4, Tcerg1, C1ra, Serpinb6a, Serpinb9b, Cachd1, Lims2, Car5b, Rhbdl3, Kdm2b, Cdc23, Tbck, Ccne2, Med23, Tceanc, Lurap1, Dusp8, Crebl2, Gli1, Mdm2, Dynll2, Serping1, Gtf2b, Top2b, Gzf1, Hdac2, Nkap, Taf5, Zfp955a, Chml, Eps8, Chrna7, Chtop, Clasp1, Clec3b, Uba2, Plod1, Tor1a, Cpsf3, Mynn, Stag2, Zfp606, Zfp758, Zfp763, Zfp958, Dnajb11, Hnrnpd, Pspc1, Csnk1g1, Ctdspl2, Cux1, Dcaf11, Hmgxb4, Cyb5d1, Nfic, Tmed5, Nmt1, Dclk1, Gpatch2l, Dhcr24, Spr, Pdlim3, Dusp3, Eci2, Pdia5, Epha2, Mnat1, Exosc4, Exosc7, Imp3, Fasn, Fbxw7, Fcgrt, Ptpn11, Fkbp9, Flt4, Lef1, Zmym2, G2e3, Gabpb2, Gadd45a, Itm2b, Nup205, Gmeb1, Stt3b, Gm14305, Ube2e1, Gm4353, Pop4, Riok1, Sf3b1, Khdrbs3, Ncbp1, Ptbp2, Rbm28, Tnpo3, Nras, Rrad, Hus1, Ube2i, Siah1b, Zfp334, Rcor1, Xpo5, Zfp27, Zfp354b, Hmgb1, Hspa5, Igfbp4, Prpf38a, Tgfbr2, Ncbp3, Kng2, Mmp28, Mmp3, Tenm4, Lrp12, Osmr, Rnf135, Kank2, Kcna2, Plekhg1, Lman2, Lpcat3, Lsm8, Pdcd6, Mdn1, Mex3c, Pex11a, Mogat2, Mrpl34, Zgrf1, Nin, Ncor1, Nr2f2, Pla2g7, Tor1b, Nupl2, Nvl, Nynrin, Obscn, Papolg, Zeb1, Tox4, Pold4, Ptpro, Prg4, Prkab1, Sun1, Top1, Yy1, Zfp865, Psme1, R3hcc1l, Snrnp40, Rmi1, Tox, Rnf167, Rpusd2, Tars, Wdr33, Rtca, Tomm22, Sap30, Siae, Slc37a4, Tbpl1, Tfe3, Zbtb26, Zfp157, Zfp160, Zfp273, Zfp397, Zfp738, Zfp748, Zfpm2, Tprkb, Zfp687, Syncrip, Yeats2, Taf1b, Wnt5a, Tm7sf2, 4930590J08Rik, Ror1, Actr6, Add3, Agk, Wdr5b, Usp38, Aktip, Taf9, Cdk2, Cfap97, Samd1, Tnrc6a, Wdr20, Ankib1, Ankrd28, Ap2b1, Ap5m1, Arid2, Drp2, Asb5, Nkapl, Srsf6, Ash2l, Asxl2, Rem1, Atp8b2, Ppp2r3c, Bclaf1, Gabarapl1, Ube2g1, Brca1, Pgbd1, Prdm15, Usp7, Cep78, Hells, Phf10, Rad17, Setdb1, Zfp874b, Gstcd, Coil, Gm10509, Vwa1, Zfp715, Creg2, Kdm2a, Lgals3bp, Ranbp6, Cybrd1, Ltn1, Ppwd1, Fgf1, Katna1, Eif4a2, Ercc6, Ercc6l, Wtap, Frs2, Gde1, Gns, Uck2, Hexa, Prdm10, Ttf2, Zfp512, Hnrnpc, Hsdl2, Hspa14, Msh3, Irgm1, Zmym4, Ppfia1, Lgals9, Tnfaip6, Lztfl1, Sdc4, Sesn3, Mztl, Tex30, Nacc2, Nanp, Slc8a1, Nxf1, Vwa8, Pcf11, Rad54b, Supt20, Zfp433, Zxdc, Senp1, Stx16, Top3b, Vta1, Acly, Actn2, Zfp944, Adora2b, Agtrap, Anapc16, Hspb11, Sumo1, Flnc, Pdzrn3, Csf2rb2, Irs3, Jazf1, Zfp184, Bclaf3, C130026I21Rik, Dusp13, Mob2, Ebf3, Eng, Mat2b, Zbtb14, Xrcc5, Sfrp5, Gpatch1, Gsn, Hip1r, Lsm3, Prpf18, Kansl1l, Mex3a, Ppp1r3b, Zfp770, Zfp954, Taf1d, A430033K04Rik, Oxsm, Rab3ip, Maged1, Mpdz, Slc27a1, Acaca, Acadsb, Sqstm1, Pitrm1, Pnpla3, Emd, Tubb6, Capn3, Stk32c, Btnl9, Fgfr1, Gdf10, Robo4, Adcy10, Cdkn1b, Agpat2, Slc4a9,

	Cct3, Gclc, Becn1, Cyfip2, Insr, Mapkapk2, Sh2b2, Sult4a1, Aldh18a1, Als2, Amfr, Nfil3, Nfe2l1, Csrp3, Aoc1, Aox1, Reps1, Tia1, Bbip1, Iqsec2, Rab13, Sipa1l1, Blmh, Kat7, Ebf4, Nhlrc1, Ptch2, Ttn, Txnip, Ube2d3, Zc3h7b, Isy1, Snip1, Bcdin3d, Smarcc1, Tmed3, Zw10, Zmym3, Chaf1a, Tfec, Tmed4, Zbtb18, Zfp101, Stim1, Cacna1g, Catsperg1, Dchs1, Slc3a2, Ewsr1, Zranb2, H3f3b, L3mbtl3, Mbtd1, Pcgf6, Rbl2, Orc4, Cep57, Med30, Cdk4, Cidec, Gabpa, Zfp85, Zfp866, Zfp932, Sec23b, Clcn5, Cldn2, Kif24, Tnfrsf21, Cnot7, Col11a2, Col14a1, Crbn, Cryz, Cstf3, Tshz2, Orc5, Slc3a1, Cyfip1, Cyp1a1, Zfand2a, Dctn5, Elf2, Ilf3, Fancb, Rassf6, Rnaseh2b, Dus1l, Timp3, Psmd5, Xxylt1, Egfem1, Slc18a1, Emc4, Hnrnpu, Pde11a, Nars, Erc1, Ppm1k, Ppp2r5c, Fbp1, F3, Pla2g5, Meox2, Lrp2, Vangl1, Slc6a4, Umps, Slc25a20, Gda, Pask, Gpr39, Iqsec1, Gpbp1l1, Gsr, Zfp873, Zfp995, Nek9, Msc, Hinfp, Jdp2, Mesp2, Hoxd4, Map3k15, Myo15, Slc1a2, Tra2b, Ribc1, Htra3, Htra4, Hspbap1, Mettl6, Pcdh12, Inppl1, Irak2, Klhl13, Shcbp1, Star, Mab21l2, Nsmce2, Sar1b, Map1lc3b, Nub1, Pkp4, Msl1, Zscan21, Mycl, Prox1, Mylip, Ndufb9, Tfdp1, Nrp2, Orc3, Osbpl11, Pros1, Rnf144b, Samd5, Sytl5, Pole2, Poll, Traip, Vapb, Stx18, Tmem177, Slc35a4, Tada1, Timd2, Tgfa, 2310011J03Rik, 4921524J17Rik, Kcnj15, Adora3, Pitpna, Pipox, Gab1, Srfbp1, Rsbn1l, Sfmbt2, Sirt1, Ttll4, H2-M2, Mtbp, Smarce1, Usp8, Chrac1, Cnnm3, Col12a1, Eri2, Dis3l2, Itm2a, Zdhhc24, Golga2, Ifnar1, Kbtbd12, Kdm5c, Megf6, Ppp2r2d, Stab1, Mettl3, Rhno1, Pcdh20, Sema6b, Vamp1, Rai14, Ccnb1, Clec10a, Emc3, Polr3d, Rab11fip3, Serpina3i, Ackr1, Dtd2, Arglu1, Npdc1, Rarb, Zfp691, Zfp872, C1qtnf9, Cacna2d2, Cenpu, Col20a1, Cspp1, Slc22a3, Sltm, Igfbp3, Slc7a11, Znhit3, Aldh8a1, Cpsf6, Cpsf7, Chmp7, Fbxl4, Parp9, Tomt, Gtf2e2, Tmem245, Prc1, Ryr1, Glyr1, Acsm1, Dlst, Kif1c, Ankrd40, Akirin2, Pcmt1, Stac3, Atl2, Erlin2, Usp13, Pcgf5, Med10, Catip, Gpcpd1, Svil, Washc5, Pcyt1a, Rmnd5a, Cd3eap, Cyb5d2, Rnf4, Lzts1, Zkscan4, Zfp768, Clcn1, Gmcl1, Thrap3, Cnep1r1, Kcnj8, Srsf7, Klhl41, Fermt2, Ndufv2, Ehd2, Jmjd4, Exo1, Rdh14, Ltbp2, Fnbp4, Il12rb2, Pex3, Pip4k2c, Ormdl2, Ip6k3, Kcnu1, Kif13a, Wipi1, Trim12c, Primpol, Swsap1, Mplkip, Sec14l5, Rnf10, Rgmb, Rbm7, Tdrkh, Rnf138, Wnk3, Wars2, Fbxw2, Fndc3a, Cmbl, Gas2, Gbp7, Tmem64, Wdyhv1, Gid4, Fbxw8, Galnt15, Slc16a7, Cox6b2, Lin52, Morn4, Slc35e1, BC055324, Epm2aip1, Cdca4, Fgfr1op2, Gpx7, Ska1, Glis1, Fam214b, Hnrnph1, Cds2, Slc4a8, Aldh16a1, Plp1, Ctso, Bora, Cln6, Wdr55, Rbm15b, Loxl3, Hr, Timm10, Mettl8, Paip2, Phospho2, Tecpr2, Bfar, Bves, Vpreb1, Mcm8, Isg20l2, Gins4, Trappc2l, Olfm4, Myrf, Nudt19, Clns1a, Nol4l, Cyp20a1, Cnih1, Ndufb5, Slc15a4, Slc31a2, Mettl14, Ano9, Mta2, Ubap2l, Hnrnpll, D11Wsu47e, Ddias, Hlx, Rasa3, Supt4a, Prdm2, Slc22a15, Xpo4, Lrrc42, Cdkn2aip, Tdg, Syce2, Fam178b, Phospho1, Smpd4, Entpd2, Thap2, Lox, Itga2, Galns, Osbpl3, Ifitm6, Glb1l2, Alox5, Ammecr1, Anks6, Bnc2, Gck, Ceacam2, Cdkl1, Hepacam, Timm21, Cys1, Dzip1l, Gm13305, Evc, Fgl2, Gcnt1, Hal, Matr3, Ldhd, Pigs, Ube2d2a, Slc22a4, Slco3a1, Zfp704, Gpr137b, Senp6, Nnat, Bend7, Atg12, Prr14l, Slc26a11, Nudt13, Ppfibp1, Morc3, Nsmce4a, Sulf2, Ccdc90b, Nod2, Zdhhc14, Tmem70, Slc35f1, C1qtnf2, Slc39a10, Phyh, Agbl2, Dusp14, Crispld2, Pwwp2a, Hnmt, Mettl4, Prkd3, Ubl5, Plekha2, Ptgfrn, Rnase4, Creld2, Cep162, Morc4, C1qtnf1, Vgll3,

	Fam135a, Tmx2, Cd248, Tymp, Arl6ip6, Mmrn2, Ggn, Armcx5, Churc1, Mtfmt, Chst11, Hmgn2, Fam214a

**C**	**DIG**
Thymus	Gstt3, Rpl27, Cdk7, Map3k2, Pdgfra, Ptk2, Rasl11a, Rras, Socs4, Sorbs3, Vav3, Pik3ca, Sec24d, Gzmm, Smap2, Acsf2, Npr2, Smarcad1, Sp4, Zfp729a, Zik1, Actr10, Acvr1c, Appl1, Lrig2, Mid1, Pdpk1, Smad5, Tmem184b, Aldh2, Gpld1, Adap2, Adcy1, Entpd1, Adra1b, Frmd4a, Adrb3, Afap1, Srms, Iws1, Agap1, Ahctf1, Nup93, Usp34, Tlr4, Akt3, Ifnar2, Lta4h, Alkbh8, Alyref, Anapc1, Eml1, Nap1l5, Pabpc1, Tep1, Trim24, Ubc, Wdr37, Anapc4, Angel2, Ank3, Ankef1, Ankrd50, Ankrd6, Anpep, Heatr5b, Kdm1a, Phlpp2, Rpl23, Arrb1, Rev3l, App, Psen2, Aplf, Aqp7, Fn1, Gab2, Gdf11, Grb10, Kirrel, Map3k1, Nck2, Rhobtb1, Sh3gl3, Spry4, Traf3, Chn2, Cav1, Plxna2, Tgfbr1, Ccm2l, Cdkn2b, Rab3il1, Stx6, Tnfaip2, Arg2, Grb2, Kdm4c, Pbrm1, Shprh, Arid4b, Mysm1, Asb3, Fem1b, Utrn, Foxs1, Specc1l, Ascc3, Cdc40, Crnkl1, Dyrk1a, Gstt1, Hnrnpab, Lrch3, Lrrc8b, Lsm2, Tril, Tsku, Asf1a, Zfp84, Atad2b, Atf5, Creb1, Dbp, Meox1, Nr2c2, Rxra, Ubr1, Ubxn7, Atg14, Atg2b, Pdik1l, Ube2j2, Ppm1d, Atp6v0e2, Dclre1a, Lama2, Mllt3, Taf7, Zfp521, Dhx15, Erh, Ilf2, Rc3h2, Avl9, Bcl2a1a, Bcl11a, Casp12, Plk2, Ppil2, Rnf144a, Rnf19b, Src, Tanc1, Bmp1, Gstm1, Nbas, Brcc3, Brwd3, Fem1c, Gli2, Gm15446, Kat6b, Kdm6a, Mef2a, Nfatc4, Spopl, Taf1, Taf2, Zbtb1, Zbtb7c, Zfp131, Zfp146, Zfp26, Zfp260, Zfp280d, Zfp322a, Zfp329, Zfp354c, Zfp386, Zfp51, Zfp518a, Zfp532, Zfp626, Zfp644, Zfp654, Zfp667, Zfp68, Zfp719, Zfp780b, Zfp786, Zfp799, Zfp81, Zfp940, Zfp955b, Zfx, Brdt, Kdm5b, Usp46, Btaf1, Dusp23, Btbd3, Cep97, Lgr5, Myb, Nktr, Ppil4, C1ra, Col8a1, Cables1, Scarf1, Tmx3, Lims2, P4hb, Casp4, Ifitm2, Cbfa2t2, Cdc23, Ccl11, Ccne2, Ccnh, Med23, Tceanc, Ccr1, Ccs, Cd209b, Cd209f, Tcam1, Cdc27, Cdc42bpb, Cdc73, Ppp2r2b, Tanc2, Ttbk2, Ciao1, Cdkn1a, Cidea, Gli1, Hivep2, Ikzf5, Mdm2, Serping1, Chd1, Gtf2b, Mphosph9, Top2b, Trim33, Hdac2, Mecp2, Nkap, Suv39h2, Taf5, Zfp955a, Chml, Chtop, Snx30, Clcn7, Clec5a, Uba2, Clmn, Nr5a2, Thrb, Epn2, Snx9, Col24a1, Lamc3, Pcolce, Pdgfrb, Plod1, Col6a3, Col6a5, Ercc8, Comt, U2af1, Dach1, Med17, Mynn, Pcdhb11, Stag2, Zbtb6, Zfp40, Zfp426, Zfp606, Zfp758, Zfp763, Zfp952, Zfp958, Ddx47, Dnajb11, Hnrnpa2b1, Lrrc1, Ythdc2, Sik2, Srsf2, Fhl4, Csmd1, Per3, Cstf2, Ctbp2, Ctdspl2, Ctla4, Ctnnd1, Exoc5, Klhl15, Klhl9, Hcar1, Lpar1, Phf11a, Rab33b, Cyb5r3, Nfic, Dap3, Gorasp1, Ndrg2, Scd2, Dcc, Dclk1, Xrcc4, Dcun1d5, Ddah1, Ddb2, Ddx3x, Dennd4c, Derl3, Dhx36, Dicer1, Dip2a, Dip2b, Disc1, Tceal1, Pafah1b1, Shpk, Nucb1, Dnajc22, Dnajc6, Zfp563, Vps45, Dusp1, Dusp19, Lmcd1, Dusp3, Lrrcc1, Pum2, Ebi3, Pdia5, Sil1, Eea1, Eef2, Ehhadh, Xrn1, Eif4e3, Ints12, Emg1, Epc2, Epha2, Rgs3, Tollip, Gtf2h1, Gtf2h4, Mnat1, Fosl2, Erp44, Exoc2, Jcad, Rcl1, Fabp4, Fam118a, Fasn, Zbtb7b, Zfp623, Gfra4, Plcl1, Ptpn11, Fignl2, Surf1, Fkbp9, Fkbpl, Flt4, Zmym2, G2e3, Pot1a, Gata2, Gbp4, Smad2, Gmeb1, Tpp2, Mxi1, Rpf2, Gm10221, Gnptab, Ube2t, Gm14305, Irf4, Pkp2, Smarcd3, Ube2e1, Ube2e3, Gm4631, Naa15, Pop4, Riok1, Rpp30, Sf3b1, Snrpb2, Snrpd1, Lrrk1,

	Ncbp1, Ptbp2, Kras, Litaf, Nras, Rasl12, Rhoj, Rhou, Sorl1, Gm5900, Mrps18b, Pgam2, Rps4x, Gna12, S1pr3, Gstm2, Olfm1, Tmbim6, Pde1b, Pde3b, Nelfe, Rnf17, Ube2i, Hcfc2, Hdlbp, Heg1, Ran, Rpa3, Traf6, Hfe, Hip1, Siah1b, Peg3, Zfp148, Zfp334, Hltf, Zfp12, Zfp27, Zfp286, Zfp316, Zfp516, Zfp651, Zfp672, Hmgb1, Hmgb2, Lemd3, Hnrnpr, Zcchc9, Ier3, Rasa1, Igfbp4, Prpf38a, Rictor, Il17rd, Tgfbr2, Rab31, Pura, Imp4, Ints6, Ints8, Ncbp3, Lrp12, Rnf135, Rnmt, Kank2, Pagr1a, Myef2, Myo3b, Prickle2, Syne2, Klf3, Klf7, Plekhg1, Lpin1, Rab14, Snrnp25, U2surp, Lsm8, Maged2, Mavs, Mex3c, Mogat2, Mrpl24, Mrpl38, Naalad2, Vamp4, Nin, Nfix, Nrip1, Uba3, Nup107, Nup35, Nup37, Nup43, Nup88, Rpgr, Tpm4, Nynrin, Olfml2a, Tex10, Senp7, Timm50, Papolg, Sncg, Zeb1, Pde12, Pex7, Zfp62, Zfp882, Traf5, Rchy1, Tcf7l1, Tox4, Plekhj1, Pnrc2, Pold4, Polr1b, Rngtt, Ttc3, Polr3k, Rif1, Ppp6r3, Prg4, Uaca, Yeats4, Yy1, Zfp697, Zfp865, Zfp93, Zfp963, Snapc3, Ptger3, Rassf2, Rad21, Rbp7, Snrnp40, Snrpa1, Rasa2, Smc5, Rassf1, Rmi1, Tox, Rlf, Rnf167, Rnf2, Wdr33, Tomm22, Usp15, Trip10, S100b, Scaf8, Serpini1, Trim30d, Tbpl1, Tfe3, Zbtb26, Zbtb33, Zfp128, Zfp157, Zfp160, Zfp273, Zfp35, Zfp369, Zfp397, Zfp605, Zfp738, Zfp748, Zfp846, Ttc39c, Suz12, Snrpc, Tlr6, Vcl, Stam, Srsf10, Ssbp2, Syncrip, Tardbp, Taf1b, Wnt5a, Thra, Aak1, Wnt2b, Ace, Actl6a, Actr6, Adam19, Adcy2, Add3, Ccnt1, Siah1a, Aftph, Agk, Ago3, Ago4, Lbr, Phf20l1, Aif1, Cyp1b1, Amotl1, Taf9, Cdk2, Wdr20, Ankrd10, Ankrd28, Ankrd37, Ap5m1, Ap4m1, Appbp2, Pkhd1l1, Arhgap6, Arid2, Slc2a10, Lta, Hipk2, Lrmda, Srsf6, Tlr5, Wdr70, Atf2, Pms1, Rem1, Rpa1, Tmpo, Rc3h1, Clec4d, Ppp2r3c, Bclaf1, Bgn, Cd40, Gabarapl1, Ppid, Ube2a, Gm10226, Srpk2, Usp7, Zfp708, Kdm4d, Cdc16, C1rl, Hpn, Prss36, Ccl7, Cxcl9, Ccnl1, Cd209d, Cd68, Cfp, Epc1, Ercc4, Hmg20a, Ing3, Phf10, Rad17, Rev1, Setdb1, Xrcc3, Zfp874b, Eif2ak4, Clk4, Cnn2, Igf2bp3, Cog5, Coil, Gtf3c3, Gm10509, Pcdhb10, Zfp715, Zfp868, Lgals3bp, Ppp3cb, Ranbp6, Dcaf13, Dclre1b, Ddx20, Ppwd1, Snrpe, Tlr2, Dnaaf2, Ryr3, Tg, Otud5, Fgf1, Dr1, Fmr1, Katna1, Ebf1, Efcab7, Efnb1, Eif1b, Eif2a, Fabp7, Fes, Fkbp10, Wtap, Frs2, Gadd45g, Gfi1, Gmps, Luc7l3, Paqr6, Gns, Pla2g2d, Gpr183, Gss, Gstp1, Gtf2h2, Rac2, Zzz3, Polr1e, Tspyl4, Zfp423, Zfp512, Hnrnpc, Rnf34, Rheb, Zfr, Skp2, Ipo11, Irs1, Pign, Urb1, Myh10, Zmym4, Ppfia1, Kin, Lhx6, Tln2, Slc9a7, Neo1, Rbm22, Pcsk6, Rspo1, Zbp1, Lztfl1, Man1a2, Map3k7, Mbip, Mcoln2, Phyhip, Msh2, Mtf2, Sbf2, Muc16, Myo1g, Mzt1, Tex30, Nacc2, Ndrg3, Rhof, Nxf1, Vwa8, Pcf11, Pcsk1, Tead4, Sike1, Ppp1r35, Rabl2, Ranbp1, Ryk, Supt20, Tbp, Tlr1, Zfp13, Zfp182, Zfp280c, Zfp39, Zfp422, Zfp433, Zfp454, Zfp820, Zfp831, Wnt2, Rtn2, Smcr8, Rangrf, Rubcn, Senp1, Smn1, Stx16, Vta1, Tcf12, Tle1, Strbp, Itgax, Acly, Zfp944, Mmp14, Adipoq, Wsb1, Agpat5, Aldh1a2, Hspb11, Nphp3, Wdr77, Ankmy2, Dock1, Cdc42bpa, Cyth4, Fgr, Ncf4, Sumo1, Ypel1, Lcp1, Ltb, Pdzrn3, Smtnl2, Wwtr1, Tbx21, Csf2rb2, Foxp3, Irs3, Lsm14a, Zfp184, Bclaf3, Blk, Btk, Rnf165, Snw1, C130026I21Rik, Cfd, Cabp4, Calm2, Ccl2, Ccl5, Ccr2, Ccr6, Cd180, Cd22, Cd274, Cd72, Cd79a, Eomes, Zfp30, Clvs1, Cmklr1, Slc30a4, Cog3, Csf2rb, Csnk1a1, Rpap2, Cx3cr1, Cxcl13, Cxcr3, Cxcr5, Folr2, Gna14, Gng10, Gng2, Gpr176, Hrh2, Itgam, Lsp1, Opn3, Ptgir, Nfyb, Phf11b, Sema4a, Dclre1c, Ddx50, Pld4, Dnajb2, Evl, Dusp16, Vwce, Ebf3, Ehd1,

	Eng, Pax5, Mat2b, Pid1, Osbpl5, Exosc3, Fcer1g, Fgf9, Zbtb14, Mrpl3, Nmd3, Gm6563, Hip1r, Lsm3, Pml, Phf14, Zfp664, Pou2f2, Prpf18, Hnrnpk, Sigmar1, Mapk8, Plin1, Itgb7, Kank3, Kansl1l, Laptm5, Prkag3, Lpxn, Lrrc32, Mat2a, Med14, Mfap5, Serpina3f, Serpina3g, Myl12b, Naa16, Nek6, Nfkbie, Ngf, Stat1, Polr1a, Rab39, Tinf2, Zbtb24, Zbtb32, Zfp281, Zfp296, Zfp770, Zfp954, Zfp983, Ptpn6, Taf5l, Trim5, Rbm4b, Taf1d, Rtkn2, Slc2a1, Top1mt, Trim34a, Myo1d, Npr1, 2610021A01Rik, 2810021J22Rik, A530032D15Rik, Maged1, Nol6, Nop56, Rpf1, Dlg5, Mpdz, Msn, Abcd3, Myo1e, Ppp2r2a, Acaca, Spns2, Rtn1, Ackr2, Cpt1c, Nr4a1, Tubb6, Tube1, Myo7a, Acvr1, Tie1, Tnfrsf10b, Trim27, Chrd, Dab2, Gdf10, Gnat2, Itch, Itgb5, Pten, Robo4, Tdp2, Tnfrsf22, Kdr, Adamdec1, Adcy10, Pde9a, Ccnt2, Eaf1, Cdkn1b, Ncbp2, Agpat2, Cisd2, Ovgp1, Akirin1, Clk1, Txnrd1, Clk3, Dlc1, Gpr55, Il5ra, Itgb1, Kank1, Mapk1, Mapk8ip1, Mapkapk2, Naip5, Plcb1, Tek, Gusb, Rdh1, Zc3h14, Alpl, Amn1, Amotl2, Card11, Magi1, Nfil3, Taf12, Angptl1, Lmo7, Sh3yl1, Arhgef25, Pcna, Nfe2l1, Shank3, Xrcc6, Spast, Ubox5, Antxr2, Col5a1, Dag1, Itgb3bp, P2rx7, Stxbp1, Mad2l1, Reps1, Reps2, Tia1, Ap3s1, Bbip1, Aqp4, Cry1, Pim2, Kifc3, Arap3, Rabgap1l, Gorab, Iqsec2, Vps54, Arhgap26, Dbn1, Arhgap29, Arhgef12, Slc7a5, Rab13, Arl3, Armt1, Efhd1, Hmgb3, Atf1, Chd7, Gata6, Hoxb2, Hoxb5, Creb3l1, Ipo4, Atp8b1, Usp3, Bach2, Chmp4c, Cnot8, Cstb, Ebf4, Hsf2, Itsn1, Lsm1, Pdlim7, Prps2, Ptch2, Rad52, Rbbp4, Ube2d1, Ube2d3, Chfr, Banf1, Batf, Bcar3, Zc3h7b, Isy1, Rbmx, Snip1, Hnrnpdl, Bcl2a1b, Ccng2, P2ry2, Selp, Birc3, Il10rb, Blnk, Cplx2, Rint1, Tmed3, Fkbp14, Brd7, Pparg, Tfeb, Zbtb18, Zfp101, Btg3, Bub3, C1galt1, Cygb, Cacna1d, Cacna1e, Fas, Capn6, Dchs1, Ppp3cc, Rbbp8, Zranb2, Naip6, Pycard, Traf1, Cbll1, Cdc26, H3f3a, H3f3b, L3mbtl3, Mbtd1, Pcgf6, Rbl2, Oxtr, Gmnn, Ccl22, Cks2, Dmtf1, Orc4, Cep57, Med30, Cd28, Cd44, Tial1, Hp1bp3, Stmn2, Cidec, Gabpa, Zfp85, Zfp942, Zfp953, Gm12258, Polr2l, Wt1, Zfp398, Zfp74, Zfp866, Zfp932, Zscan29, Mfsd7a, Chordc1, Sell, Dffb, Tlr13, Clp1, Nrxn2, Cluap1, Rassf4, Ppih, Ppil3, Cnot7, Col14a1, Col15a1, Lamb2, Plod3, Cops3, Fbxl14, Hnrnpa3, Rbm45, Thoc1, Crbn, Crym, Fbxl7, Csnk2a1, Cstf3, Ctnna1, Flywch1, Fzd9, Klf2, Pcdh15, Prom1, Tesk1, Tshz2, Orc5, Ssbp1, Frg1, Lrrn4, Rbm3, Rnpc3, Snrpg, Snrpn, Tlr11, Tlr9, Cyp4f18, Rnf145, Uchl5, Cybb, Cyfip1, Cyp4f17, Dcp1b, Thoc7, Eif4e, Dek, Wdr12, Eif4a1, Elf2, Rprd1a, Srsf1, Thoc2, Upf2, Zcchc14, Ddx43, Rspry1, Fancb, Ncdn, Dlgap3, Kctd17, Dph1, Lsm5, Rad51c, Rnaseh2b, Sp140, Dnajc15, Dnajc24, Dnajc7, Lrrc27, Ubash3b, Dock5, Dpy19l3, Tmem160, Dus1l, Nkd1, Hspb1, Reln, Slc18b1, Hnrnpu, Ssb, Vim, Fbxo11, Il27ra, Naip2, Ppp2ca, Ppp2r5c, Pptc7, Rhoc, Rhod, Exd2, Rasip1, Zcchc8, Ggnbp2, Uhrf2, Fgd6, Tnr, Fndc1, Mapk11, Trim47, Wtip, Meox2, Lrp2, Ntrk2, Vangl1, Gata3, Pld2, Gmpr2, Iqsec1, Plce1, Rgs1, Rgs18, Tshr, Wac, Gpbp1l1, Kctd6, Tacc1, Gtf2a2, Med4, Zfp871, Zfp873, Zfp995, Zscan12, Sik1, Trdmt1, Nek9, Msc, Zbed4, Trp53inp1, Prpf4b, Rbm39, Lrrc29, Jdp2, Mtpap, Mageh1, Nod1, Tra2b, Txlng, Zrsr1, Nasp, S100a6, Tfam, Tspan18, Txndc5, Txndc9, Hspbap1, Mettl6, Pfdn1, Sf3b6, Ift172, Ift57, Pcdh12, Inppl1, Pik3cd, Syk, Inpp1, Inpp5a, Msl3, Peli3, Irak2, Irf1, Tspan32, Itih5, Morf4l1, Tubgcp3, Sh3bp5, Ldb2, Lgals1, Lilra6, Lin9, Lipe, Plvap, Ptpra, Nsmce1,

	Nsmce2, Slc20a1, Tjp1, Mettl1, Mkx, Mmp25, Phb, Plxnb2, Rgl1, Msl1, Zscan21, Mycl, Mycn, Rfc3, Spo11, Srsf3, Zfp410, Zfp658, Nfia, Snrnp48, Nid2, Ninl, Nos1ap, Psmd6, Pan3, Wif1, Orc3, P4ha2, Pacs1, Serpinb6b, Pear1, Prps1l3, Syt7, Sytl5, Poll, Prrc1, Tlk1, Rassf3, Rcn3, Tnfrsf13c, Sprtn, Rin2, Tyms, Unc5c, Runx3, Scn3a, Syt17, Stk40, Srsf11, Tada1, Usp1, Tcp11l1, Tgfa, Ube2cbp, Wwc2, 4921524J17Rik, Snx20, Fhl5, Itga7, Rexo5, Upp1, Bambi, Il2rb, Naip1, Phlpp1, Stard13, Yap1, Picalm, Atad1, Atp8b4, Bace1, Lyn, Plcg2, Ube2k, Wdfy4, Bcl2a1d, Bcl2l2, Srfbp1, Brk1, Rsbn1l, Camsap3, Cbx1, Sfmbt2, Sirt1, Ttll4, Ccl21a, Fap, Cd19, H2-M2, Cd74, Cd79b, Cdk2ap2, Gm10778, Smarce1, Zfp111, Ciita, Clec4e, Clec7a, Cnot2, Col16a1, Cr2, Ldlrad3, Eapp, Neu3, Gbp2, Fcer2a, Fcgr3, Pou5f2, Itm2a, Itgb1bp1, Rgs19, Gbp5, Gpm6b, Lgmn, Hprt, Prmt6, Usp33, Malt1, Igfbp7, Itga8, Kansl2, Tmem50a, Ppp2r2d, Stab1, Myo1f, Ncf1, Nabp1, Nlk, Serpinb9, Pecam1, Pias2, Prkce, Rfwd3, Sema6b, S100a4, Sec23a, Vamp1, Slc40a1, Stat4, Tnfsf13b, Tnfrsf9, Bach1, Arhgap11a, Arhgap9, Cacng7, Cul4b, Ccdc18, Ccnb1, Cma1, Tjp2, Vasn, Cxcr2, Ddx17, Trim30b, Med18, Hcls1, Il10ra, Inpp5d, Prpf3, Magoh, Taf3, Rap1gap2, Ndel1, Nthl1, Tnf, Rnh1, Tpgs2, Plekhf2, Camk1, Arglu1, Npdc1, Lyz1, Ing2, Rarb, Zfp872, Serpina3n, C3ar1, Cenpu, Olfml3, Rbm5, Ppp1r8, Erg, Ets2, Fcrls, Ffar4, Loxl2, Ythdc1, Icam1, Igfbp3, Il7r, Peli2, Lrrc20, Slc7a11, Plin3, Rbm4, Itpkb, Fasl, Btg2, Cpsf6, Cpsf7, Wfs1, Flt3, Cpne2, Snx8, Engase, Gtf2e2, Zfp189, Hnrnpl, Samhd1, P2ry12, Snrnp27, Tgm2, Eif4g2, Il9r, Pdk2, H2-Oa, Aldh9a1, Ino80c, Adam12, Kif1c, Fubp1, Ifi30, Ak6, Akirin2, Vps26b, Aplnr, Apol6, Arl6ip1, Rtn3, Mcm9, Atl2, Reep1, E2f5, Smg7, Klhl5, Dcaf10, Mfn2, Med10, Bcs1l, Gpd1, Caml, Trpm2, Caprin1, Cnot6, Cnot6l, Pcyt1a, Ccr5, Rmnd5a, Cd3eap, Wdr48, Rnf4, Znrf1, Dbf4, Paxbp1, Cdk17, Cep76, Cep41, Cep72, Zkscan4, Pla2g12a, Fip1l1, Gmcl1, Slc22a18, Cnep1r1, Cnr2, Snx7, Coro2a, Crebzf, Smndc1, Ctss, Srsf7, Dcaf8, Ptgs1, Fermt2, Vash1, Pdcd7, Dusp11, Dnajc1, Dnaja3, Ppp4r2, Dysf, Ect2, Eepd1, Ltbp2, Gbp8, Parva, Ggt6, Mbtps1, Gpd2, Hcst, Ly9, Hpf1, Pmm2, Il18r1, Il18rap, Kif13a, Tnfaip1, Lat2, Tlk2, Primpol, Swsap1, Mplkip, Tenm1, Slc25a14, Wdr41, Podnl1, Rbm7, Rcn2, Tdrkh, Ptges2, Ube2g2, Zcchc10, Ttc39b, Spib, Spry1, Tnxb, Adamts7, Pirb, Ncf2, Cd80, Rnf6, Batf2, Mndal, Bst1, Cd226, Fbxw2, Cdkn2aipnl, Clic4, Oas3, Cmbl, Tmcc2, Ly86, Enpp2, Zfp326, Tdrd7, Lax1, Haus1, Phldb2, Mgat5, Tmem64, Nuak2, Papola, Plek, Wdyhv1, Trim3, Satb1, H2-Aa, H2-Eb1, Bzw1, Gfpt2, Clec12a, Gid4, Purb, Fbxw8, Msr1, Nln, Il15, Lrif1, Retn, Sh2d2a, Prpf40a, Prpf40b, Pnpt1, Rnf168, Ttc14, Uqcc2, Cenpi, Lin52, Siglecg, Rubcnl, BC055324, Btla, Fbxl12, Cdca4, Cep70, Fgfr1op2, Gpx7, Ppp1cc, Zfp511, Glis1, Sfxn5, Ifi203, Ccdc9b, Dbr1, Fam214b, Hnrnph1, Mgrn1, Ptdss2, Selplg, 1700037H04Rik, Serpina3c, Stx1b, Acp6, Paqr4, Cacna1i, Akap17b, Gpr18, Alox15, Tbc1d2, Mex3b, Dennd2a, Clstn2, Rps6kb1, Atg4c, Lamtor3, Ctla2a, Bnip2, Nudcd2, Bud31, Galnt7, Cadm3, Il33, Rarg, Cd38, Srgn, Cd86, Trim44, H2-M3, Scamp1, Cluh, Mier1, Cntf, Sned1, Swap70, Nelfa, Fgd5, Hsdl1, Ddx31, Dgke, Zfp330, Frmd3, Fzd1, Dtymk, Srp19, Loxl3, Glipr2, Tgfbi, Purg, Plcd3, Gpx3, Hr, Tal1, Rbmxl1, Klhl6, Nicn1, Timm10, Mettl8, Wdr89, Nudt21, Paip2, Ranbp9, Adcy7, Bfar, Ccdc93, Vpreb1, Mcm8, Isg20l2, Ms4a1, Topors, Tnfrsf13b,

	Tnfsf8, Chmp1b, Drg1, S1pr4, Zdhhc17, Plcb3, Slamf8, Arxes2, Clns1a, Draxin, Ndufb5, Rcbtb2, Trat1, Mettl14, Mta2, Slpi, Farp1, Ubap2l, Hnrnpll, Cenpn, Nxt1, Hnrnpf, Ddias, Hlx, Fcrl1, Rasa3, Il17rc, Rundc3b, Supt4a, Shisa5, Uhrf1bp1, Xpo4, Ing1, Slain2, Lrrc42, Cdkn2aip, Tmem79, Snx18, Fam122b, Sash1, P2ry13, Syce2, Fig4, Pik3ap1, Stard6, Nr1d2, B4galt4, Zrsr2, Thap2, Megf8, Osbpl2, Nudt16, Gimap3, Dpp7, P4ha3, Zbtb2, Col6a4, Pknox1, Alox5, Rel, Bicc1, Tmbim1, Nkain4, Lyz2, Bank1, Prr5, Rarres2, Cd53, Farp2, Timm21, Hs1bp3, Loxl1, Fgl2, Stard8, Taf1a, Haus2, Matr3, Mfsd9, Ube2d2a, Sdhaf2, Aloxe3, Slc44a4, Csf1, Icosl, Timd4, Naa30, Ptafr, Sarnp, Esyt1, Il4i1, Srpx2, Cst7, Nhsl2, Loxl4, Senp6, Cenpq, Nnat, Pigg, Stk10, Aoc3, Aida, Atg12, Atp9a, Tmem131, St13, Rusc2, Slc26a11, Irf8, Mtmr10, Ppfibp1, Atp13a3, Slc38a1, Ndc1, Cd48, Nsmce4a, Tiprl, Sulf2, Msl2, Zmpste24, Phf3, Lig4, Frem1, Ino80d, Tgs1, Morc2a, Prr13, Slamf7, Cdan1, Dok4, Son, Nfrkb, Mzb1, Cd33, Coq4, Ramp2, Retnla, Oxld1, Tekt2, Sfswap, Piezo2, Alox5ap, Tnks1bp1, Ly6a, Bckdha, C1qtnf2, Hsh2d, Phyh, Pcyox1, Cd200, Cd34, Pwwp2a, Mettl4, Ubl5, Abcb10, Olfml2b, Ubr7, Osgin1, Pou2af1, Ldlrad4, Gpbp1, Pxdn, Apol7c, C1qtnf1, Rhbdf1, She, Arl6ip6, Armcx5, Lix1l, Cd83, Mtfmt, Chst11, Cep95, Gpam, Gpr174, Hmgn2, Ccl19, Gm2564, Hvcn1, Slamf9, Tagap, Nkg7
Skin	Slc9a3r1, Abcc5, Sec24d, Sod1, Grk5, Acox1, Ndrg4, Sp4, Actr3b, Acvr1c, Il6ra, Smad5, Trim34b, Epha4, Jag1, Itgb6, Mpp7, Adamts20, Rras2, Frmd4a, Srms, Kif18a, Prkd1, Folh1, Plk4, Sdk1, Ifnar2, Ilk, Tgfbr3, Aldh1a1, Alx4, Rgs7, Cbx2, Cdk14, Ifit1bl2, Angptl3, Ankrd46, Ankrd50, Anks4b, Calr, Tapbp, Arrb1, Gm10263, Hspa12a, Rps12-ps3, Cdh6, Ephb1, Ror2, Tmed10, Aqp7, Cblb, Dusp6, Fgg, Fn1, Gdf11, Map4k4, Spry4, Trio, Arap2, Fgd1, Plxna2, Elmo2, Lgals8, Musk, Plxna1, Plxna4, Trrap, Arhgap32, Smarcd2, Arl13b, Rnd1, Asb11, Utrn, Tnfrsf1b, Ckap5, Cpn2, Gm5616, Zfp821, Aspm, Ass1, Atf3, H6pd, Pidd1, Rrm2b, Atp6ap2, Atp7a, Taf7, Ctps, Mcm4, Bard1, Bcap31, Bcl11a, Slc25a10, Lgr6, Lrrc39, Otub2, Prdx4, Usp43, Vdr, Bmp1, Bmp2, Braf, Chd4, Gli3, Gm14419, Ikzf2, Kat6b, Maml3, Mettl7a1, Ube2l6, Zbtb37, Zfat, Zfp280b, Zfp507, Zfp532, Zfp786, Jarid2, Setbp1, Cpsf2, Cd9, Myb, C1ra, Serpinb6a, Cachd1, Cap2, Casp2, Casp8ap2, Phyhd1, Stac, Mycbp2, Phc3, Ccdc47, Cdk10, Ccr1, Pfdn4, Cd81, Cdk15, Cdk5rap2, Cdkn3, Rbl1, Crebl2, Gli1, Trib2, Dynll2, Serpinf1, Serping1, Polh, Topbp1, Cit, Clasp1, Mark1, Epas1, Clu, Cmya5, Csf1r, Pcolce, Plod1, Sparcl1, Gtf3c2, Creb5, Mecom, Pcdhb14, Zfp105, Lrrc1, Lrrc9, Nono, Sik2, Csnk1g1, Ctnna3, Klhl4, Cux1, Gpr173, Ezh1, Dars2, Rrm1, Dcc, Prdx6, Taok3, Dnajc22, Dnhd1, Pard3b, Dnmt1, Men1, Zfp563, Lypla2, Poln, Dusp1, Synpo2, Dusp3, Dusp4, Ecd, Edn1, Efnb2, Egfr, Ehhadh, Eif4a3, Srsf9, Eif4ebp1, Eif4h, Elovl2, Entpd3, Epha2, Wnt5b, Mapk13, Exosc4, Utp3, Fasn, Zfp623, Fbxw7, Fcgrt, Mrto4, Surf1, Fli1, Hoxd8, Lef1, Fsd2, Fth1, Fzd6, Gja1, Ifrd2, Mrps2, Gm10053, Gm10184, Pkm, Khdrbs3, Nras, Rap2b, Rasl12, Rcn1, Mrpl52, Mrps33, Nip7, Gng11, Gpx8, Mt2, H2-Q7, Ube2i, Hdac1, Ran, Rpa3, Hes6, Hip1, Peg3, Zfp266, Hmcn1, Thap1, Xpo5, Zfp217, Zfp27, Zfp711, Hsd11b1, Msx1, Hspa1l, Tgfbr2, Slc16a1, Mical2, Mmp28, Tnc, Lrp12, Sh3rf1, Kank2, Katnal2, Kazn, Kcna1, Kcna6, Kctd5, Meaf6, Kif20b, Prickle2, Ldb3, Plekhg1, Lmod1, Lnx1,

	Pan2, Meis1, Nos1, Mogat2, Mrpl20, Mrpl36, Mrpl39, Mrpl46, Mrpl57, Mrpl9, Pla2g15, Nkd2, Npr3, Trim11, Nr2f2, Trim66, Nup107, Rcbtb1, Txnrd3, P2rx4, Phactr4, Scin, Tiam1, Pir, Plxnb1, Ptpn13, Ppip5k1, Ppp1r12b, Tbccd1, Prkab1, Prkci, Rasgrp2, Ulk3, Yeats4, Ywhab, Zfp384, Zfp46, Prss53, Psmb1, Pycr2, Rassf2, Trim63, Snrpa, Rgl3, Sfrp1, Rnf113a2, Rpl3l, Rtca, Sap30, Sass6, Scn4a, Siae, Slc25a48, Smad1, Zfp467, Tro, Snrpc, Spry2, Taf4b, Yeats2, Wnt5a, Trp63, Usp31, Ror1, Fkbp3, Ace, Actl6a, Pcdhga3, Pde4b, Adcy2, Nwd1, Ahsa1, Ak5, Itpr1, Aurkb, Bmi1, Cdk2, Paxip1, Plk1, Ankib1, Ankle1, Ap4m1, Hspb2, Arhgap6, Arid2, Asb13, Drp2, Zfp185, Asb5, Nrcam, Ash2l, Atad5, Rem1, Ccni, Kcnip2, Ptprq, Speg, Tmpo, Usp54, Pknox2, B4galt2, Ndufaf5, Gabarapl1, Mertk, Blm, Brca1, Brca2, Hivep1, Prdm15, Zfp114, Zfp236, C1qc, Coprs, Cct7, Cenpa, Cfp, Hells, Polr2h, Rad51, Setdb1, Zkscan6, Gstcd, Pbx2, Pcdhb2, Timm22, Eif1, Snrpe, Tlr2, Dlat, Fgf1, Syn3, E2f1, E2f8, Efna2, Efs, Eif4a2, Eno3, Ercc6, Ercc6l, Fads2, Fcgr1, Spire1, Foxc1, Gde1, Gm20695, Hsd17b11, Mmd, Gngt2, Gns, Rac2, Uck2, Has3, Nfe2l3, Hexa, Ttf2, Hspa14, Ntf3, Pard6g, Ikzf1, Iqank1, Irs1, Itgb2, Kdelr3, Ncoa2, Ppfia1, Tubg2, Larp7, Lig1, Lig3, Neo1, Map2k6, Mapkap1, Nek4, Trim45, Troap, Uap1, Rad54b, Timeless, Zfp422, Zfp473, Zfp568, Pycr1, Rragb, Slc25a5, Srgap1, Tbc1d5, Rrs1, Wasf1, Adora2b, Agt, Bub1b, Ccna2, Ncaph, Nphp3, Ube2c, Angpt2, Apex2, Arl11, Bbs2, Fgd2, Fgr, Ikbke, Efcab11, Asb1, Asb16, Pdzrn3, Smtnl2, Insig1, Tbx21, Csf2rb2, Mras, Sgk2, Prdx2, C130026I21Rik, Ccl5, Cd79a, Eomes, Mybl2, Zfp30, Cib2, Clspn, Cnksr2, Cxcl13, Dpp4, Hspa2, Ptgir, Dclre1c, Pus7, Sdf2l1, Grip2, E2f7, Eng, Ngfr, Fgf9, Sfrp5, Gp1ba, Kcns3, Ikzf3, Il21r, Mrpl21, Rab17, Nfkbie, Senp8, Ostc, Ppp1r3d, Rassf9, Rab39, Rad54l, Zfp78, Zfp931, Snrpd2, Trim30a, Sec61b, Slc38a2, Rab3ip, Ezr, Acadsb, Sqstm1, Gnaq, Pitrm1, Ptgis, Robo1, Actr1b, Capn3, Polr3a, Bmp7, Itgb3, Ppm1g, Tie1, Tnfrsf10b, Trim27, Btnl9, Tnfrsf22, Kdr, Trim25, Prdx3, Agpat2, Hscb, Lpin3, Pbx1, Cct3, Ahr, Ddo, Akirin1, Ar, Arl15, Becn1, Dlc1, Ksr2, Ptpn18, Tek, Gusb, Aldh18a1, G3bp2, Aldh1a3, Alg2, Rhpn2, Alpl, Amfr, Dlg2, Magi1, Gas2l3, Ttc32, Anln, Fat1, Padi4, Sele, Ap2a2, Reps1, Shh, St14, Arc, Syt4, Aqp4, Kif23, Arfgap1, Arhgap26, Racgap1, Arhgef15, Slc7a5, Sorbs2, Epha1, Tesk2, Blmh, Arpc4, Arv1, Ascc2, Atad2, Crem, Atg4a, Atp10a, Atp5h, Sorbs1, Aurka, Fbxo8, Hspb6, Lsm1, Ptch1, Ptpn3, Trim36, Ube2b, Bcar3, Prim1, Snip1, Smarcc1, Birc5, Il10rb, Bmp4, Iscu, Cdc20, Chaf1a, Tfec, Raly, Bub1, Stim1, Fkbp7, Cemip, Car1, Car7, Ewsr1, Mical1, Prickle3, Rab22a, Sfn, Oxtr, Gmnn, Ccnb2, Cdk1, Tchp, Tonsl, Irf2bp2, Klhdc8a, Cct5, Cct6a, Cct8, Cd276, Cd28, Cd44, Cdc6, Cdk4, Msi1, Incenp, Kif18b, Kif22, Cenpe, Cfap20dc, Cgn, Chchd3, Gatad2a, Wt1, Chek1, Mfsd7a, Id2, Cldn1, Cldn4, Klrb1a, Kif24, Kifc5b, Cnbp, Ppih, Exoc6, Col25a1, Lama1, Commd2, Copb2, Rbm45, Crbn, Crhr2, Crym, Fbxl7, Epcam, Gpsm2, Prom1, Scube3, Siah2, Sox18, Tgm1, Tle3, Naa38, Prelp, Cybb, Dctn3, Dctn5, Ddx11, Etv4, Ilf3, Nanos1, Homer2, Lsm6, Dock3, Dtx1, Fbp2, Elk3, Tpx2, Eme1, Pbk, Erc1, Ergic1, Igf2, Il27ra, Map3k6, Mapk9, Naip2, Nmi, Ppm1k, Esco2, Etnk1, Extl3, Procr, Fabp5, Fam241a, Fbxo5, Fcna, Fgf13, Fkbp11, Hoxa5, Hoxa3, Meox2, Jak1, Vangl1, Wnt11, Fubp3, Gabra3, Gabrr2, Umps, Nek2, Pask, Pde6d, Gipc3, Gla, Osgepl1, Golgb1, Gpd1l, Kctd6,

	Tacc1, Tsfm, Hhat, Homer3, Hoxc4, Hoxd4, Hunk, Myo15, S100a1, Srxn1, Trpm1, Txn1, Stmn1, Htra3, Trappc11, Txndc5, Hspbap1, Pfdn1, Igdcc4, Pcdh12, Ly75, Pdgfb, Iqgap2, Iqgap3, Irf1, Sufu, Mtdh, Kcnh1, Shcbp1, Sh3bp5, Lmo2, Mastl, Melk, Mest, Mier2, Rgl1, Sema3e, Tiam2, Usp6nl, Mrpl18, Mycl, Mycn, Pif1, Sp6, Spo11, Mylip, Napsa, Ncald, Ndc80, Tfdp1, Nrp1, Nrp2, Nsl1, Trip13, Tinagl1, Wif1, Orc3, Tfpi, Pla2g4b, Samd5, Sema6d, Pole, Sae1, Traip, Vapb, Prss12, Wfdc17, Zfp69, Syde2, Scn8a, Stx18, Spint1, Tmem177, Slc7a7, Smim20, Ston1, Tcp11l1, Tgfa, Tgoln1, Tnfrsf12a, 2310011J03Rik, Kcnj14, Ghr, Itga7, Bambi, Gypc, Ppp1r3c, Mical3, C1qa, Rps6kl1, Gab1, Lyn, Wdfy4, Cast, Cdh3, Faf2, Ttll4, Ccdc141, Cd79b, Grap, Zbtb46, Klrc1, Col12a1, Col4a5, Nampt, Vcam1, Dct, Ptchd1, Dll4, Man2b1, Etnppl, Fmnl1, Fnbp1l, Gja5, Prmt6, Ints7, Kbtbd12, Mcm7, Ncapd3, Ncr1, Nme1, Serpinb9, Pdha1, Pnma1, Trpv3, Ralgps2, Slc26a10, Arhgap11a, Rai14, Foxj2, Jmy, Car12, Ccnb1, Cdc25c, Clec10a, Tjp2, Nfatc1, Smap1, Hcls1, Kif11, Kif15, Lmnb1, Magoh, Man1b1, Med7, Phax, Rbpj, Slc39a2, Uhrf1, Sephs1, Arhgap19, Kif14, Kif1b, Serpina3n, Fbxl20, Cd302, Cenpk, Colgalt2, Slc22a3, Eva1c, Icam2, Igfbp3, Nuf2, Lilra5, Med15, Nr3c2, Pcolce2, Pop1, Rcc2, Tspyl1, Stk11ip, Srl, Ambra1, Hmmr, Hnrnpl, Kif20a, Prc1, Sash3, Prdm1, Trpv4, Siglech, Tgm2, Top2a, Samsn1, Hadhb, Accs, Gstp2, Setd3, Tagln3, Myo9b, Kif4, Pde4a, Atp1a2, Angel1, Tpi1, Ankrd40, Ptpn21, Cd3d, Aldh1l2, Pdss2, Foxm1, Cep55, Arl6ip5, Atf7, Atp6v1g1, Fancg, Usp13, Mfn1, Pgam5, Siva1, Nde1, Dusp7, Fam83h, Gm10110, Cadps2, Unc45b, Cbfa2t3, Ccr5, Dbf4, Cdc7, Cdca3, Lzts1, Kifc1, Idh1, Cep152, Ces2e, Chaf1b, Thrap3, Usp39, Cnih4, Dok7, Cst3, Ctss, Fbxo4, Dtl, Klhl41, Fermt2, Dld, Fnbp1, Ica1l, Spic, Ect2, Ehd2, Fignl1, Exo1, Gm11639, Fnbp4, Parva, Heyl, Tgm4, Ip6k3, Kif2c, Tspear, Nipsnap1, Trim12c, Mlxip, Mnd1, Sec14l5, Tango6, Rgmb, Tacc3, Ppp1ca, Sav1, Rasal3, Sub1, Sgcd, Tnfrsf18, Ucp2, Igsf9, Coq2, Eif2b3, Arhgap15, Mcm6, Cdkn2aipnl, Cenpf, Chac1, Mki67, Cmbl, Mdfi, Kntc1, Ddit4l, Dlgap5, Dscc1, Ly86, Gbp7, Stx2, Il1rn, Cnpy4, Msr1, Knstrn, Mtmr12, Rsad2, Dmrt2, Spg11, Lin52, Gad1-ps, Plscr1, Gabbr1, Glp1r, Gpx7, Nmnat2, Ska1, Abcg3, Ifi47, Stil, Psip1, Itga1, Abcg2, Acot7, Avil, Aff3, Cds2, Slc4a11, Plp1, Dnase2a, Grin2b, Thy1, Atg4c, Gpnmb, Cntnap1, Fzd5, Bora, Bud31, Galnt14, Stac2, Car2, Pmel, Cd38, Celf5, Ckap4, Ptprb, Dnah1, Kif26b, Egfl7, Sema6a, Cpxm1, Fgd5, Slirp, Lpl, Zfp330, Rbm15b, Fzd1, Pard3, Etfdh, Fryl, Gpsm1, Zfp14, Nav1, Nat8l, Mrpl41, Zfp365, Nptxr, Paip2, Tnfrsf26, Tnfsf10, Phospho2, Vcan, Sema4f, Wdr47, Tnrc18, Tecpr2, Dsn1, Cdhr5, Mcm8, Ulk4, Ms4a1, Smu1, Otulin, Izumo1, Dph2, Zdhhc17, Clns1a, Hnrnpa1, Plscr2, Orai3, Capn12, Ndufb5, Gimap6, Psmg1, Mtmr2, Ticrr, Farp1, Ccdc32, Ppp1r16b, Gm5424, Bloc1s5, Cd52, Fry, Prr11, Tdg, Slc30a1, Fam178b, Prx, Spn, Ciapin1, Gnpnat1, Jkamp, Ptpdc1, Ceacam1, Slc35f6, Ifitm6, Tmem88, Abcb1a, Acy1, Ammecr1, Gca, Tmbim1, Bnc2, Cd53, Colgalt1, Dgka, Dnajc12, Gm13305, Mfsd8, Stard8, Myof, Slc22a4, Tbc1d25, Trim26, Mrps26, 1110059G10Rik, Ptafr, Pomt1, Hhip, Wdr62, Eogt, Nnat, Secisbp2l, Rgn, Arhgap44, Arid5a, Asl, Fam83g, Fbxo9, Irf8, Nudt13, Tor2a, Acpp, Cenpp, Sulf2, Fam136a, Eya3, Atxn7l1, Nat9, Frem1, Zfyve27, Nqo2, Fam151b, Mtx1, Def6, Dok4, Cfh, Cox6c, Ncapd2, Slc39a10, Agmat, Cp,

	Dusp14, Hnmt, Sertad4, Car8, Fgfbp1, Ypel4, Sox4, Stc1, Spaca6, Stox2, Creld1, Pou2af1, Ncapg2, Tmem219, Btbd11, Cldn5, Emcn, Unc5b, Slc25a43, Cpm, Bglap3, Fam135a, Ly6g6d, She, Cd93, Clec14a, Ssmem1, Tymp, Lpar5, 3110040N11Rik, Tmem204, Ankrd11
Muscle	Brd3, Hlf, Smarca2, Tef, Dzip3, Abcd1, Rit1, Wipf3, Abtb1, Syne1, Sec22c, Smap2, Araf, Dnajc10, Wdr1, Fbn1, Ndrg4, Zfp202, Zfp472, Zfp729a, Zfp790, Actr10, Actr8, Acvr1b, Nhlrc2, Stat3, Trim65, Trub2, Zfyve16, Epha4, Adam21, Itgb6, Mpp7, Adamts20, Rab8b, Agap1, Kif18a, Agfg2, Ago2, Ahcyl1, Srsf4, Rgs11, Rgs7, Ifit1, Ifit1bl2, Ifit3, Plk3, Rpap3, Trim24, Ttc33, Wdr37, Anapc4, Angptl4, Ankhd1, Chd9, Cycs, Oasl1, Rev3l, Ldlr, Tnks2, Gnao1, Jak2, Map3k8, Map4k4, Nck2, Ncoa1, Pdp2, Spry4, Stxbp5, Trio, Tfrc, Cyth1, Gsk3b, Irak1, Map2k3, Map3k11, Psmb10, Arhgap10, Elmo2, Exoc1, Gdi1, Leo1, Tnfaip2, Tom1l2, Arhgef2, Arid4a, Dpf1, Arl8a, Arl8b, Atp2b4, Arntl, Rxrb, Arpp21, Mfsd3, Cnppd1, Specc1l, Dnal1, Eif4b, Herc3, Igsf10, Lrrc17, Zfp84, Qrsl1, Creb1, Dbp, Macrod1, Mafb, Ubr1, Atg13, Ulk1, Atic, Gstm5, Rdh13, Atp5g3, Atp6v0d1, Oxa1l, Taf7, Ilf2, Mcm4, Zc3h18, Npas2, Per2, Auts2, Klrb1f, Babam1, Herc2, Bbx, Cul4a, Bcor, Zbtb43, Bet1l, Bhlhe41, Birc2, Dhx58, Ifih1, Pex12, Ppif, Smad3, Traf4, Uba5, Bmp2, Gstm1, Bnip1, Clock, Dhdds, Irf7, Sncaip, Taf2, Zbtb10, Zbtb11, Zfp146, Zfp462, Zfp507, Zfp566, Zfp654, Zfp667, Zfp719, Zfp972, Brd8, Dhx40, Lrrc23, Ppil4, Bzw2, Cab39, Camk1d, Casp4, Rhbdl3, Gja6, Cbfa2t2, Mycbp2, Ccdc47, Med13, Med23, Tspan4, Naa10, Lurap1, Srp72, Dusp8, Chkb, Fkbp5, Ttbk2, Wee1, Snapc1, Ciao1, Cdkn1a, Mdm2, Cenpc1, Cenpm, Top2b, Gzf1, Tpcn1, Cit, Clasp1, Uba2, Strip2, Cog4, Commd8, Ercc8, Flot2, Cpt1a, Hic1, Klf13, Pcdhb12, Pcdhb16, Pcdhb19, Zfp579, Zfp606, Zfp952, Crebrf, Cry2, Csf2ra, Csnk1g3, Rps6kc1, Ctbp2, Ctnnb1, Nop14, Ctu1, Dpp8, Hcar1, Zdbf2, Ezh1, Hmgxb4, Cyb5d1, Dhcr7, Fdft1, R3hdm4, Cyp2e1, Cyp2u1, D6Wsu163e, Dap3, Nmt1, Scd2, Dmwd, Zfp36l2, Ddi2, Ddx3x, Heatr1, Tceal1, Edem2, Gstt2, Dnmt3a, Glt28d2, Nup98, Dpm2, Dusp1, Hexim1, Echdc2, Ehbp1, Fbxo32, Hyi, Ints10, Entpd8, Ptpn4, Trim23, Hbegf, Gtf2h1, Esrra, Fam110b, Mmp15, Fbxl3, Mrps7, Fgf18, Ptpn11, Fkbp9, Ip6k2, Impact, Ftsj1, Gadd45a, Gmeb1, Mob4, Mrps5, Mxd1, Sf3b1, Tmem147, Ncbp1, Paip1, Pum1, Tfip11, Zmat3, Isg15, Rab11b, Rab39b, Rhoj, Rhou, Gnb5, Rgs6, Ric8b, Gnl1, Gpt2, Gstm2, Trpm4, Prokr1, Trim13, Gtf2f2, Pak2, Mt2, Hdac11, Hdac9, Rad50, Zfp266, Zfp334, Zfp574, Rcor1, Zbtb49, Zfp316, Zfp354b, Zfp672, Zfp677, Zkscan7, Hsd17b7, Hspa4l, Trak2, Wbp2, Ift46, Tgfbr2, Ing5, Ints2, Lamc1, Junb, Rnmt, Tada2b, Kank2, Meaf6, Kif3a, Ppfia3, Kifap3, Klc1, Klb, Tbx3, Klf3, Lcn2, Mmp12, Plekhg1, Lipc, Lpin1, Rnpep, Uso1, Pld1, Lsm8, Mafk, Map4k5, Trim14, Srebf2, Mitf, Mrpl12, Mrps24, Mrps27, Nebl, Myc, Stx17, Ncoa7, Prkacb, Nfix, Sugt1, Nup62, Txnrd3, Oasl2, Optn, Upp2, Tex10, Shmt1, Zeb1, Pck1, Rhbdd2, Pfn2, Phf6, Rnf44, Rngtt, Polr3e, Ptprn2, Prkx, Sun1, Zfp408, Zscan22, Zscan26, Prkra, Psmd14, Rab27a, Rassf2, Rbm47, Trmt11, Snrpa, Sfrp1, Rnf181, Xbp1, Rpusd2, Rsph1, Sh3rf2, Slc4a7, Smad1, Zfp300, Zfp65, Zfp212, Tfcp2l1, Tm7sf2, Tpst1, Trp63, Usp18, Zfand5, Map3k5, Actl6a, Adrm1, Aff1, Nup133, Nwd1, Usp38, Akip1, Klhl28, Amph, Bmi1, Paxip1, Samd1, Nup85, Appbp2, Vps39,

	Arih2, Ypel3, Ube3c, Rab24, Ecm2, Bcl6, Phf19, Bcl2l1, Atg5, Rdh12, Rpa1, Atrnl1, Clec4d, BC025920, Bclaf1, Gabarapl1, Nedd4, Rnft1, Ube2a, Gm10226, Rbm26, Cdc16, Nalcn, Hint2, Cdkn2c, Fos, Hells, Kat8, Pi4ka, Rnf40, Xrcc3, Zfp110, Zfp263, Ckm, Cln3, Gstcd, Tbc1d24, Zfp715, Zfp72, Fgf2, Tet2, Ddx20, Ltn1, Trmt1, Ryr3, Dnajc11, Doc2g, Katna1, Eif1b, Eif2a, Pwp1, Emc1, Pcna-ps2, Fads2, Fam117b, Fkbp10, Ift81, Klhdc1, Rufy4, Gfm1, Gmps, Glis3, Rac2, Uck2, Hck, Hdac5, Tspyl4, Zfp142, Zfp526, Hmga1, Hook2, Msh3, Nol11, Iqank1, Tmed8, Kctd9, Ppfia1, Ky, Uggt1, Zbp1, Man1a2, Map3k13, Sdc4, Tulp3, Mrm1, Nacc2, Stam2, Pdgfc, Pdzd8, Rabl2, Stk35, Zfp39, Zfp641, Zfp811, Zfp870, Zxdc, Rubcn, Smn1, Rnf39, Rrs1, Tle1, Trim37, Trappc12, Lonrf3, Adrb2, Wsb1, Ccna2, Ift88, Tbl1x, Ap1g2, Aqp1, Bbs2, Smtn, Dapk1, Ddx58, Mras, Oas1a, Rasl11b, Atxn2, Rnf19a, Mrpl19, Cdyl2, Efhc1, Clspn, Cog3, Gng2, Gpr65, Il18, Lpar2, Ptgir, Phf11d, Dusp10, Grip2, Mob2, Ngfr, Stx8, Pdcd2l, Gp5, Pgd, Mx1, Zfp383, Itgb7, Usp24, Lrrc32, Tal2, Nek7, Ngf, Nup50, Polr1a, Rreb1, Tinf2, Zfp949, Trim30a, Slc38a11, Zyg11b, Pim1, 2010315B03Rik, 2810021J22Rik, Aasdh, Gnl3, Klhdc10, Mybbp1a, Nfx1, Nop58, Pum3, Surf6, Utp15, Cnbd2, Gopc, Whrn, Abhd2, Myo1e, Osbp, Sqstm1, Apob, Snx25, Acvr1, Crkl, Chrd, Gpc4, Itgb5, Kif5a, Nhlrc3, Ppp1r15a, Rgs2, Spryd4, Trim30c, Trim39, Trim7, Wasf2, Adck2, Adck5, Vipr1, Vps18, Map1lc3a, Gadd45b, Eaf1, Marveld2, Ago1, Ahrr, Cisd2, Ppard, Ttll12, Gclc, Gsto1, Arl15, Cdkn1c, Dusp5, Kctd20, Mapk6, Plcb1, Ppl, Uxs1, Zranb1, G3bp2, Rhpn2, Nfil3, Nt5c3, Amt, Nbn, Lmo7, Micall1, Rabep1, Nfe2l2, Ola1, Rab20, Arhgef40, Ttc32, Cdc37l1, Ubox5, Col4a2, Hap1, Col5a1, Prkcd, Clstn3, Cntfr, Get4, Cry1, Nr4a3, Exoc3l2, Fchsd2, Arfgap1, Arhgap26, Bcl7b, Zkscan14, Daam1, Psd3, Armc2, Kat7, Cebpg, Atg4d, Reep5, Atp11b, Car14, Pik3c2b, Axin2, Bag1, Dnm2, Hsf2, Mcl1, Nqo1, Txnip, Wipi2, Chfr, Bahcc1, Banf1, Bbs10, Prim1, Ccng2, Hapln1, Birc3, Cplx2, Cops7b, Pinx1, Stat5a, Zfp169, Zfp956, Raly, Rbm34, Cacna1g, Car11, Cask, Casp1, Midn, Phc1, Rai1, Ccm2, Ccnb2, Cdk1, E2f2, Irf2bp2, Col4a1, Tnfrsf19, Chka, Nup210, Cenpe, Cfap20dc, Polr1c, Zfhx3, Chmp6, Tlr13, Cluap1, Cmpk2, Lcp2, Cnbp, Ntng2, Exoc6, Col11a2, Col5a3, Col6a1, Comtd1, Copb1, Irf2bpl, Stx3, Crhr2, Golph3, Etv6, Flcn, Rhbdd1, Frg1, Ephx1, Dcp1b, Edc4, Desi1, Gpkow, Palb2, Rpusd1, Noa1, Wdr4, Dnajb14, Dnajc2, Plaur, Dock3, Dpf2, Sema4b, Dtwd2, Ltbp3, Med26, Egfem1, Eid1, Rsph9, Emc4, Kdsr, Nars, Samm50, Eral1, Ppm1k, Ppp2r5c, Rnf8, Tnik, Esco2, Ip6k1, Tbl3, Extl3, Fam126a, Ttc7, Ttc7b, Fastkd2, Tk2, Ppox, Fgd3, Fntb, Jak1, Fzd7, Hars, Gabrr2, Grem2, Pask, Gpr27, Nupr1l, Zscan12, Xpc, Hinfp, Mkks, Plekhf1, Shq1, Trpm7, Txlng, Zrsr1, Tbk1, Nasp, Sox9, Htra3, Tfam, Lingo1, Thop1, Pfkl, Ica1, Ift172, Igfbp2, Pdgfb, Pik3cd, Iqgap3, Nlrp12, Thumpd2, Mad2l1bp, Pex10, Sar1b, Marf1, Nub1, Melk, Zscan21, Mycl, Ppp1r10, Sntg2, Ntmt1, Prps1, Prps1l3, Rell1, Pold3, Rnf115, Tmem203, Ppp1r15b, Prrc1, Vapa, Sprtn, Zfand2b, Sugp2, Tbcb, Ston1, Ttc1, Zswim6, Acox2, Htatip2, Ipo8, Afg3l1, Als2cl, Ppp1r3c, Ide, Pmm1, Hoxa1, Atad1, Bace1, Gab1, Bcl2l2, Srfbp1, Sirt1, Ces1d, Cnnm3, Col6a2, Krt18, Elp5, Lemd2, Ovca2, Foxp1, Zdhhc24, Icam4, Inpp5e, Ints7, Peli1, Klhl26, Klra2, Zer1, Wdr27, Myo1f, Myoc, Ncapd3, Neil2, Neu2, Ppm1a, Pdk4, Tbc1d20, Sec23a, Tmed7, Ube2w,

	Tle4, Apobec1, Cul4b, Ctdp1, Ddx60, Ext2, Smap1, Lmnb1, Taf3, Mtmr14, Ndufs2, Tnf, Zfp710, Uhrf1, Dtd2, Pik3c3, Jmjd6, Ap3s2, Apex1, Spc25, Ube2f, Poc1b, Yipf2, Rarb, Serpina3n, Med9, Slc37a2, Cyp51, Dnttip2, Eid2b, Foxk1, Gemin8, Gm9845, Ttc30b, Lmbr1l, Nr3c2, Rabggtb, Rnf122, Ythdf3, Kcnj2, Atp2c1, Flt3, Egln3, Fbxl4, Gfod2, Hmmr, Prc1, Tbcd, Nudt16l1, Uri1, Ska3, Rhbdl1, Yod1, Top2a, Ogfod1, Mthfd1l, Samsn1, Mfsd1, Acer3, Ino80c, Setd3, Agrn, Arpc5l, Adam9, Psmd7, Kif4, Nt5c2, Kif9, Tom1, Angel1, Aldh1l2, Tmtc4, Ankrd13b, Cep55, Taco1, Pyroxd2, Tbc1d17, Armc10, Asph, Rara, Atl2, Tigar, Yipf5, Zmynd11, Fanca, Fancg, Fchsd1, Bcas3, Bcs1l, Dusp7, Orai2, Cnot6, Plekhh3, Mmachc, Rmnd5a, Cdk17, Cenpb, Klf9, Fip1l1, Cilp, Cisd3, Clec16a, Dnm1l, Thrap3, Ppp1r2, Spsb3, Tbc1d31, Coq9, Cox10, Slc25a12, Ndufc2, Ubxn1, Cuedc1, Klhl21, Klhl22, Cystm1, Paip2b, Sars, Srsf7, Dtl, Mettl2, Noc2l, Decr2, Phf5a, Dmxl2, Dnajc1, Mapkapk5, Ndufa11, E430018J23Rik, Zfp655, Orai1, Oma1, Pdcd4, Esrp2, Exo1, Ext1, Rdh14, Fam76a, Fcho1, Igfbp5, Gatad1, Snupn, Ggt6, Gpn3, Thap11, Rtn4ip1, Pmm2, Ptpn12, Lyar, Nfya, Kcnu1, Tspear, Ssc5d, Letm1, Tpk1, Man1a, Wipi1, Vamp5, Mrpl47, Nadsyn1, Ppp1r3f, Relt, Slc25a30, Tacc3, Pdcl, Tyw5, Pm20d1, Zc3h4, Ppp2r3a, Rbm7, Stk25, Tdrkh, Trmt6, Sbf1, Tbc1d12, Rnf38, Spry1, Strada, Coq2, Agl, Hlcs, Ube4a, Mndal, Pfkp, Ccdc91, Slc2a6, Chac1, Dctn6, Ddit4, Tdrd7, Fyb, Gbp7, Haus1, Tmed1, Parp11, Mcm3, Olfm2, Papola, Patz1, Plekhm1, Abcg1, Hrk, Trim16, Cbx7, Gfpt2, Slc41a3, Ndufa4l2, Zfp668, N4bp2, Mtfp1, Osbpl8, Mycbpap, Tnrc6c, Tbc1d8b, Rsad2, Lin52, Abat, Acot2, Cdca4, Rad9a, Zfp747, Traf3ip2, Serpine1, Zdhhc7, Steap3, Tlcd2, Ddx41, Kcp, Tbc1d2, Necap2, Klk8, Astn2, Atp6v1c1, Atp6v1a, Aven, Bnip3, Bora, Nudcd2, Galnt14, Capn15, Ttbk1, Cluh, Cnp, Sned1, Cox4i2, Tppp3, Lpl, Ptcd3, Fbxo44, Gstm6, Klhdc8b, Xaf1, Pde7a, Manba, Rsph4a, Sh3d21, Exd1, Toe1, Tmem216, Amd1, Hnrnpll, Ctsl, Ddias, Fancf, Pacsin3, Tax1bp1, St3gal4, Slc22a15, Yipf1, Bbox1, Dcun1d3, Cdkn2aip, Celf1, Coa5, Dut, Sash1, Slc39a6, Lpgat1, Ciart, Fzd4, Pla2g6, Abca9, Lox, Sec16a, Nudt16, Rfx5, Smdt1, Slc35f6, Zc3h12c, Alox5, Extl1, Bcam, Gck, Pacsin2, Cfap410, Rab3gap1, Arsg, Ssr2, Slc36a1, Acadm, Mtmr11, Mks1, Tctn1, Poglut1, Alg14, Bloc1s6, Cox19, Nt5dc1, St7l, Trp53inp2, Fbxo9, Isca1, Slc26a1, Ypel5, Gjb1, Cdc42ep2, Brd1, Pyurf, Pigm, Mid1ip1, Brpf1, Nubpl, Tstd3, Cep162, Tmem209, Rnf217, Sertad3, Amigo2, Cnnm4, Cntd1, Ccdc134
Liver	Hlf, Tef, Aars, Abhd6, Jund, Mapk14, Pdgfra, Abr, Syne1, Igf1r, Cnn3, Actg2, Lrg1, Adamts3, Adcy1, Gnb4, Nmrk1, Cxcl1, Ahctf1, Ahcyl2, Ahnak, Akap10, Prkch, Foxo1, Nos2, Tgfbr3, Plbd2, Pgm5, Alg3, Nr1i3, Rgs16, Eml1, Tbl1xr1, Trim24, Fgfrl1, Ankrd26, Calr, Foxo3, Rps26-ps1, Apba1, Psen2, Dkk3, Rab5b, Fkbp2, Gdf11, Grb10, Mapk15, Pdp2, Rasd2, Rasgrf2, Spry4, Stat2, Synrg, Traf3, Arhgef19, Dock11, Gorasp2, Map2k3, Rab11fip4, Copz1, Dnaja1, Ngef, Rab3il1, Sh3pxd2a, Sipa1l2, Arhgap24, Smarcc2, Arid4a, Arl4c, Atp2b4, Usp2, Arpc2, Vav2, Vamp3, Cnppd1, Dhx29, Igsf10, Lrrc59, Lrrc8b, Slit3, Tpbg, Tril, Rad23b, Aspm, Vcpkmt, Dbp, Pou2f1, Atg14, Ubl7, Mlh1, Rdh13, Atp6v0d2, Lhpp, Dclre1a, Npas2, Baiap2, Bcl11a, Bhlhe41, Casp12, Dhx58, Ephb3, Ern1, Gys2, Htra1, Lgr6, Rab8a, Uba5, Bmp2, Boll, Gm14288, Ikzf2, Kat6b, Mettl7a1, Nfatc4,

	Ppara, Zfp386, Zfp518b, Zfp68, Zscan20, Brdt, Cd9, Btbd10, Cwc15, Serpinb6a, Cyb5b, Cars, Casp4, Cav2, Kdm2b, Cdk10, Cct6b, Cd63, Trib1, Dusp8, Tanc2, Tubb4b, Cdkn2d, Crebl2, Hnf4a, Polk, Gzf1, Slc9a6, Chrna7, Cideb, Clcn7, Clec3b, Thrb, Gdap2, Cnn1, Cpeb4, Cpt1a, Mlxipl, Nabp2, Nedd9, Zfp324, Zfp426, Cry2, Per3, Scly, Cux1, Dhcr7, Cyp2a5, Fdx1, Gorasp1, Ddx27, Denr, Dgkd, Gstt2, Nucb1, Pdia3, Gch1, Dyrk2, Ears2, Echdc1, Eci2, Eea1, Eef1g, Eefsec, Eif5a, Enpp1, Spta1, Eya2, Fasn, Fcgrt, Plcl1, Surf1, Flt1, Flt4, Frem2, Fzd8, Gadd45a, Gbp4, Itm2b, Gk5, Gmeb1, Map2k7, Mrps2, Mrps5, Mxi1, Rpl13, Stt3b, N4bp2l1, Trim35, Gm12166, Gm14296, Smarcd1, Snrpd1, Snrpd3, Rbm28, Rala, Rcn1, Rhebl1, Rhou, Ubqln1, Zfyve1, Ppic, Psmc1, Gnb5, Gnai1, Gnmt, Got2, Gtf2ird1, Hdlbp, Zfp113, Zcchc9, Pdcd6ip, Ier3, Igfbp4, Tgfbr2, Rab31, Mical2, Jam3, Rnf135, Rnmt, Kazn, Kcnc3, Myo3b, Prickle1, Klb, L1cam, Larp1b, Pld1, Lrrc55, Slc25a1, Stap2, Pqbp1, Mcm10, Pdcd6, Mcu, Notch3, Minpp1, Mocs1, Mrpl36, Mrpl38, Mrps27, Myo1b, Naglu, Stx12, Vti1a, Nid1, Nr6a1, Nrf1, Nupl2, Tpm3, Uap1l1, Oasl2, Pck1, Pcsk4, Recql, Tiam1, Tox4, Pms2, Poli, Polr3c, Ptpro, Tbxas1, Ppp2r5a, Sun1, Zfp697, Zfp865, Zkscan2, Psmd11, Psme1, R3hcc1l, Wls, Rapgef4, Ube2l3, Rgl3, Rpl4, Tars, Rtca, Runx1, Siae, Slc37a4, Slc38a3, Tfe3, Zfp438, Zfp605, Tprkb, Srcin1, Tmem45a, Tm7sf2, Tmx4, Ubxn4, Map3k5, Ace, Actr6, Adam19, Adcy2, Usp38, Samd1, Prickle4, Ankrd24, Crip1, Csrp1, Ankrd37, Kpnb1, Asb13, Ccnd1, Dst, Atp8b2, Mphosph8, Shc4, Usp54, B4galt2, Cd40, Gabarapl1, Bsg, Hpn, Coprs, Tceanc2, Cep78, Scarb1, Cfdp1, Rev1, Rxrg, Zfp263, Zkscan6, Rab27b, Trib3, Npc1, Ston2, Tbc1d24, Cpt2, Klf10, Matn2, Pcdhb2, Pcdhb7, Klhl32, Dhrs7, Larp1, Dnah7b, Sarm1, Ehmt1, Txndc16, Gfm1, Glul, Gnl2, Slu7, Gns, Grb14, Mgl2, Pusl1, Prdm6, Ttf2, Spag4, Hsph1, Pard6g, Id1, Ikzf1, Reck, Tmed8, Kdelr3, Ppfia1, Spg20, Kin, Lgals9, Lhx6, Sdc4, Tmem14a, Tmem41a, Myo1g, Scfd2, Nek4, Phgdh, Ptdss1, Rasgrp3, Tsc1, Utp6, Zfp1, Zfp109, Zxdc, Vegfb, Rab37, Tgif2, Acly, Adora2b, Agtrap, Sgsm1, Mad2l2, Sgtb, Ube2c, Angpt2, Ikbke, Arl5c, Gmfb, Asb1, Flnc, Helz2, Mr1, Atp2b2, Dapk1, Ddx58, Jazf1, Avpr1a, Hes1, Ccr2, Chrna4, Cog3, Col4a4, Polr2g, Ctsc, Il18, Lsp1, Sdc3, Frmd4b, Dhx33, Pdia4, Evl, Grip2, Tubb2a, Edar, Elovl5, Eng, Inhba, Osbpl5, Tirap, Sphk2, Xrcc5, Golph3l, Lnx2, Gsn, Pgd, Hif1an, Sigmar1, Ifngr1, Itga5, Kank3, Slc7a2, Lasp1, Usp24, Lpxn, Map3k12, Mat2a, Nek6, Pnkd, Pcdhb5, Ppp1r3b, Zbtb24, Rab34, Shank2, Siglec1, Tpm1, Srpk1, Stx11, Top1mt, Trim21, Was, Dapk2, Hspa4, Pum3, Tfpi2, Acsl4, Acsbg1, Myo1e, Acaca, Osbp, Lipg, Ptgis, Robo1, Corin, Laptm4b, Bcr, Btnl9, Cd320, Erbb3, Hsp90aa1, Lefty1, Nedd4l, Peg12, Rhob, Rnf152, Tec, Trim7, Plekha5, Adamts1, Adcy3, Pde9a, Agpat2, Ampd2, Txnrd1, Gclc, Arhgap35, Cdkn1c, Dgkb, Erc2, Insr, Met, Nr2f1, Plcb1, Ptk6, Ptpn18, Stk24, Clpx, Gusb, Ormdl1, Rdh10, Ppp2cb, Amotl2, Nfil3, Angptl2, Lrp4, Arhgef25, Ola1, Anln, Sdcbp, Aox1, Clhc1, Cntfr, Pim2, Arfrp1, Arhgap28, Arhgef15, Sh3d19, Sorbs2, Epha1, Sipa1l1, Atad2, Hmgb3, Kat7, Cebpg, Hoxb2, Usp3, Pkp1, Pomk, Bag2, Eps8l2, Hid1, Mlkl, Ndfip1, Psmd12, Trim36, Txnip, Uchl3, Isy1, Ttf1, Bcl2l11, Clec2d, Fkbp14, Klf16, Zfp956, Zmiz1, Zcchc3, Ski, Bub1, Celsr1, Casp1, Traf1, Dnmt3b, Suv39h1, Lrrc8c, Lrrc8d, Cd44, Rassf8, Chka, Hp1bp3, Stmn2, Cyb561, Cidec, Ces2c, Zfp810, Lactb2, Mfsd7a,

	Sec23b, Dffb, Cisd1, Snx4, Cldn4, Tuba1c, Tuba4a, Clp1, Cluap1, Rassf4, Syt1, Ntng2, Col14a1, Lamb2, Col25a1, Crtap, Smoc1, Col6a1, Cpeb3, Irf2bpl, Stx3, Cpn1, Sertad2, Crip3, Csrnp1, Pcdh15, Ctnnd2, Rbm3, Rnf145, Cyp4f39, Ephx1, Cyp2a4, Slc17a8, Nanos1, Dffa, Ppip5k2, Rassf6, Dlgap3, Rnaseh2b, Ltbp3, Nkd1, Hspb1, Egfem1, Spen, Slc48a1, F8, Lmntd2, Vim, Nars, Rars, Erbb4, Ikbkg, Ppm1k, Pptc7, Tnik, Ip6k1, Zcchc8, Hgf, F3, Fam126a, Fgd6, Rnf225, Haus4, Gramd3, Slc25a20, Ripk3, Gfm2, Gpr39, S1pr5, Tshr, Golga5, Tmem200a, Gpatch11, Gsr, Gucd1, Trp53inp1, Hoxc4, Slc1a2, Psmd1, Tuba8, Psmc2, Sox9, Lrp2bp, Tmtc2, Lamp1, Scarb2, Ogdhl, Pcdh12, Pik3cd, Pik3cg, Ptpn22, Ptpn7, Ptprj, Syk, Itga4, Pdlim4, Mfge8, Klf12, Mag, Smc1a, Magi3, Slc20a1, Plxnb2, Usp6nl, Tmsb4x, Zfp658, Mylip, Ndc80, Ninl, Psmd8, Nt5c, Trip13, Pi4k2a, Pf4, Rnf144b, Tfpi, Plb1, Pole, Ppil6, Sntb1, Smc4, Rassf3, Rin2, Rnf150, Runx3, Stx18, Slc39a7, Zcchc18, Ythdf1, Tgfa, Abcd2, Acox2, Gsta1, Clic1, Stard13, Ide, Arcn1, Bin1, Arhgef3, Pgm3, Bdh2, Bloc1s4, Hps5, Gas1, Mvd, Psmc4, Faf2, Cbr4, Cenpj, Zbtb46, Tm9sf1, Usp8, Ciita, Clcc1, Clec11a, Klrb1b, Cpeb2, Ctsk, Dis3l2, Foxf1, Zdhhc24, Havcr2, Zpbp, Ifnar1, Il12rb1, Inpp5e, Irgq, Stab1, Nek10, Mmgt2, Mmp23, Slx1b, Pcdh20, Ppp1r18, Pecam1, Smc2, Tbc1d20, Sec23a, Rai14, Myzap, Tjp2, Gabrq, Setd4, Polr3d, Id3, Il10ra, Inpp5d, Kif11, Taf3, Mrc1, Slc39a2, Adcy6, Dhrs9, Apex1, Lgals3, Pam, Zfp872, Snca, Cerkl, Cyp51, Rbm5, Fcgr2b, Erg, Tab2, Ffar4, Sp2, Kynu, Mfsd2a, Mia3, Plin3, S1pr1, Tspyl2, Dtd1, Kif20a, Samhd1, Nif3l1, Prdm1, Tmem9, Yod1, Top2a, Pdk2, Mfsd1, H2-Oa, Xpr1, Psmd7, Kif4, Kxd1, Csad, Angel1, Iars, Mpi, Golga4, Rtn3, Armc10, Sgpl1, Myl6, B4galnt1, Gale, Pja2, Parp3, Caprin1, Dis3l, Rnf4, Cep135, Klf9, Fip1l1, Eif1a, Tbc1d31, Coro2a, Ndufc2, Cst3, Ctsh, Ubl3, Gnpat, Dld, Dnajc1, Ugdh, Fignl1, Nmnat1, F8a, Fanci, Nfxl1, Gpat2, Haao, Mrpl16, Ip6k3, Mafa, Wnk4, Ptprk, Lat2, Mob3b, Mblac2, Me2, Pafah2, Rgmb, Rcn2, Rnf138, Vipas39, Tnfrsf18, Tnxb, Trim59, Arhgap15, Gpr132, Mllt11, Cenpf, Mki67, Cmbl, Kctd7, Slc11a1, Trp53bp2, Leprot, Patz1, Tob2, H2-Aa, H2-Eb1, Pcdhb6, Ap1g1, Hey1, Cenph, Dnah5, Glod4, Hmgcs1, Ap3b1, Cox6b2, Rubcnl, Abce1, Sfxn5, Ap5s1, Serpine1, Stil, Pdzd4, Avil, Cds2, Agtpbp1, Ttll5, Mex3b, Sgip1, Mppe1, Clstn2, Ctso, Klhl18, Marcks, Il33, Rrp15, Lpcat1, Tbrg1, Ptprb, Kif21a, Snx5, Tma7, Swap70, Panx1, Glipr2, Ffar2, Fgf11, Slc6a17, Lancl1, Gpihbp1, Prepl, Mroh2a, Nicn1, Mdga1, Zfp365, Nrros, Nus1, Plaa, Vps37d, Adcy7, Dock4, Rimklb, Sh3d21, Ace2, Pon3, Vasp, S1pr4, Plekhm3, Capg, Cyp20a1, Insc, Spcs1, Mgat1, Rdh11, Slc15a4, Mars2, Ilvbl, Degs1, Sfr1, Rasa3, Ing1, Lpgat1, Sgms1, Nusap1, Prx, Cers5, Plekha4, Stard6, Entpd2, Mxra8, Nudt16, Ceacam1, Psen1, Ak4, Ammecr1, Aph1c, Thsd4, BC035947, Gck, Trabd2b, Cc2d1b, Ccdc77, Cpa3, Fbxo10, Fbxo38, Cxxc5, Fam107a, Loxl1, Hpse, Tspan15, Ldhd, Mab21l3, Trim26, Rexo2, Ptafr, Il4i1, Aqp11, Nipal1, Steap4, Nmb, Endou, Pomt2, Nptn, Gjb1, Morc3, Lyl1, Jade1, Phf3, Hbb-bt, Pigyl, Sdf4, Ndufaf7, Fstl1, Pmf1, Slc35a1, Tmem59, Nubpl, Setd6, Ldb1, Pdcd2, Slc8b1, Arsj, Azin1, Bckdha, Abca6, Adamts5, Slc7a6, Slc39a1, Mettl4, Sema7a, Slc35e2, Smim3, Adprh, Tctn3, Ptgfrn, Ubr7, Selenoh, Rnf128, Pram1, H2-DMb1, Shld1, Morc4, B3galt4, Dctd, Slc25a44, Ldlrad4, Sh2d5, Ccdc39, Atraid, Unc5b, Scaper, Dap, Tmx2,

	Slc39a3, Gpr153, Tymp, Gramd4, Parp16, Cd83, Gon4l, Gpam, H2-DMb2
Adrenal glands	Tef, Rras, Syne1, Igf1r, Sod1, Araf, Acsf2, Gm14410, Actb, Trim65, Aif1l, Pcid2, Alb, Plbd2, Trim24, Ttc33, Hspa12a, Rev3l, Aplp2, Tmed10, Fgfr4, Fn1, Grb10, Ikbkb, Tfrc, Axl, Asap2, Copa, Musk, Pld6, Smyd4, Atxn2l, Cops6, Dbp, Atic, Ppm1d, Atp6ap2, Triap1, Cul4a, Bhlhe41, Csk, Nudt12, Pea15a, Src, Traf4, Zfp260, Zfp458, Zfp59, Zfx, Dnd1, Tmx3, Rhbdl3, Cbr2, Cd209b, Ppp2r2b, Cdk5, Klf5, Clstn1, Lamc3, Stag2, Crk, Per3, Dcakd, Dcdc2b, Dmwd, Derl1, Dis3, Dnajc13, Stx1a, Dyrk2, Echdc1, Fam120c, Ptprr, Ier2, Smarca4, Zmat2, Rab11b, Rap1b, Sord, Hectd3, Sec23ip, Hes6, Prpf38a, Tgfbr2, Ncstn, Plg, Tnc, Rasgrf1, Junb, Kazn, Kif20b, Srsf5, Lrp1, Mavs, Med12l, Usp45, Slc25a15, Stoml2, Relch, Sema3c, Setd1a, Timm17b, Yy1, R3hcc1l, Rnf181, Sec63, Zfp128, Zfp160, Zfp748, Zfp869, Srcin1, Wrnip1, Acsl1, Aif1, Itpr1, Klhl28, Amotl1, Ankle1, Cd40, Zfp646, Masp2, Tmem165, Polr3h, Zfp110, Slc2a4, Dlat, Sarm1, Scube1, Myo1h, Egr1, Faf1, Pah, Gnaz, Gss, Hdac3, Ranbp3, Pard6g, Itpa, Lat, Mospd1, Mrm1, Myl1, Mylpf, Nfkbiz, Pcsk1, Tbc1d5, Siglece, Lonrf3, Aco2, Zfp944, Adipor2, Ift88, Bbs1, Cdc42bpa, Gmfb, Mast4, Arrdc4, F10, Cdyl2, Ddx5, Grhl3, Ikzf3, Kansl1l, Oas2, Nek7, Pex5, Srpk1, Hspa1a, Hspa1b, Npr1, Pim1, Unc13a, Nfx1, Utp14b, Cldn15, Prkar2a, Ccnd3, Acat1, Acat2, Osbp, Snx25, Bmp7, Dido1, Lefty1, Robo4, Tec, Tmeff2, Trim7, Cyfip2, Akt2, Nfil3, Ola1, Gas2l3, Cd69, Aox1, Chrm2, Mad2l1, Pcx, Hook1, Bbip1, Vps11, Exoc3l2, Gosr2, Arhgap4, Slc7a5, Zkscan14, Armc2, Blmh, Ipo4, B630019K06Rik, Cdc14b, Efcab2, Hspb6, Pgm1, Syde1, Trim36, Txnip, Efemp2, Slit1, Rbmx, Ddb1, Tmed3, Bnip3l, Cdc20, Tfap4, Gm14326, Rbm34, Cep83, Il16, Canx, Celsr1, Evpl, Ewsr1, Rab22a, Rbbp8, Cenpe, Zfp959, Zfp810, Gm2a, Sell, Cmpk2, Col7a1, Fbxl14, Ctsd, Fbxo2, Ssbp1, Rbm3, Zfand2a, Usp19, Fancb, Helq, Slfn1, Dnajc15, Dnajc2, Hcn3, Esm1, Slc48a1, F8, Entpd4, Rnf8, Procr, F3, Faap24, Ttc7, Polb, Fmnl3, Hoxa5, Tsc22d3, Jak1, Itk, Ramp3, Gramd1a, Gramd1b, Gsap, Pip4k2a, Hinfp, S100a1, Smyd1, Txlng, Wdr90, Myl6b, Pmpcb, Klrk1, Nasp, Sox9, Stmn1, Timm44, Zpbp2, Txndc9, Lamp1, Rdx, Socs1, Nup62cl, Itga11, Morf4l1, Kif7, Nsmce2, Mastl, Mon2, Mrfap1, Tbc1d2b, Zmym6, Ncald, Nsl1, Tinagl1, Osbpl11, Rnf115, Rnf146, Usp1, Timd2, Tmem63a, Ablim1, Sepsecs, Adap1, Cast, Ccp110, Usp8, Cr2, Dnah7c, Dpt, Exph5, Flad1, Mcee, Hopx, Tcof1, Kmt2b, Jmy, Abhd10, Aqp3, Elfn1, Eda, Elk1, Kynu, Slc45a3, Atp2c1, Taf8, Btg2, Ints1, Flt3, Yod1, Orai1, Igsf1, Zfp207, Pth1r, Pltp, Sema4f, Fam122b, Hhipl2, Arhgap21, Atg12
Colon	Hlf, Tef, A4galt, Jak3, Rasl11a, Rras, Appl1, Il6ra, Adam32, Prkch, C8a, Fga, Asap2, Cyth3, Exoc4, Leo1, Lrrk2, Per1, Lrrc49, Tsku, Aspm, Ythdf2, Dbp, Npas2, Per2, Bard1, Baz1b, BC034090, Bet1l, Bhlhe41, Ern1, Gys2, Rnf5, Clock, Smyd3, Zfp280b, Dusp23, Lrrc2, Casp2, Cct6b, Tspan4, Cdc27, Cdc42bpb, Wee1, Cdkn3, Spc24, Zfp955a, Cog4, Col24a1, Cry2, Per3, Cyp2a5, Dazap2, Scd1, Dcp2, Gstk1, Ddx10, Derl3, Zfp563, Klc4, Poln, Vps45, Dusp3, Edn1, Egfr, Fasn, Zfp623, Gle1, Mnt, N4bp2l1, Rala, Rac3, Peg3, Hp, Hsd17b4, Rnf41, Isoc1, Kng1, Mmp28, Mmp12, Lonrf1, Lrfn3, Mrpl15, Pelo, Prkab1, Xpnpep2, Taf1b, Ppp2r5d, Pcdhga3, Actr6, Aif1, Aurkb, Plk1, Atf2, Atn1, Trf, Ppm1l, Bmp3, Wdr19, Cfi, Uggt2, Rev1, Zkscan6, Fbn2, Matn4, Myo1h, E2f8, Ercc6l, Fabp7, Flii, Pah,

	Gngt2, Zfp526, Iqank1, Pdlim5, Map2k6, Mxd3, Scai, Nanp, Vtn, Rasgrp3, Smg5, Uvrag, Zfp39, Zfp689, Wnt2, Vta1, Bub1b, Ccna2, Rasl11b, Ccnf, Ccl2, Ccr2, Clspn, Lsp1, Ptgfr, Pus7, Dvl2, E2f7, Fcer1g, Oas2, Pdhx, Rab39, Rad54l, Serpina1d, Serpina3k, Serpina3m, Rpl18a, Rcan1, A430033K04Rik, A530032D15Rik, Tfpi2, Myo1e, Pnpla3, Tmem184a, Lefty1, Pten, Aldh3a2, Clic3, Bex2, Sult4a1, Nfil3, Plekhg5, Anapc11, Lrp4, Nfe2l2, Anln, Aoc1, Ap3s1, Rnaseh2a, Ccbe1, Cry1, Fchsd2, Kif23, Racgap1, Sh3d19, Tesk2, Ascc2, Lamtor1, Car14, Aurka, Itsn1, Rnf123, Bax, Bcas2, Bcl2, Cdc20, Brox, Bsn, Bub1, Calcrl, Ppp3cc, Ctbp1, Ccl22, Lrrc8c, Incenp, Kif18b, Kif22, Cfap20dc, Chek2, Tlr13, Csnk2a1, Vtcn1, Kmo, Zfand2a, Dctn3, Tpx2, Pbk, Mapk9, Fancd2, Gata3, Ripk3, Nek2, Pask, Kdm4a, Gpr39, Pdzd11, Pip4k2a, Rab6b, Wdr13, Klrk1, Stmn1, Traf7, Iqgap3, Plk5, Shcbp1, Mastl, Melk, Mmp25, Ppp1r10, Zbtb25, Pear1, Vapa, Nf2, Acad9, Snx20, Alox12, Pmm1, Cbr3, Clec4e, Trpv2, Mmgt2, Plxnc1, Pigk, Tmcc3, Adamts4, Fam83d, Tjp2, Med18, Kif11, Kif15, Lmnb1, Ypel2, Ankrd55, Arhgap19, Kif14, Ctc1, Slc22a3, Fcgr2b, Acss3, Ints1, Fbxl19, Fbxo25, Tomt, Kif20a, Prc1, Psd2, Yod1, Top2a, Mthfd1l, Cacna1s, Adam12, Kif4, Kif13b, Foxm1, Atp13a1, Fanca, Chmp5, Dcaf10, Mfn1, Dusp7, Kifc1, Gm2004, Klhl22, Ect2, Tor1aip1, Epg5, Nmnat1, Fanci, Mast2, Stbd1, Heyl, Nlrx1, Kif2c, Podnl1, Cdca8, Pkmyt1, Pfkfb3, Kntc1, Leprot, Tmed1, Cbx7, Cdca2, Chtf18, Mcrs1, Uqcc2, Tsc22d2, Mgrn1, Ptdss2, Plgrkt, Slc13a4, Sned1, Glipr2, Gpihbp1, Hepacam2, Ppfia4, Tnfrsf13b, Hba-a2, Gtse1, Tiparp, Slc22a15, Abhd15, Sash1, Prr11, Nusap1, Osbpl2, Postn, Ceacam2, Rab3gap1, Slc25a34, Slc13a5, Pnpo, Jagn1, Lipt2, Tbc1d22a, Nudcd1, Hbb-bt, Kcne4, Stard3, Pou2af1, St3gal1, Mmaa, Cc2d1a, Cldn8, Fam214a
Kidney	Pdgfra, Wipf3, Wdr1, Smad5, Itga6, Akr1c12, Crebbp, Rnf7, Ubc, Anapc4, Dpysl2, Ankrd46, Rad9b, Ap1b1, Heatr5a, Brd4, Tmed10, Ncam1, Igf2r, Cdkn2b, Pcbp2, Plxna4, Arfgap3, Grb2, Smyd4, Chd8, Atxn2l, Brpf3, Crnkl1, Nr2c2, Atg13, Atg14, Ulk1, Atp5g3, Lama2, Irf2, Ep300, Osr1, Smyd3, Zfat, Cpsf2, Zfp395, Phyhd1, Cherp, Itih4, Tspan4, Clasp1, Nr5a2, Snx9, Clu, Suco, Cpt1a, Rrm1, Scd2, Hmgcr, Ddb2, Gpatch2l, Dpm1, Eif4ebp2, Ep400, Lef1, Tpp2, Ubb, Psat1, Rbm28, Rrad, Nnmt, Neto2, Trak2, Isoc1, Myh11, Kansl3, Tipin, Scaf4, Setd1b, Stau1, Mcoln3, Mrpl43, Myc, Zzef1, Nupl2, Rpgr, Pigc, Psmd11, Sec14l4, Tars, Zfp438, Tnfsf15, Lrrc71, Ankrd10, Ap2b1, Lta, Asxl1, Baiap2l1, Nedd4, Cep78, Ehd4, Cse1l, Sfrp2, Zfp142, Pign, Rspo1, Mettl16, Txndc15, Ptrh1, Tbc1d8, Vta1, Slc25a5, Arrdc4, Atxn2, Rnf19a, Tomm20, Ehd1, Gpatch1, Ptgs2, Mkrn3, Manf, Rreb1, Serpina1d, Rps27l, Taf13, Ubtf, Actg1, Tube1, Polr2a, Rasl10b, Wasf2, Acvr2b, Aga, Clk1, Hspa13, G3bp2, Rab38, Parvb, Tmed9, Gbf1, Arhgef11, Calcoco1, Gata6, Vdac1, Bcorl1, Xpo7, Dchs1, Cdc26, Orc4, Ces2c, Ces2g, Gm12258, Tmem198, Cisd1, Kifc5b, Klf2, Dhx57, Rbm3, Cyfip1, Eif4e, Palb2, Gtpbp8, Elmod3, Rhoc, Mphosph6, Glg1, Prrc2b, Gabrr2, Trpm6, Gpm6a, Gtf3c4, Rfk, Ubr2, Wdr13, Tsacc, Mettl6, Ldb2, Lrat, Mon2, Mrfap1, Zbtb40, Mycn, Ncoa6, Ndufb9, Tnfaip3, Tmem63a, Sptlc1, Pxk, Yap1, C1s2, Samd8, Arid1a, Plcg2, Rsbn1l, Cd7, Cept1, Def8, Zpbp, Myoc, Cd2, Cd300lf, Id3, Ist1, Foxk1, Podxl, Hsd3b2, Casc3, Cxcr6, Fbxl4, Id4, Rph3al, Yod1, Acer3, Mypn,

	Dlst, Aldh1l2, Tmtc4, Stac3, Unc50, Npepps, Tigar, Hnrnpul1, Svil, Cytip, Zfp358, Cox8a, Ramp1, Enc1, Ltbp2, Rtn4ip1, Il18r1, Wnk4, Mpp1, Polr3gl, Zfand1, Ttc39b, Tvp23b, Ccdc50, Clic4, Pfkfb3, Plekhm1, Zbtb39, Cox6b1, Il15, Utp23, 1700037H04Rik, Praf2, Zfp553, Syngr1, Kdm3b, Kansl1, Bfar, Izumo1, Adam33, Gtse1, Srrm2, Cdkn2aip, Abca9, Rnf149, Ncmap, Gnpnat1, Megf8, Slc30a5, Sema3g, Lypla1, Lamtor5, Ubiad1, Slc25a34, Dipk2a, Haus8, Slc25a39, Abhd17b, Arsk, Cox7a2, Pigf, Ptgr2, Pigo, Pomp, Morc4, Sertad3
Lung	Tef, Tfcp2, Pdgfra, Rasl11a, Gzmm, Gna13, Gucy1a2, Zbtb3, Adam17, Adcy8, Macf1, Fat4, Amdhd1, Ttc12, Angel2, Dpysl3, Ppp2r5e, Smarca1, Rhobtb1, Leo1, Tgfbrap1, Rad54l2, Arntl, Rorc, Lrrc49, Asxl3, Dbp, Atic, Triap1, Atp8a2, Hnrnpa0, Npas2, Bhlhe41, Vdr, Zfp146, Zfp239, Ppp4r4, Ccnh, Ccno, Tspan4, Naa60, Ppp2r2b, Wee1, Cdkn1a, Runx1t1, Topbp1, Cspg4, Hsd17b1, Plekhh1, Ptpn11, Fzd3, Gata2, Srpr, Rerg, Rhebl1, Rhoj, Ric8b, S1pr3, Plekhg1, Lnx1, Lrrc55, Ssh2, Mapre3, Rasl2-9, Ppip5k1, Trim17, Rplp1, Sh3rf2, Zfp438, Ntpcr, Amph, Angptl7, Arfgef1, Arhgap6, Dock7, Nyx, Wdr70, Rnaseh2c, Rev1, Rbms1, Ddx21, Fat3, Jmjd1c, Rrp8, Tulp3, Nacc2, Ung, Rubcn, Styk1, Sspn, Mmp14, Wdr77, Zfp184, Sdccag8, Ednra, Ednrb, Kiss1r, Pus7, Gas2l1, Zbtb14, Gpatch1, Mrpl30, Pou4f1, Podn, Rassf9, Ppp1r26, Trim21, Prkar2a, Gnaq, Lipg, Gdf10, Lefty1, Spryd4, Dgkb, Ltb4r2, Dlg2, Nfil3, Ttc16, Angptl2, Antxr2, Shh, Rbbp5, Ccbe1, Arhgap29, Psd3, Rasef, Gata6, Trappc4, Bach2, Znrf3, Lrrc8e, Kalrn, Cyb561, Plod3, Flot1, Cox15, Cysltr1, Nanos1, Lsm6, Vipr2, Negr1, Extl3, Faap24, Narf, Mmd2, Trappc11, Htr2a, Sp6, Ncald, Ntan1, Pcdh11x, Pcdh19, Rrh, Tbc1d30, Bmper, Itga7, Pde7b, Adra1a, Ccp110, Clint1, Dusp28, Gbp2, Fnbp1l, Gbp5, Mme, Ptpn2, Plxnc1, Ralgps2, Tcp11l2, Lrrfip1, Cyp26b1, Il2ra, Micall2, Spns3, Poc1b, Fh1, Lmo4, Sema4d, Rabggtb, Xlr, Pgk1, Ank, Uqcrfs1, Cpeb1, Dnajc28, Nmnat1, Heyl, Tnfaip1, Ptprk, Raver2, Znhit6, Gbp7, Pitpnm3, Plek, Coq6, Mcat, Tmco4, Abce1, Cep70, Tars2, Pi15, Gtpbp10, Orai3, Nr1d2, Spaca1, Aldh7a1, Elp4, Ccdc40, Ppfibp1, Arhgap17, Phyh, Plekha2, Tbkbp1, Amigo2, Mfsd2b
Spleen	Tef, Mmp17, Adrb3, Nup93, Rgs11, Mta1, Spry4, Tyro3, Arl13b, Foxs1, Dbp, Phf11c, Mdp1, Npas2, Vezf1, Zfp955b, Cad, Ccno, Cd209f, Pdgfrb, Col3a1, Cpox, Zbtb41, Shb, Per3, Mpst, Zfp36l2, Ddah1, Eftud2, Eif2s2, Sgta, Mmp15, Ier2, Smarcd3, Mcu, Mrpl17, Nin, Nup107, Rcbtb1, Pex7, Ptprn2, Tor1aip2, Sf3b3, Xbp1, Sec63, Zfp467, Tubb5, Prkaca, Prickle4, Ankrd9, Ap1s1, Bcl2l1, Ndufaf5, Rnft1, Ccnl1, Nacc1, Pcdhb4, Zfp715, Efnb1, Gfm1, Hgs, Senp3, Rspo1, Lztfl1, Supt20, Ackr4, Casp3, Timm17a, Ehd1, Nmd3, Lnx2, Pou2f2, Metap2, Naa20, Nr1d1, Pou4f1, 1700109H08Rik, Mdm4, Noc4l, Pde9a, Ccnt2, Arf6, Clip3, Map2k4, Rab23, Angptl2, Ccdc8, Ano2, Cry1, Rab10, Atf6b, Mcl1, Mlkl, Shkbp1, Bnip3l, Zfp956, Pcgf6, Dmtf1, Ces2g, Cntn2, Ush2a, Cxcl12, Elf2, Rangap1, Dnaja4, Prdm11, Rhod, Sema4c, Rbm39, Hoxd4, Pcdh12, Pom121, Megf10, Mest, Ccl17, Hsf5, Cpeb2, Farsa, Slc2a12, Tcof1, Sema6b, Bak1, Rcc2, Snx10, Acss3, Anxa4, Trappc5, Arpc5l, Ak6, Gart, Tnk2, Camsap2, F8a, Ddit4, Plac8, Usp42, Acsm3, Hk1, Bmt2, Nudcd2, Kif26b, Lpl, Pdpn, Rogdi, Dcun1d3, Nr1d2, Tnfrsf11b, Hepacam, Cys1, Depdc1b, Tmsb15b1, Hcfc1r1, Leng1, Fbrs, Haus5

Eye	Abcc6, Rbks, Tab3, Frmd4a, Agfg2, Rpl5, Anapc10, Phip, Ldlr, Psmb10, Trrap, Arl8b, Myo9a, Cpn2, Lrrc3, Aspscr1, Atat1, Dbp, Pou2f1, Mbd1, Atp5a1, Atp5o, Clock, Zfp644, Bzw2, Cad, Cars, Kmt2c, Snx30, Dgkd, Nrip3, Dnajc13, Eef1g, Eif3m, Fah, Hras, Rpl36a, Pygb, Rpl17, Usp21, Med27, Minpp1, Sncg, Psmb1, Rfx3, Suz12, Spry2, Klhl28, Hspb2, Appbp2, Lrmda, Cops8, Pole3, Creb3l3, Dnah7b, Gm4787, Has3, Hivep3, Prdm6, Ldhb, Kcna3, Ppfia1, Vamp8, Nrg2, Aco2, Ccna2, Cd79a, Xcr1, Nfyb, Eef1a1, Helb, Rpl22, Rasgef1b, Zbtb32, Mapk3, Als2, Amot, Anapc2, Ano2, Bbip1, Sde2, Cfap20dc, Clcn5, Cluap1, Cspg5, Fzd9, Nudt4, Dph1, Mapk11, Scg5, Tsfm, Htra4, Pfkl, Ift57, Iqgap3, Lilra6, Star, Mgarp, Wdfy3, Ripk2, Itga7, Arfip2, Hps5, Klrd1, Lysmd4, Vti1b, Agmo, Kif14, Bak1, Fhod1, Pnpla6, Cpsf7, Myh2, Ryr1, Myh1, Itpr2, Atp5g1, Bid, Chmp3, Dhps, Kars, Trdn, Tyw5, Ppp1ca, Sv2a, Rasal3, Nek3, Sdhc, Kcp, Grm8, Mtch2, Cd27, Ddx31, Ghdc, Gstm6, Ufc1, Zfp14, Mettl8, Caap1, Mtap, Rpl37, Thap7, Sgpp2, Lamtor5, Rps15, Prcp, Dcaf15, Acot13

Supplementary Table 3. The edge list of DIN.

Node-1	Node-2	Differential count
Hnf4a	Npas2	36
Cdk16	Nfil3	33
Rbl2	Cdk1	33
Npas2	Vdr	33
Arntl	Usp2	32
Nfil3	Zfp521	31
Usp2	Per2	31
Wsb1	Hsp90aa1	30
Psmd12	Htra3	30
Gm10184	Cct2	29
Hcfc2	Wsb1	29
Nfil3	Hivep2	29
Top2a	Rbl2	29
Zfp623	Tef	29
Dctn5	Actr8	28
Acvr1c	Ntf3	28
Bcl6	Nfil3	28
Brca1	Per2	28
Rbl2	Ccna2	28
Rbl2	Cenpf	28
Nfil3	Elk1	28
Flt4	Insr	28
Rxrg	Npas2	28
Npas2	Strip2	28
Psmd11	Ubc	28
Ap1m2	Prkab1	27
Bace1	Tfrc	27
Fhod1	Trim23	27
Foxj2	Nfil3	27
Nfil3	Foxn3	27
Ube2i	Hspb1	27
Npc1	Slc2a2	27
Tfrc	Tpd52l1	27
Agrn	Pbk	26
Hfe	Brdt	26
Ccnjl	Rbl2	26
Ddit4	Snrpg	26
Dnm1l	Snx4	26
Epha2	Ppm1k	26
Fos	Nfil3	26

Nfil3	Foxj3	26
Per2	Hif1a	26
Hltf	Zfp949	26
Hspb1	Stmn1	26
Tbk1	Lefty1	26
Lrrc55	Slu7	26
Mef2a	Wsb1	26
Mrpl19	Tmem177	26
Smad3	Nfil3	26
Npas2	Psmd14	26
Pnkd	Paf1	26
Rbl2	Pbk	26
Psat1	Tubb2a	26
Rbl2	Trim59	26
Zfp217	Tef	26
Tef	Zfp454	26
Nr2f2	Actr10	25
Nf2	Actr8	25
Pdzrn3	Casp2	25
Lgr6	Cct3	25
Commd10	Wsb1	25
Cux1	Trim24	25
Cyb561	Eci2	25
Zfp90	Dbp	25
E2f6	Nfil3	25
Oxtr	Grk5	25
Hells	Ino80c	25
Herc4	Ube2f	25
Zfp708	Hlf	25
Igf1r	Hspa4	25
Rpap3	Hspa4l	25
Rhob	Itga5	25
Tef	Kat2b	25
Slu7	Lgr6	25
Mmp28	Serpina3c	25
Serpinb6a	Mmp3	25
Mxd1	Smad3	25
Zfp949	Nfic	25
Ppp1r3c	Ppp5c	25
Ppp2r5c	Raf1	25
Tef	Rad54l	25
Rsl1	Tef	25
Slu7	Slit3	25

Vdr	Snw1	25
Snx4	Tfrc	25
Actl6a	Adcy3	24
Cmpk2	Ak5	24
Brca1	Anapc4	24
Anapc4	Dmwd	24
Wdr20	Anapc4	24
Bet1l	Map1lc3a	24
Bhlhe41	Per2	24
Btnl9	Fos	24
Ccnb2	Rbl2	24
Dbp	Cdk18	24
Cdk1	Dusp7	24
Tef	Chd5	24
Lsm2	Crnkl1	24
Runx1	Dbp	24
Echdc1	Rpsa-ps10	24
Ern1	Tirap	24
Per2	Esrra	24
Wdr13	Ezh1	24
Fgfrl1	Fgf18	24
Foxp2	Tef	24
Nr2f2	Gnai1	24
Grip1	Serpinb6a	24
Prpf8	Gstt2	24
Itgb5	Rhod	24
Lnx2	Kit	24
Nfil3	Klhl24	24
Lims2	Lats2	24
Lgr6	Snw1	24
Lims2	Ppp1r16b	24
Map2k6	Rhoj	24
Nme2	Tubb2a	24
Tubb2a	Nme3	24
Tgfbr2	Nphp3	24
Wsb2	Per2	24
Phf10	Taf2	24
Ppm1g	Ptk6	24
Sil1	Rpl23a-ps3	24
Smyd2	Zfp949	24
Ube2e1	Sqstm1	24
Tef	Zfp109	24
Zfp52	Tef	24

Stat1	Aars	23
Pnpla3	Acsl4	23
Dnm1l	Actr2	23
Actr8	Cryab	23
Actr8	Synpo2	23
Hspa1l	Acvr1c	23
Acvr1c	Mink1	23
Agfg2	Pak2	23
Agrn	Pask	23
Ctsk	Ahr	23
Npas2	Ahrr	23
Tmtc4	Anapc4	23
Cdk1	Ankrd28	23
Rem1	Anln	23
Dbp	Atf1	23
Atf3	Trim2	23
Baz1b	Dbp	23
Etv6	Brca1	23
Bub1b	Tubb2a	23
Slit1	Cct2	23
Cttnbp2	Cct3	23
Tpbg	Cct3	23
Plk2	Cdk1	23
Tmod4	Chmp4c	23
Cpsf3	Slu7	23
Crip1	Itga5	23
Csf2rb2	Ptk6	23
Mylk3	Csnk2a2	23
Smn1	Cstf2	23
Dbp	Glis1	23
Dhx29	Nop14	23
Dnajb6	Rpusd2	23
Etv3	Tef	23
Tef	Foxo3	23
Ybx3	G3bp2	23
Gtf2h4	Polr3k	23
Hells	Zfp532	23
Hip1r	Parva	23
Ttf2	Hspb1	23
Ttc33	Ift172	23
Tef	Ikzf1	23
Kpnb1	Insig2	23
Lama3	Itga5	23

Itga5	Pmp22	23
Tgfbr2	Itga5	23
Kpna1	Per2	23
Map3k5	Pbk	23
Ywhaz	Map3k5	23
Serpinb6a	Mcpt4	23
Mdm2	Trim24	23
Med23	Naa15	23
Mrpl45	Tmem177	23
Mrps2	Rplp1	23
Rps10-ps1	Mrps2	23
Myo1e	Rad23a	23
Ptgs1	Ncbp1	23
Ncoa2	Sirt5	23
Smad4	Nfil3	23
Vezf1	Nfil3	23
Npas2	Nr5a2	23
Nr2f2	Trim24	23
Nr6a1	Tef	23
Ptk6	Ntf3	23
Pask	Rbl2	23
Snrpd1	Pdhb	23
Thrb	Per3	23
Plagl2	Rchy1	23
Smarcad1	Pld2	23
Rad23a	Trim23	23
Rad23b	Uba5	23
Zfp532	Rad54b	23
Rbl2	Smad2	23
Rhod	Sorbs3	23
Zfp872	Sap30	23
Zfp956	Sap30l	23
Smarcc1	Yy1	23
Tubb2a	Stmn1	23
Tfrc	Tbk1	23
Tef	Zfp108	23
Tef	Zfp46	23
Actr1b	Fos	22
Actr8	Clip1	22
Trim63	Acvr1c	22
Adcy1	Nme3	22
Adcy1	Rasl12	22
Cenpe	Agfg2	22

Aif1l	Tubb2a	22
Ankrd27	Psmc4	22
Anln	Itpr1	22
Apaf1	Foxo3	22
Nfil3	Atf1	22
Atp5o	Nt5c2	22
Bcl11a	Dbp	22
Stac3	Cachd1	22
Ccna2	Spast	22
Cct3	Cyfip1	22
Irak4	Cdkn1a	22
Chka	Pnkd	22
Chrna4	Itga6	22
Crkl	Elmo3	22
Cry1	Per3	22
Tia1	Crym	22
Cwc27	Ubl5	22
Vezf1	Dbp	22
Oas1a	Dmwd	22
Prkci	Dusp1	22
Mllt3	Eaf1	22
Ecd	Zbtb43	22
Insr	Efna2	22
Flt4	Egfr	22
Eif5b	Fos	22
Ran	Ephb3	22
Erbb2	Sh2b2	22
Ercc6	Yy1	22
Ercc6l	Tef	22
Ets2	Rbl2	22
Tef	Eya3	22
Fgfrl1	Ror1	22
Fosl2	Tef	22
Frk	Ppp2ca	22
Tef	Gfi1	22
Ggt6	Gpx7	22
Phf7	Gm14296	22
Gm9843	N4bp2l1	22
Gnao1	Mrpl19	22
Hells	Zfp949	22
Hira	Tubb2a	22
Zkscan6	Hlf	22
Rpa1	Hnrnpk	22

Hsp90ab1	Map3k5	22
Hspa4l	Wsb1	22
Ikzf5	Nfil3	22
Pik3r1	Il1r2	22
Pik3ca	Ing5	22
Itga8	Lamc1	22
Jak2	Raf1	22
Serpinb6a	Klk8	22
Lef1	Zbtb7c	22
Srsf9	Lgr6	22
Lrch4	Tirap	22
Srms	Lrp3	22
Wnt5a	Lrp4	22
Tnf	Lrrn4	22
Map3k5	Ndc80	22
Nasp	Mat2b	22
Wsb1	Mef2c	22
Rab10	Nfil3	22
Nup210	Ube2j1	22
Rbl2	Oas1a	22
Rnf144b	Oasl1	22
Tspan4	Ocln	22
Pik3cb	Tgfbr2	22
Ppard	Rxrg	22
Ppm1g	Tgfbr2	22
Tnf	Psmd10	22
Sec23b	Psmd14	22
Rev1	Ubc	22
Vapb	Rsad2	22
Tef	Runx3	22
Rxra	Trim24	22
Sdc4	Sqstm1	22
Yy1	Setdb1	22
Zfp467	Setdb1	22
Tef	Thrb	22
Tef	Zfp169	22
Tef	Zfp2	22
Tef	Zfp473	22
Tef	Zfp93	22
Traf3	Tifa	22
Tmtc4	Wsb1	22
Ubr1	Ube2w	22
Ubqln1	Wdr77	22

Acap2	Rab20	21
Ppbp	Ackr1	21
Aco2	Tfrc	21
Myo1e	Actr1b	21
Myo1e	Actr3	21
Chd3	Actr8	21
Coro6	Actr8	21
Actr8	Tuba8	21
Acvr1c	C130026I21Rik	21
Acvr1c	Irs3	21
Adam33	Itga3	21
Enpp3	Adcy1	21
Kif4	Agfg2	21
Ahctf1	Piwil4	21
Shmt1	Ahcyl2	21
Hyou1	Aif1l	21
Ajuba	Capn3	21
Gmps	Aldoa	21
Mad2l1	Anapc4	21
Ube2w	Anapc4	21
Ankrd27	Cdk18	21
App	Cfb	21
Tef	Ar	21
Rasgrp1	Arl8b	21
Tbc1d17	Arl8b	21
Arntl	Eif3f	21
Atf3	Sh3rf1	21
Wdr38	Atf3	21
Ecd	Bcl6	21
Esr2	Bhlhe41	21
Fgfrl1	Bmpr1b	21
Npas2	Brcc3	21
Chd4	Brdt	21
Bud31	Snw1	21
Scarb2	C1s2	21
Ccni	Ppp1cb	21
Cct2	Ecm2	21
Cct2	Igsf10	21
Cct2	Rtn4r	21
Lrig3	Cct3	21
Cd209a	Ptpru	21
Cdc25c	Plk2	21
Irs3	Cdc42bpg	21

Cdk20	Gtf2e2	21
Cttnbp2	Cdk2	21
Cebpb	Fos	21
Gsr	Ciapin1	21
Clcn7	Trappc10	21
Npas2	Cops6	21
Tns1	Crkl	21
Dnmt3b	Cul4a	21
Dchs1	Egfr	21
Dcp2	Rps20	21
Ddx20	Hspa14	21
Dhx29	Ltn1	21
Trim23	Dmpk	21
Ttc33	Dmwd	21
Mtg1	Dnajb6	21
Hnrnpa1	Dnajc10	21
Dnmt3b	Il2rg	21
Dtx4	Ltbp1	21
Itgb6	Dusp10	21
Dusp14	Slc9a3r2	21
Tubb2a	Dynll1	21
Tef	E2f7	21
Lrch4	Eci2	21
Hace1	Epha2	21
Map4k4	Epha2	21
Esr1	Uba5	21
Esrra	Fos	21
Fabp5	Ube2f	21
Fcgr1	Insr	21
Fkbp3	Noc2l	21
Fos	Tef	21
Trim2	Fos	21
Irs3	Gab1	21
Gab1	Tnc	21
Gm10226	Hdac2	21
Gm14296	Hlf	21
Sqstm1	Gsk3b	21
Hcar1	Lnx2	21
Zfp687	Hdac2	21
Hdac2	Zgrf1	21
Heyl	Nr2f2	21
Zfp654	Hltf	21
Rbl2	Hspa14	21

Hspb2	Hspa4	21
Id3	Klf4	21
Pask	Ifnar2	21
Nfil3	Ikzf2	21
Imp3	Mrpl46	21
Imp3	Rpsa-ps10	21
Mef2d	Ip6k2	21
Irf7	Iqsec1	21
Irs3	Ptk6	21
Pabpc4l	Itgae	21
Kank1	Parva	21
Kif4	Plk2	21
Tef	Klf15	21
Klf16	Tef	21
Pdzrn3	Lgr5	21
Lgr6	Sf3a3	21
Lpin1	Prkcq	21
Map3k8	Lrrc39	21
Snw1	Maml3	21
Mcm7	Mat2b	21
Rem1	Met	21
Mfsd7a	Tnfrsf22	21
Serpina3c	Mmp3	21
Raf1	Mras	21
Yy1	Mta2	21
Mxd3	Mycl	21
Mxd3	Zkscan14	21
Mxi1	Zbtb42	21
Mxi1	Zbtb43	21
Myo1e	Osbp	21
Ncbp1	Snrpe	21
Zfp112	Nfil3	21
Notch3	Snw1	21
Trib3	Nr1i3	21
Nras	Trio	21
Oas1a	Nrip3	21
Pabpc1	Zcrb1	21
Ppard	Per2	21
Pfn1	Rbp7	21
Zkscan14	Phf11b	21
Ppfia1	Ptpn12	21
Tyro3	Ppp1ca	21
Rev1	Rad51b	21

Reck	Tgfbr2	21
Rnf165	Ube2j1	21
Tars	Rpl10	21
Rpl23a-ps3	Srp14	21
Snw1	Slit3	21
Wee1	Smurf1	21
Srf	Srebf2	21
Taf7	Tada1	21
Tnf	Tbk1	21
Zfp263	Tef	21
Tef	Zfp59	21
Zfp791	Tef	21
Zfp867	Tef	21
Tpbg	Txnl4a	21
Zfp956	Ube2d1	21
A430033K04Rik	Tef	20
Ctnnal1	Actl6a	20
Cap2	Actr8	20
Chrnb1	Actr8	20
Hspb6	Actr8	20
Actr8	Hspb7	20
Dst	Acvr1c	20
Acvr1c	Tgfbr2	20
Acvr1c	Trim2	20
Adam15	Itga5	20
Pdgfra	Angptl7	20
Smtn	Ankmy2	20
Anks4b	Plekha5	20
Anxa2	Dnajc8	20
Ap1m2	Ap3b1	20
Ap2b1	Hbegf	20
Appl1	Srms	20
Appl2	Stab1	20
Brdt	Arntl	20
Arntl	Ddx59	20
Arntl	Ndufaf5	20
Sfrp5	Arrb1	20
Igf1r	Asap3	20
Asb11	Plekha5	20
Snw1	Aspn	20
Tox4	Atf3	20
Atg13	Rheb	20
Npepps	Atl3	20

Atp5o	Ephx1	20
Atp8b2	Dnajc2	20
Bap1	Ncoa1	20
Rbl2	Bard1	20
Snip1	Baz1b	20
Irf1	Bcl11a	20
Srms	Birc5	20
Blm	Pik3cb	20
Pld2	Braf	20
Prdm15	Brdt	20
Brip1	Rpa1	20
Crip1	Calr	20
Gm27029	Calr	20
Calr	Prnp	20
Smtn	Cap1	20
Capn3	Syne2	20
Jup	Car7	20
Cask	Hdac2	20
Trim24	Cbx5	20
Ccnb1	Rbl2	20
Plk2	Ccnb2	20
Ccr3	Gna12	20
Cct3	Igsf10	20
Cct3	Prelp	20
Cct5	Hnrnpa0	20
Lrig3	Cct5	20
Tlr5	Cct6b	20
Ptpru	Cd209f	20
Cdc20	Uba5	20
Cdk20	Gtf2b	20
Cdk2	Rxrb	20
Parp3	Cdkn1a	20
Chek1	Pik3cb	20
Clock	Eif3f	20
Npc1	Clu	20
Gar1	Cnbp	20
Cnot7	Cstf2	20
Sncaip	Crkl	20
Irf2bp2	Csnk2b	20
Drp2	Ctnnal1	20
Topbp1	Cttnbp2	20
Cux1	Rbl2	20
Dnaja1	Cyb5d1	20

Tfrc	Dag1	20
Rhbdl3	Dapk1	20
Dbf4	Plk2	20
Lef1	Dbp	20
Nr2f6	Dbp	20
Taf5	Dclre1a	20
Xpc	Ddb2	20
Ddhd2	Urm1	20
Ddx20	Lsm2	20
Desi1	Kpna4	20
Eri3	Dhx58	20
Dmpk	Sqstm1	20
Dnajb11	Snw1	20
Dnajc2	Eif4g1	20
Dnajc2	Wsb1	20
Pkm	Dnm1l	20
Dusp1	Tbk1	20
Egln3	Egln1	20
Nr2f2	Eif4a2	20
Tiam2	Epha2	20
Kalrn	Ephb1	20
Erc1	Tyro3	20
Ppm1g	Ern1	20
Npas2	Esrra	20
Ube2f	Esrra	20
Ets1	Rbl2	20
Ets2	Mapk11	20
Rac3	Exoc3l4	20
Itga5	F10	20
Tmed2	F8	20
Hspa14	Fer	20
Fgfrl1	Fgf11	20
Hspa1l	Flcn	20
Rev1	Flna	20
Ube2i	Fosl2	20
Itga3	Fyn	20
Rem1	Gab1	20
Plk4	Gas7	20
Setdb1	Gli1	20
Zfp287	Glis1	20
Phf7	Gm10226	20
Gm14403	Smarcd2	20
Sash1	Gnas	20

Pparg	Gnat2	20
Gp1ba	Rpl4	20
Tceanc2	Gpn3	20
Mgst3	Gsto2	20
Gtf2h4	Sumo1	20
Ing5	Hdac9	20
Zfp27	Hells	20
Hells	Zfp507	20
Hells	Zfp956	20
Twist1	Hes1	20
Tgfbr2	Hras	20
Tef	Hsf2	20
Tubb2a	Hspa14	20
Id3	Runx2	20
Ubc	Ifrd1	20
Ppt1	Igsf10	20
Pik3c2b	Ing5	20
Irs3	Smg1	20
Pink1	Isg15	20
Ubqln4	Isg15	20
Itga5	Mmp23	20
Prg4	Itga5	20
Lims2	Kank1	20
Kank2	Ptk7	20
Zrsr1	Khdrbs3	20
Prickle2	Kif15	20
Lims2	Kif26b	20
Pag1	Kif5b	20
Lgr6	Tcp1	20
Lnx2	Lin7a	20
Srsf9	Lrig3	20
Lrpprc	Tbk1	20
Lrrc59	Ppp2cb	20
Lsm6	Snrpd1	20
Mst1r	Mag	20
Maged1	Paxip1	20
Txn2	Map3k5	20
Mat2b	Setdb1	20
Recql	Mcm10	20
Plk2	Mcm7	20
Meaf6	Taf3	20
Med4	Med18	20
Mrpl47	Ppib	20

Rps20	Mrrf	20
Ms4a2	Mst1r	20
Mxd1	Ppp1r12b	20
Pxdn	Ncbp1	20
Upf2	Ncbp1	20
Npas2	Smad3	20
Npas2	Smarcd3	20
Npas2	Trap1	20
Npas2	Uck2	20
Slc8b1	Npc1	20
Npr2	Plxna2	20
Trim13	Nr2f2	20
Nr2f2	Trim3	20
Nr2f2	Trip6	20
Srms	Nras	20
Nub1	Psme3	20
Ttc33	Oas1a	20
Pink1	Oasl1	20
Oasl2	Tmtc4	20
Pbk	Pdzrn3	20
Sec23a	Pdcd6	20
Serpinb6a	Pdzd2	20
Per2	Per1	20
Per2	Wdr13	20
Per3	Vrk1	20
Pik3r3	Ppara	20
Tef	Plagl2	20
Pld2	Pum1	20
Robo1	Podn	20
Tmtc4	Pola1	20
Pole3	Smarca1	20
Ppard	Smyd1	20
Ube2w	Ppil1	20
Rxrb	Ppp1cb	20
Tef	Prdm6	20
Prkcz	Robo4	20
Sele	Ptprj	20
Rad23b	Smyd5	20
Ube2e1	Rad51c	20
Ube2s	Rad51d	20
Rad54b	Rbl2	20
Topbp1	Rai14	20
Rbl1	Tef	20

Rbl2	Trim34b	20
Spry4	Ret	20
Rgs12	Smad7	20
Tom1	Rheb	20
Ripk4	Zfp507	20
Rnf19a	Vcp	20
Tars	Rps2	20
Rtn4r	Snw1	20
Tef	Runx2	20
Setd7	Wsb1	20
Zfp27	Setdb1	20
Smarcd2	Zfp467	20
Smarcd3	Zfp949	20
Tanc1	Smg1	20
Wee1	Spata13	20
Zfp119b	Tef	20
Tef	Zfp692	20
Zfp869	Tef	20
Tfrc	Zfp36l2	20
Wrnip1	Ubc	20

Supplementary Table 4. Summary of the NASA GeneLab datasets. *"/" in the last column denotes that the dataset contains samples with different doses.*

Dataset	Number of ground control samples	Number of spaceflight samples	Tissue	Dose (mGy) in spaceflight samples
OSD-47	3	3	Liver	4.784
OSD-98	5	6	Adrenal glands	7.752
OSD-99	6	6	Muscle	7.752
OSD-100	6	6	Eye	7.752
OSD-101	6	6	Muscle	7.752
OSD-102	6	6	Kidney	7.752
OSD-103	6	6	Muscle	7.752
OSD-104	6	6	Muscle	7.752
OSD-105	6	6	Muscle	7.752
OSD-137	6	6	Liver	8.97/9.2/9.471
OSD-162	5	5	Eye	8.97/9.2
OSD-163	6	6	Kidney	8.97/9.2/9.471
OSD-164	6	6	Liver/Spleen	4.784
OSD-168	9	9	Liver	7.752/8.97/9.2
OSD-173	2	2	Liver	4.66
OSD-194	4	5	Eye	9.471/9.702
OSD-238	6	12	Skin	8.84
OSD-240	10	10	Skin	7.14
OSD-241	10	9	Skin	7.14
OSD-242	4	5	Liver	8.295
OSD-243	19	18	Skin	8.49/14.999
OSD-244	19	19	Thymus	8.49/15.848
OSD-245	19	20	Liver	8.49/15.848
OSD-246	16	15	Spleen	8.49/15.848
OSD-247	17	19	Colon	8.49/15.848
OSD-248	19	20	Lung	8.49/15.848
OSD-253	19	20	Kidney	7.592/7.83/21.812/22.099
OSD-288	3	3	Spleen	6.4
OSD-379	35	35	Liver	6.446/11.115
OSD-401	6	6	Muscle	14.22

Supplementary Table 5. The radiation gene set.

**Radiation gene set**
Abraxas1, Aen, Atm, Babam1, Babam2, Brat1, Brca1, Brcc3, Brcc3dc, Clk2, Cop1, D7Ertd443e, Eya1, Eya3, Inip, Ints3, Kat5, Mrnip, Mta1, Nabp1, Nabp2, Nhej1, Paxip1, Rfwd3, Rnf168, Rnf8, Smpd1, Stk11, Ticrr, Topbp1, Uimc1, Usp28, Prkdc, Xrcc4, Lig4, Trp53bp2, Dclre1c, Rad54b, Rad54l, Xrcc6, Nek1, Bax, Bard1, Blm, Brca2, Cdkn1a, Clock, Ect2, Eef1d, Fignl1, Gadd45a, Grb2, Hus1, Ino80, Ints7, Mapk14, Net1, Rad1, Rad51, Rad51ap1, Rad9a, Rhno1, Rhob, Rpl26, Sirt1, Spidr, Swi5, Tank, Tnks1bp1, Trp53, Rad9b, Itgb6, Tgfb1, Snai2, Fbxo4, Bbc3, Kdm4d, Cdc25a, Col3a1, Ogg1, Plk3, Txn1, Polh, Mbd4, Fancg, Bcl2l1, Kit, Myc, Hsf1, Nscme3l, Nsmce3, Mtch2
